# Unraveling Translational Insights into Systemic Multi-Organ Toxicity of Cytosine Arabinoside (Ara-C): A Systematic Review of Preclinical Animal Evidence

**DOI:** 10.3390/cimb48010004

**Published:** 2025-12-19

**Authors:** Ioannis Konstantinidis, Sophia Tsokkou, Antonios Keramas, Kali Makedou, Eleni Gavriilaki, Georgios Delis, Theodora Papamitsou

**Affiliations:** 1 Department of Medicine, Faculty of Health Sciences, Aristotle University of Thessaloniki, 54124 Thessaloniki, Greece; stsokkou@auth.gr (S.T.); antonios@auth.gr (A.K.); 2 Laboratory of Biochemistry, AHEPA University Hospital, School of Medicine, Aristotle University of Thessaloniki, 54124 Thessaloniki, Greece; kmakedou@auth.gr; 3 2nd Propedeutic Department of Internal Medicine, Haematology Unit—Haemophilia Centre of Northern Greece, Hippokration General Hospital of Thessaloniki, Aristotle University of Thessaloniki, 54124 Thessaloniki, Greece; gavriiel@auth.gr; 4 Laboratory of Pharmacology, School of Veterinary Medicine, Faculty of Health Sciences, Aristotle University of Thessaloniki, 54124 Thessaloniki, Greece; delis@vet.auth.gr; 5 Laboratory of Histology-Embryology, Department of Medicine, Faculty of Health Sciences, Aristotle University of Thessaloniki, 54124 Thessaloniki, Greece

**Keywords:** cytarabine (Ara-C), systemic toxicity, neurotoxicity, intestinal mucositis, ocular toxicity, hepatotoxicity, nephrotoxicity, developmental toxicity

## Abstract

**Background/Objectives**: Cytarabine (Ara-C) remains central to acute myeloid leukemia therapy but is limited by unpredictable systemic toxicities. Preclinical studies have long documented multi-organ injury, yet findings remain fragmented. This systematic review synthesizes animal evidence to clarify the spectrum, dose–response patterns, and mechanisms of cytarabine-induced toxicity. **Methods**: Following PRISMA 2020 guidelines and PROSPERO registration (CRD420251081384), a comprehensive search of PubMed, MEDLINE, Scopus, Cochrane Library and Embase identified eligible in vivo animal studies. Data extraction covered animal models, dosing regimens, routes of administration, histopathological and biochemical endpoints and mechanistic findings. Risk of bias and study quality were assessed using SYRCLE’s tool, CAMARADES checklist and an adapted Newcastle–Ottawa Scale, with reporting benchmarked against ARRIVE 2.0. **Results**: Eighty-one studies (1964–2024) were included. Cytarabine produced dose- and regimen-dependent toxicities across multiple organs. Neurotoxicity was most frequently reported, followed by intestinal mucositis, ocular injury, alopecia, hepatotoxicity, nephrotoxicity, and developmental anomalies. Mechanistic analyses consistently implicated oxidative stress, inflammatory cascades, apoptosis, and epigenetic dysregulation. Study quality was moderate, with frequent deficiencies in randomization, blinding, and sample-size justification, raising concerns about reproducibility. Cardiotoxicity, despite clinical relevance, was virtually absent from preclinical evaluation. **Conclusions**: Preclinical evidence suggests cytarabine’s systemic toxicity as a multifactorial process extending beyond rapidly proliferating tissues. While animal studies provide mechanistic insights, methodological weaknesses and translational gaps limit predictive value. Future research must adopt rigorous design, systematically assess underexplored toxicities, and integrate molecular profiling to identify biomarkers and protective strategies.

## 1. Introduction

Cytarabine, also known as cytosine arabinoside or 1-β-D-arabinofuranosylcytosine, is a deoxycytidine nucleoside analog that remains a fundamental pharmacologic agent in induction and consolidation therapy for patients diagnosed with acute myeloid leukemia (AML), as well as Hodgkin and non-Hodgkin lymphomas [1,2,3,4,5]. As a pyrimidine nucleoside analog, it mimics the physiological nucleoside deoxycytidine. Its primary mechanism of action involves intracellular phosphorylation to the active metabolite cytarabine triphosphate (ara-CTP), which competes with deoxycytidine triphosphate for incorporation into DNA during the S-phase of the cell cycle, inhibiting DNA polymerase and resulting in chain termination and the subsequent induction of apoptosis in rapidly proliferating malignant cells [1,2].

Despite its proven antileukemic efficacy, clinical application is constrained by a narrow therapeutic index, with significant burdens of severe, life-threatening, or fatal adverse effects that remain largely unpredictable despite decades of use [1,6,7,8,9]. This unpredictability stems from inter-individual variability in pharmacokinetics, which is modulated by genetic polymorphisms in enzymes such as cytidine deaminase (CDA). Reduced CDA activity can lead to excessive intracellular accumulation of ara-CTP, thereby precipitating heightened systemic toxicity [6,8].

The toxicity profile of cytarabine is highly dose- and regimen-dependent, posing challenges for optimizing therapeutic efficacy while minimizing harm [1]. In standard 7-day regimens (typically 100–200 mg/m^2^ daily), myelosuppression is the principal dose-limiting toxicity, manifesting as profound neutropenia, thrombocytopenia, and anemia that increase infection risks [1,10]. Gastrointestinal toxicities commonly present as mild to moderate mucositis and diarrhea, though rare but severe complications like acute pancreatitis have been reported, potentially linked to direct acinar cell damage or inflammatory cascades [11,12]. Furthermore, the “cytarabine syndrome” may occur within 12 h of infusion initiation and is characterized by fever, myalgias, arthralgias, bone pain, maculopapular rash, keratoconjunctivitis, and retrosternal pain, likely driven by cytokine release or hypersensitivity reactions [13].

The advent of high-dose cytarabine (HiDAC) regimens (2–3 g/m^2^) for consolidation therapy has further amplified the toxicity burden. HiDAC is associated with severe adverse events including biphasic pancytopenia, neurotoxicity (cerebellar ataxia, seizures or encephalopathy due to ara-CTP accumulation in cerebrospinal fluid), dermatologic rashes, alopecia and hyperbilirubinemia affecting over 10% of patients [1,10,14]. Although less frequently discussed in the context of standard chemotherapy, cardiovascular complications, such as fatal cardiomyopathy when combined with cyclophosphamide in transplant conditioning regimens, angina, pericarditis and bradycardia (incidence up to 2.8%), further underscore the drug’s multi-organ impact and may involve endothelial damage or oxidative stress [15,16].

Critically, these toxicities extend beyond immediate physiological harm, comprising patient quality of life, increased healthcare costs and necessitating treatment interruptions or dose reductions that may affect survival outcomes [6,7]. Preclinical animal models have served as an indispensable platform for elucidating the pathophysiology of these off-target effects, demonstrating reproducible patterns of multi-organ toxicity across species. However, this vast body of evidence remains fragmented, dispersed across various species, dosing schedules and areas of research, hindering cross-study comparison and translational extrapolation to safer clinical applications in AML therapy.

Therefore, the primary objective of this systematic review is to address and bridge the gap between animal and human-based studies by comprehensively synthesizing animal evidence on cytarabine’s systemic multi-organ toxicity, with the objectives to (1) catalog the spectrum of organ toxicities reported in animal models following cytarabine administration, (2) characterize dose–response relationships and treatment schedules associated with the onset, severity and temporal progression of cytarabine-induced toxicity, (3) identify the histopathological, biochemical, and functional endpoints used to quantify organ-specific damage and (4) map proposed molecular and cellular mechanisms underlying cytarabine toxicity in preclinical studies.

## 2. Materials and Methods

To guarantee methodological transparency and rigor, this systematic review was conducted in accordance with the PRISMA 2020 (Preferred Reporting Items for Systematic reviews and Meta-Analyses) guidelines [17], as detailed in Supplementary Table S1. The review was registered in PROSPERO on 15 July 2025, with ID CRD420251081384.

### 2.1. Identifying Research Questions

To clarify the research query, a PICO (population, intervention, comparator, outcome) framework [18] was employed, as detailed in Table 1. Accordingly, the following review question was developed: “In preclinical animal models (mice, rats, rabbits or drosophila), what are the systemic multi-organ toxicities induced by various dosing regimens of cytarabine (Ara-C) administration compared to control groups with no agent administered?”.

### 2.2. Identifying Relevant Studies

A comprehensive and systematic search was conducted on 15 July 2025 across multiple biomedical databases, including PubMed, MEDLINE, Scopus, Cochrane Library and Embase, using the following query strings: “(“Arabinofuranosylcytosine” OR “Arabinosylcytosine” OR “Aracytidine” OR “Cytosine Arabinoside” OR “Arabinoside, Cytosine” OR “Cytarabine Hydrochloride” OR “Cytosar” OR “beta-Ara C” OR “Aracytine” OR “Ara-C” “Cytonal” OR “Cytarabine”) AND (animal study OR animal model)”. References were reviewed for the identification of any additional relevant articles.

### 2.3. Study Selection—Eligibility and Screening

The review was restricted to full-text research studies published in peer-reviewed journals in the English language. Eligible studies had to meet the following criteria: the study must include preclinical animal models (mice, rats, rabbits or drosophila of all species and breeds); the study design must be a randomized or nonrandomized controlled study, conducted in vivo; the study should focus primarily on the systemic multi-organ toxicity of cytarabine (Ara-C) and, when available, include a control group with no agent administered. Duplicate records, review articles, systematic reviews and meta-analyses, protocols and guidelines, in vitro studies, clinical trials (all phases) on humans, conference abstracts and presentations, preprints, studies deemed irrelevant, unavailable full-text, and studies in other languages apart from English were excluded.

After the exclusion of articles based on the above criteria by both automated tools and the researchers, the final set of articles was retrieved. To ensure accuracy and objectivity, two independent reviewers (I.K. and S.T.) initially screened the titles and abstracts in a double-blinded process. For studies that passed this initial screening, the full texts were obtained and further evaluated to determine their final eligibility. Any discrepancies during the screening process were resolved by a third reviewer (T.P.).

### 2.4. Data Charting

Key data were independently extracted from all included studies by two reviewers (I.K. and S.T.), comprising the first author and year of publication, DOI, study design including experimental groups, control group and number of animals per group, animal model details such as species, sex, weight and age, intervention details including dose of cytarabine, timing of administration, frequency of administration, route of administration and vehicle, primary outcomes such as main toxicity, biomarker results and main histopathological findings of cytarabine’s toxicity and secondary outcomes including key methods used to assess cytarabine’s organ-specific toxicity.

### 2.5. Collating, Summarizing, and Reporting Results

The extracted data were synthesized into results tables. Given the systematic nature of this review, a meta-analysis was not conducted. Instead, a narrative synthesis of the principal findings was performed to explore and evaluate the systemic multi-organ toxicity of cytarabine in preclinical animal models.

### 2.6. Study Quality and Risk of Bias Assessment

The risk of bias and study quality of the included studies were assessed by two independent reviewers (I.K. and S.T.) using SYRCLE’s risk of bias tool for assessing risk of bias [19] and the CAMARADES checklist [20] and the Newcastle–Ottawa Scale (NOS) adapted for animal models [21] for study quality. Any discrepancies were resolved through discussion or by consulting a third reviewer (T.P.). Additionally, adherence to the ARRIVE 2.0 Essential 10 guidelines was evaluated, and the overall quality of evidence was assessed using the GRADE framework.

## 3. Results

### 3.1. Study Selection

**Figure 1:**
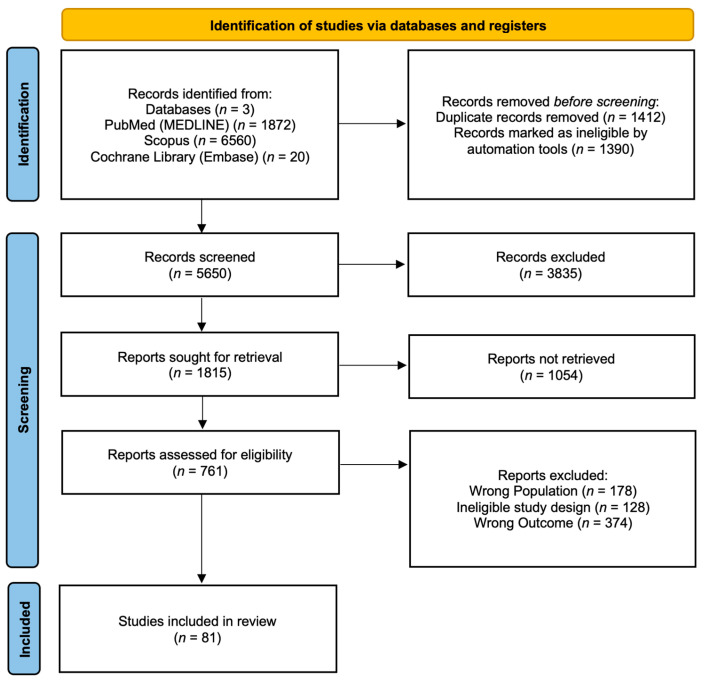
PRISMA flow diagram.

The PRISMA flow diagram (Figure 1) outlines the review selection and exclusion process. Initially, a total of 8452 records were retrieved from the aforementioned databases (PubMed and MEDLINE, *n* = 1872; Scopus, *n* = 6560; Cochrane and Embase, *n* = 20). Automated screening excluded 1390 records, leaving 7062 records for further consideration. Of these, 1412 duplicate records were removed manually. Subsequently, 3835 studies were excluded based on ineligible study design, as determined through title and abstract screening, and 1054 studies were not retrieved due to no full-text availability. After a full-text review of the remaining 761 articles, 81 studies met the inclusion criteria and were included in the review.

### 3.2. Study Characteristics

The included studies spanned dates from 1964 to 2024, exclusively focusing on in vivo animal models of cytarabine-induced toxicity. They primarily employed rodent models, with mice (*n* = 40 studies) and rats (*n* = 35 studies) representing the majority, whereas rabbits (*n* = 6) and Drosophila (*n* = 2) were used less frequently. Animal ages ranged from embryonic stages (gestation days 10–12 in teratogenicity models) to adulthood (6–8 weeks old in most murine investigations), with body weights generally between 20 and 300 g, depending on species and strain. Common strains included Wistar rats (*n* = 18), Sprague–Dawley rats (*n* = 14), C57BL/6J mice (*n* = 11), and BALB/c mice (*n* = 6), which were frequently chosen for their genetic consistency and suitability for modeling human physiological responses. Cytarabine regimens exhibited considerable variability, reflecting efforts to emulate clinical dosing in the treatment of acute myeloid leukemia. Doses ranged from 1 mg/kg to 1000 mg/kg and were administered through various routes, including intraperitoneal (i.p.; *n* = 63 studies), intravenous (i.v.; *n* = 4), subcutaneous (s.c.; *n* = 6), oral (*n* = 3), intravitreal (*n* = 1), subconjunctival (*n* = 1), ophthalmic drops (*n* = 1), intratesticular (*n* = 1), and intrathecal (*n* = 1). Treatment regimens comprised single doses, daily administrations spanning 3 to 7 days, or intermittent protocols extending over several weeks, with vehicles such as saline or phosphate-buffered saline frequently employed. Experimental groups generally consisted of cytarabine-only cohorts (*n* = 6–20 animals per group) in conjunction with saline control groups, and, in certain instances, included adjunctive interventions such as antioxidants or cytoprotectants to reduce toxicity. Group sizes ranged from 5 to 30 animals, with larger cohorts used in studies evaluating functional outcomes such as behavioral assessments.

### 3.3. Risk of Bias and Study Quality

Using the SYRCLE’s tool, there were moderate to high risks of concern in most of the studies, with only 10% (*n* = 8) rated as low overall risk (Supplementary Table S2). Sequence generation (randomization) was reported in only 30% of studies, while baseline characteristics were consistently described. Allocation concealment and random housing were unclear in nearly all cases, contributing to potential selection and performance biases. Blinding of caregivers and investigators was absent in nearly all studies, though outcome assessors were blinded in only 15%. Incomplete outcome data and selective reporting were low risks overall, with most studies addressing all planned endpoints. Other biases, such as funding conflicts, were unclear in older publications.

Study quality, as assessed per the CAMARADES 10-point checklist, indicated moderate quality overall, with a median score of 4 (range 3–8) out of 10 (maximum score) and only six studies of high quality, with strengths in peer-reviewed publication, appropriate animal models and compliance with welfare regulations (Supplementary Table S3). Weaknesses encompassed absent sample-size calculations (in all studies), absent blinded model induction (in all studies) and lack of temperature control (in 62% of studies). The adaptation of the Newcastle–Ottawa Scale for cohort-like animal studies yielded a median score of 7 (range 6–9) out of 9 (maximum score), indicating high quality among the included studies, with high representativeness of exposed cohorts and ascertainment of exposure, but variable comparability regarding confounders like age or comorbidities in 93% of studies (Supplementary Table S4). Adherence to ARRIVE 2.0 Essential 10 guidelines was moderate, with a median score of 6 (range 5–9.5) out of 10 (maximum score), with robust reporting of study design, outcome measures, experimental animals and procedures, but deficiencies in sample-size justification (absent in all studies), blinding (absent in 88%) and randomization (absent in 79%) processes (Supplementary Table S5). It is worth noting that publication bias was not detected, though the predominance of positive toxicity findings suggests potential underreporting of null results.

### 3.4. Spectrum of Organ Toxicities

The included studies documented a wide range of organ toxicities induced by cytarabine, mostly impacting rapidly proliferating tissues in accordance with the drug’s antimetabolite mechanism of action. Table 2 encapsulates the results from the studies included, Supplementary Table S6 reports the results for individual studies. Neurotoxicity was the most commonly observed toxicity, with it occurring in an estimated 30% of cases in a total of 23 studies [22,23,24,25,26,27,28,29,30,31,32,33,34,35,36,37,38,39,40,41,42,43,44], presenting as cerebellar degeneration, ataxia, and hippocampal-dependent cognitive deficits, predominantly in rat and mouse models. Intestinal toxicity, encompassing mucositis and villous atrophy, was observed in 14% of studies (*n* = 11) [45,46,47,48,49,50,51,52,53,54,55,56]. Ocular toxicity, including meibomian gland dysfunction, acinar atrophy, ductal hyperkeratinization, lacrimal gland hyposecretion, corneal epithelial erosions and retinal atrophy, was reported in ~10% of studies (*n* = 8) [35,36,57,58,59,60,61,62,63], particularly in mice exposed to high-dose i.p. regimens. Alopecia was assessed in 6 studies [64,65,66,67,68,69]. Hepatotoxicity, characterized by elevated transaminases, steatosis and fibrosis, affected ~5% of investigations (*n* = 4) [70,71,72,73]. Nephrotoxicity, characterized by tubular necrosis and glomerular damage, appeared in only 3 studies [35,36,70]. Less common injuries included myelotoxicity [74,75,76,77,78], pulmonary fibrosis [79] and reproductive toxicity, such as testicular atrophy with a detrimental impact on testicular Leydig cell number and consequently, reduced testosterone levels, following cytarabine exposure [80]. Multi-organ involvement was evident in 20% of studies, emphasizing cytarabine’s systemic impact, with no organ spared in high-dose models. Finally, 27 studies also assessed developmental toxicity following gestational exposure, along with other organ injuries, including craniofacial defects (cleft palate, encephalocele), limb and tail deformities (hemimelia, phocomelia, clubbing), digit anomalies (polydactyly, oligodactyly, ectrodactyly) and skeletal dysplasias (carpal bone fusions, absence, incomplete ossification, hypoplasia of skull, vertebral fusions) [33,34,35,36,39,41,42,43,55,61,71,80,81,82,83,84,85,86,87,88,89,90,91,92,93,94,95,96]. Notably, cardiotoxicity, which cytarabine potentially induces, has not been systematically evaluated in preclinical animal models according to the published literature.

### 3.5. Dose–Response Relationships, Treatment Schedules and Human Equivalent Dose Translation

**Figure 2:**
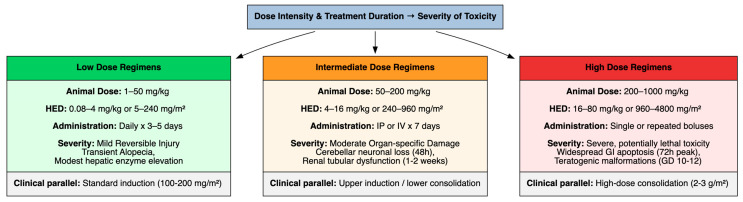
Dose-Dependent Toxicity Profile and Human Equivalent Dose Translation.

The severity of toxicity was closely linked to dose intensity and treatment duration (Figure 2). To enhance translational utility, animal doses have been converted to human equivalent doses (HEDs) using body-surface area normalization according to FDA guidance, with conversion factors of 0.08 for mouse and 0.16 for rat models [97].

Low doses, such as 1–50 mg/kg for animal models, approximately equivalent to 0.08–4 mg/kg or 5–240 mg/m^2^ in humans, often administered daily for 3–5 days, elicited mild, reversible injuries such as transient alopecia or hepatic enzyme elevations. These doses approximate conventional low-to-standard clinically used cytarabine regimens (100–200 mg/m^2^) used in induction chemotherapy for AML.

Intermediate-dose regimens, such as 50–200 mg/kg for animal models, approximately equivalent to 4–16 mg/kg or 240–960 mg/m^2^ in humans, administered i.p. or i.v. for 7 days, induced moderate organ-specific damage, including neuronal loss in the cerebellum (observed within 48 h post-administration) and renal tubular dysfunction progressing over 1–2 weeks. These doses approximate the upper range of standard induction and lower range of consolidation regimens, where neurotoxicity emerges as a dose-limiting toxicity.

High-dose regimens, such as 200–1000 mg/kg for animal models, approximately equivalent to 16–80 mg/kg or 960–4800 mg/m^2^ in humans, administered as single or repeated bolus injections, were associated with severe, potentially lethal toxicity. Widespread apoptosis in the gut mucosa, peaking at 72 h, and teratogenic malformations when administered during critical gestational windows, such as gestational days 10–12 in rats, corresponding to early organogenesis, were induced. These high-dose regimens are consistent with intensive consolidation protocols (2–3 g/m^2^ per dose) employed in AML therapy, where neurotoxicity, gastrointestinal injury and ocular complications become clinically prominent.

**Figure 3:**
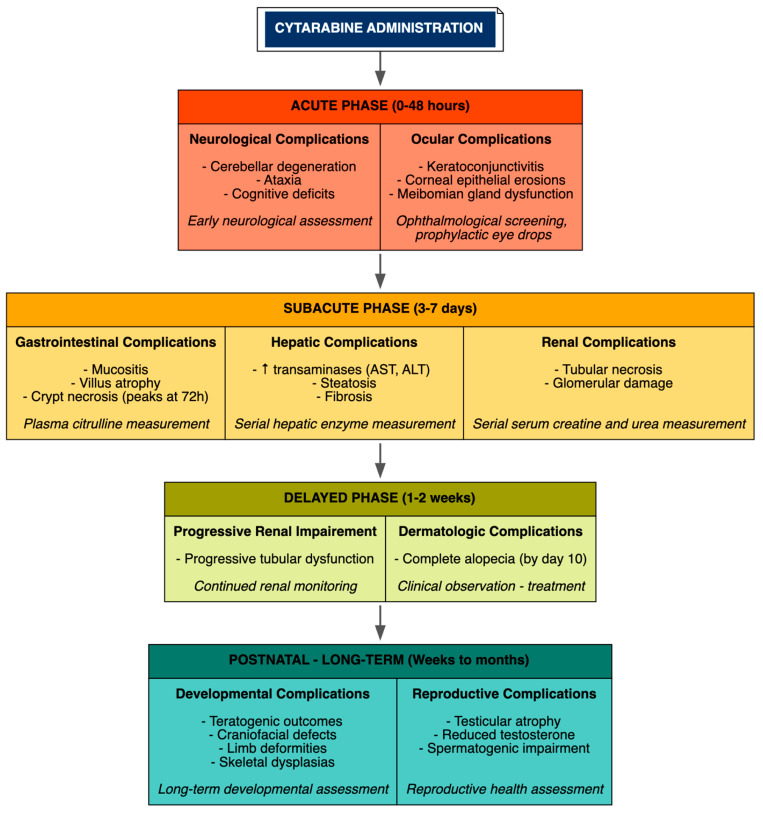
Temporal Progression of Organ-Specific Toxicities.

Temporal progression varied by organ, reflecting distinct vulnerabilities of proliferating tissues and organ-specific metabolic demands. Ocular and neurological effects appeared acutely within 24–48 h post-administration, with neuronal loss in the cerebellum observed within 48 h and corneal epithelial erosions and keratoconjunctivitis manifesting early in high-dose models, necessitating early neurological and ophthalmological assessment. Hepatic and renal injuries developed subacutely over 3–7 days, with progressive renal tubular dysfunction extending over 1–2 weeks, emphasizing the need for serial measurement of hepatic transaminases and renal function markers starting from day 3. Gastrointestinal toxicity, characterized by widespread apoptosis in the gut mucosa, peaked at 72 h, with catastrophic villus and crypt destruction observed by day 3 in high-dose exposures. Dermatologic toxicity followed a delayed course, with complete alopecia typically evident by day 10 following treatment initiation. Teratogenic outcomes manifested postnatally when cytarabine was administered during critical gestational windows, such as gestational days 10–12 in rats corresponding to early organogenesis. Routes of administration influenced severity, with intrathecal administration exacerbating neurotoxicity, while treatment schedules mimicked clinical consolidation, such as 100 mg/kg daily for 5 consecutive days, reproducibly induced multi-organ patterns across preclinical models (Figure 3).

### 3.6. Histopathological, Biochemical and Functional Endpoints

Across studies, a wide range of histopathological, biochemical, and functional endpoints were employed. Histological analyses revealed acinar dropout in meibomian glands, villus shortening and crypt necrosis in the intestine, dendritic retraction in cortical neurons and mitochondrial disruption in Drosophila midgut epithelia. Biochemical assays consistently suggested oxidative stress, with increased lipid peroxidation markers (MDA, 4-HNE, 8-OHdG) and reduced antioxidant enzyme activity (SOD, CAT, GSH-Px). Functional outcomes included impaired tear production, reduced locomotor activity, cognitive deficits in Morris water maze testing and decreased fertility indices.

### 3.7. Molecular and Cellular Mechanisms

Multiple, interrelated mechanistic pathways were consistently implicated across organ systems, highlighting that cytarabine toxicity cannot be reduced to a single mode of injury. Oxidative stress and the collapse of endogenous antioxidant defenses emerged as central drivers, particularly in ocular, intestinal, hepatic and developmental models. Studies consistently documented accumulation of lipid peroxidation products and depletion of glutathione and enzymatic scavengers that temporally preceded structural injury. Inflammatory cascades, typified by TNF-α and IL-6 upregulation and skewed macrophage polarization toward pro-inflammatory M1 phenotypes, were robustly documented in intestinal injury models and correlated with amplified mucosal damage. Interventional studies showing that macrophage depletion or anti-inflammatory agents attenuate injury provide strong preliminary evidence for a causal role of inflammation. Apoptotic signaling, evidenced by caspase activation, p53 induction and DNA fragmentation, was a ubiquitous feature across intestinal, developmental and neural tissues.

**Figure 4:**
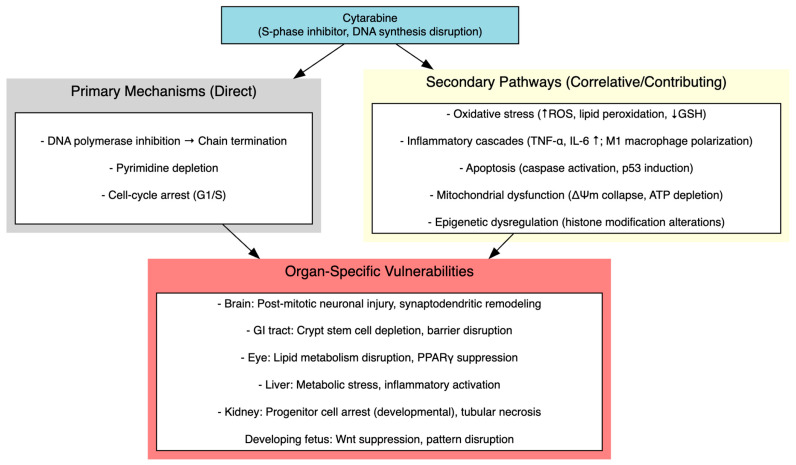
Molecular and Cellular Mechanisms of Cytarabine-Induced Multi-Organ Toxicity.

In the nervous system, toxicity extended beyond antimetabolite-mediated mitotic arrest of progenitors to encompass dendritic atrophy, selective loss of mature spines, mitochondrial dysfunction, neurofilament degradation and epigenetic dysregulation, collectively disrupting synaptic plasticity and circuit integrity rather than producing uniform neuronal loss. Ocular injury involved dysregulated lipid metabolism, including loss of PPARγ nuclear localization, impaired lipogenic enzyme expression (AWAT2, SOAT1, ELOVL4), compensatory upregulation of cholesterologenesis and suppression of the AKT/FoxO survival signaling axis. These findings suggest that metabolic disruption, particularly in lipid-rich tissues such as meibomian glands, may represent a tissue-specific vulnerability. Oxidative disequilibrium, with accumulation of lipid peroxidation markers and blunted Nrf2/HO-1 antioxidant defenses, likely compounds these metabolic deficits. Developmental anomalies reflected a convergence of proliferation arrest, Wnt suppression, p53-dependent apoptosis and premature gliogenic shifts, consistent with disrupted embryonic patterning. Finally, in Drosophila, activation of JAK-STAT, JNK and Toll/IMD pathways suggested that cytarabine engages conserved innate immune and apoptotic programs, reinforcing the view that its systemic toxicity arises from a nexus of oxidative, inflammatory, apoptotic and epigenetic disturbances rather than a single linear pathway. Figure 4 summarizes the proposed molecular and cellular mechanisms of cytarabine- induced multi-organ toxicity.

## 4. Discussion

This systematic review of preclinical animal research provides preliminary evidence for cytarabine’s S-phase-specific antimetabolite activity that can lead to dose- and regimen-dependent systemic multi-organ toxicities, mostly in rapidly proliferating tissues. Neurotoxicity, gastrointestinal mucositis, and ophthalmic impairment were the most common side effects, but hepatic, renal, reproductive, and developmental toxicities suggest cytarabine’s systemic influence. These findings support clinical observations of chemotherapy-related complications in acute myeloid leukemia patients and provide translational insights into underlying mechanisms like oxidative stress, inflammation, apoptosis, and cell-cycle disruption, enabling organ-specific protective interventions and dosing optimization to improve therapeutic indices in humans.

### 4.1. Cytarabine-Induced Neurotoxicity

Neurotoxicity was identified as the predominant adverse effect of cytarabine in 23 preclinical studies, corresponding to clinical observations where it represents a significant dose-limiting toxicity, impacting up to 12% of patients undergoing high-dose intravenous treatments and as many as 55% of those with renal impairment [1,98]. It presented as cerebellar degeneration, disruption of hippocampal circuits, cognitive and behavioral deficits and developmental anomalies, predominantly in juvenile rodents and, in one instance, a Drosophila model, with cumulative doses varying from 15 to 2800 mg/kg delivered via intraperitoneal, intrathecal, or oral routes.

Cerebellar pathology was characterized by the loss of Purkinje cells, disorganization of the monolayer, depletion of granule cells, and cytotoxicity, alongside molecular changes such as hyporeactivity of axonal neurofilaments (NFs), selective degradation of the NF-H isoform (approximately 40% reduction), dysregulation of calbindin D-28K, oxidative stress (elevated MDA, reduced GSH), DNA strand breaks, apoptosis (TUNEL+, p53+, caspase-3+), and epigenetic modifications (increased H3 acetylation and K4 methylation, decreased H3K9 methylation) [27,28,29]. The lesions were associated with motor ataxia, as suggested by an expanded footprint base, increased step width, deficits in rotarod latency, and ambulatory hypokinesia [29], mirroring the clinically observed acute cerebellar syndrome, which is characterized by dysarthria, nystagmus, ataxia, and, in severe instances, irreversible disability, typically occurring 3–8 days following the initiation of high-dose treatment [1,99].

Hippocampal vulnerability was marked by dendritic atrophy, arbor simplification and selective loss of mature mushroom spines (~50% reduction in DG, CA1 and CA3), without overt necrosis or cell loss, but accompanied by leukocytosis suggestive of neuroinflammation. These alterations likely underline the cognitive deficits, memory impairments and encephalopathy reported in long-term survivors, particularly pediatric patients [100]. Juvenile intrathecal administration of Ara-C plus methotrexate impaired Morris Water Maze (MWM) probe trial performance, reducing quadrant preference and platform crossings while sparing acquisition and swim speed [22]. In the anterior cingulate cortex (ACC), layer II/III pyramidal neurons exhibited apical dendritic retraction (~15% reduced length, ~36% fewer branch points, ~51% reduced spine density), impairing remote but not recent memory, consistent with delayed circuit remodeling [24] and potentially explaining the delayed neurocognitive sequelae observed in leukemia survivors [100]. By contrast, some studies reported no MWM deficits despite ~9% weight loss [23].

Supratentorial pathology included neuronal pyknosis, cytoplasmic depletion, nuclear vacuolization and granule layer fragmentation across multiple regions, mediated by monoamine depletion, cholinesterase inhibition, oxidative stress (increased TBARS, MDA and H_2_O_2_; decreased GSH) and mitochondrial dysfunction (decreased succinate dehydrogenase, increased ROS, swelling and ΔΨm collapse) [25,26,31,37]. Such widespread injury plausibly accounts for clinical manifestations including seizures, somnolence, confusion and, rarely, fatal outcomes, with heightened risk in elderly patients or those with hepatic or renal dysfunction [1,98].

Developmental neurotoxicity encompassed neural tube defects (exencephaly ~30% at GD7.5), neuroepithelial disorganization, proliferation arrest (~65% reduction in PH3^+^ cells), increased apoptosis (2–3-fold rise in cleaved caspase-3), gliogenic shift (reduced Nestin, increased GFAP) and Wnt suppression (~50–65% reduction in β-catenin), recapitulating embryonic stem cell G_1_ arrest and differentiation skew [30]. Neonatal exposure induced cerebellar hypoplasia, Purkinje degeneration, heterotopic gray matter, microcephaly, hydrocephalus and impaired brain growth [32,34,35,36,40,41,42,44], alongside p53-dependent fetal lesions [101], sensorimotor gating deficits [43] and neurochemical disturbances [39]. In a rabbit herpes simplex encephalitis model, Ara-C evoked pathological brain changes [38]. In Drosophila, chronic exposure produced dose-dependent locomotor impairment, reduced lifespan and upregulation of the ROS, JNK, and JAK-STAT pathways [55].

**Figure 5:**
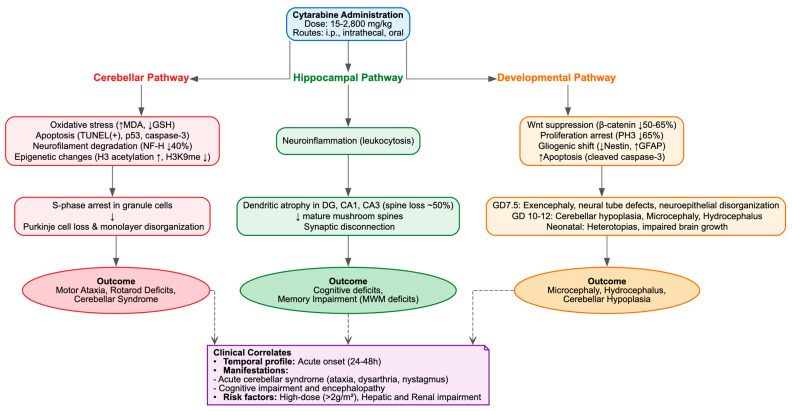
Cytarabine-Induced Neurotoxicity.

Concluding, these findings emphasize that Ara-C neurotoxicity is not limited to antimetabolite-mediated mitotic arrest in progenitors, but also extends to oxidative and mitochondrial injury, epigenetic disruption, apoptosis and protracted synaptodendritic remodeling in post-mitotic neurons (Table 3, Figure 5). This pattern is region-specific (cerebellum > hippocampus > supratentorial) and translates into enduring behavioral and cognitive consequences.

### 4.2. Cytarabine-Induced Gastrointestinal Toxicity

Cytarabine induced marked dose- and regimen-dependent gastrointestinal injury in 11 preclinical studies, predominantly targeting the small intestine (duodenum, jejunum, ileum) and, to a lesser extent, the colon. The dominant pathological picture was mucositis, characterized by villus atrophy (shortening, blunting, flattening), crypt hypoplasia or necrosis, enterocyte vacuolization and sloughing, dense lamina propria and submucosal inflammatory infiltrates, muscularis edema, cryptal abscesses and, in some cases, colonic polyps or xanthomatized enterocytes [46,47,49,51,52]. These findings mirror clinical observations in AML patients, where cytarabine frequently causes dose-dependent mucositis and diarrhea, often accompanied by abdominal pain and, in severe cases, necrotizing enterocolitis [1,102]. Additional complications such as ileus, hematemesis, melena, and vascular lesions including telangiectasia or intramural hematomas have also been reported [103].

The ileum appeared particularly vulnerable, showing flattened villi, crypt epithelial necrosis, vacuolated enterocytes, inflammatory infiltrates and tight-junction disruption with reduced ZO-1/occludin expression [46]. In contrast, jejunal crypts showed ~50% reduction in mitotic index without overt necrosis, suggesting impaired regenerative capacity [45,52]. In germ-free and conventional mice, short-chain fatty acids partially mitigated villus blunting, necrosis and lamina propria inflammation, though these lesions worsened under elemental diets [47]. Ultrastructural studies in Drosophila revealed epithelial edema, apical nuclear displacement, sparse microvilli and mitochondrial membrane rupture with matrix rarefaction, producing shortened and thinned guts but preserved permeability [55]. Notably, the study by Park M-R et al. (2023) reported preserved villus and crypt architecture with intact tight junctions, yet profound functional impairment was evident, including remodeling of lacteal lymphatic endothelium from permeable “button-like” to impermeable “zipper-like” VE-cadherin junctions, VEGFR2/AKT hyperphosphorylation, sequestration of oversized chylomicrons (>600 nm) in trans-Golgi cisternae, impaired lipid absorption, delayed intestinal transit, and cachexia independent of anorexia [104]. This functional malabsorption correlates with clinical reports of cytarabine-induced diarrhea, weight loss and metabolic derangements, particularly under high-dose regimens where complications such as pancreatitis, electrolyte disturbances, and protein-losing enteropathy have been documented [1,103].

The study by Minden MD et al. (2024) showed the lethality of high-dose exposure, with 100% mortality by day 3 following 300 mg/kg Ara-C, accompanied by catastrophic villus and crypt destruction, inflammatory infiltrates, reduced plasma citrulline (a surrogate for enterocyte mass) and dysbiosis marked by decreased Firmicutes and increased Bacteroidetes species [51]. Notably, reduced citrulline is already recognized clinically as a biomarker of enterocyte loss and a predictor of mucositis severity [105].

At the mechanistic level, cytarabine-induced gastrointestinal toxicity converged on dysregulated inflammation, oxidative stress, and impaired repair responses. Key features included pro-inflammatory M1 macrophage polarization (increased CD86^+^, iNOS^+^, CD11b^+^F4/80^+^ with reduced CD206^+^ M2), upregulation of TNF-α and IL-6 with IL-10 suppression via AKT/PI3K [46] and JAK2/STAT1 signaling [49], increased ROS production with reduced antioxidant defenses (SOD, CAT), activation of innate immune pathways (Toll/IMD, JAK-STAT, JNK) and apoptosis [55].

**Figure 6:**
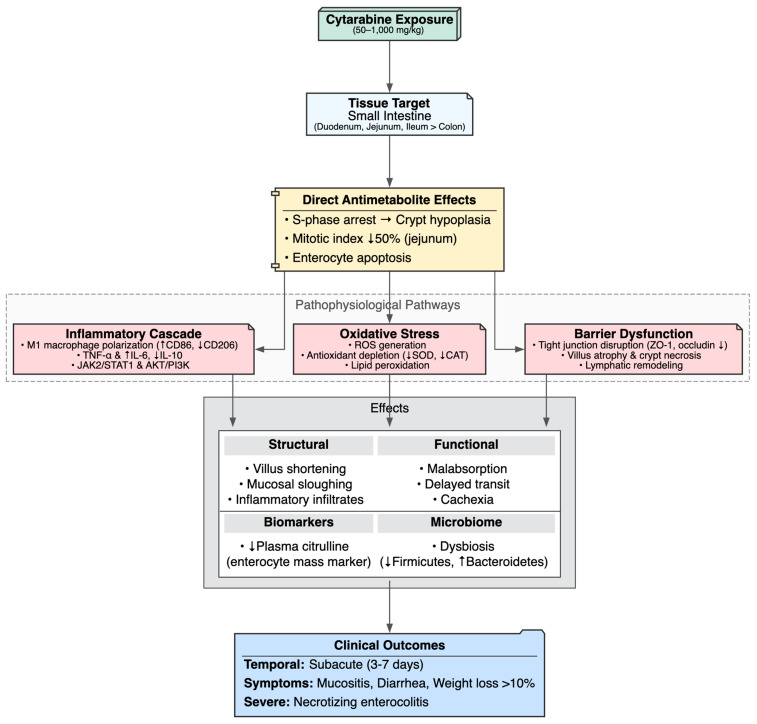
Cytarabine-Induced Gastrointestinal Toxicity.

Together, these processes culminated in epithelial barrier breakdown, regenerative failure, nutrient malabsorption, systemic inflammation, progressive weight loss exceeding 10% and, ultimately, mortality. The evidence highlights cytarabine’s profound disruption of intestinal homeostasis in rapidly renewing mucosa, raising critical translational questions about how such injury can be predicted, mitigated, or reversed in clinical practice (Table 4, Figure 6).

### 4.3. Cytarabine-Induced Ocular Toxicity

Ocular toxicity, observed predominantly under high-dose intraperitoneal or locally administered regimens, was reported across eight preclinical investigations in murine, rabbit and rat models. The study by Liu et al. (2024) showed that systemic cytarabine (50 mg/kg i.p. daily for 7 days) induces meibomian gland dysfunction and ocular surface injury in C57BL/6J mice with punctate corneal epithelial erosions (elevated corneal fluorescein staining), a ~50% reduction in tear volume due to lacrimal myoepithelial degeneration (reduced α-SMA and AQP5) and meibomian gland anomalies including orifice plugging and a 40% loss of acinar area [57]. Histopathology revealed acinar atrophy, ductal dilation with lipid stasis, full-thickness ductal hyperkeratinization (K1^+^/K10^+^) and depletion of progenitor basal cells (reduced PCNA^+^, p63^+^, Lrig1^+^) [57].

The study by Balci YI et al. (2017) corroborated these findings in Wistar rats (400 mg/kg i.p. for 5 days), reporting keratoconjunctivitis with a 19-fold rise in total oxidant status and a 9-fold increase in oxidative stress index [58]. These results implicate reactive oxygen species as central mediators of ocular surface injury, compounding cytarabine’s antimitotic effects.

Other studies highlighted route-dependent differences. The study by Rootman et al. (1983) compared subconjunctival and intravenous administration (37.5 mg/kg) in New Zealand white rabbits, where subconjunctival injection induced transient conjunctival inflammatory infiltrates and focal superficial erosions resolving within one week, while deeper ocular structures remained unaffected [59]. The study by Diets-Ouwehand et al. (1992) examined intravitreal cytarabine (600–2700 μg) in chinchilla rabbits, where doses ≥1500 μg caused transient blood–retina barrier disruption and irreversible b-wave attenuation on electroretinography, consistent with photoreceptor or bipolar cell dysfunction [60]. Although light microscopy showed no overt pathology, electron microscopy revealed synaptic pedicle abnormalities in photoreceptors [60]. The study by Percy and Danylchuk (1977) described severe retinal dysplasia in postnatal rats with rosette formations, photoreceptor and bipolar cell disarray, nuclear displacement, degeneration and retinal thinning, attributed to mitotic arrest during gliogenesis and neurogenesis [61]. Similarly, the study by Shimada et al. (1973) reported retinal rosettes in mice, suggesting cytarabine’s disruption of retinal progenitor proliferation [62]. The study by Kaufman et al. (1964) documented corneal epithelial toxicity in rabbits, characterized by edema, opacity and sloughing [63]. Developmental ocular defects were also evident in the research by Percy and Albert (1974), who suggested postnatal proliferative blockade in rodents [35], while the study by Percy (1975) proposed teratogenic retinal anomalies in late fetal models [36].

At the mechanistic level, cytarabine-induced ocular toxicity appears to converge on a cascade of disrupted lipid homeostasis, impaired survival signaling, and unchecked oxidative stress. Loss of PPARγ nuclear localization undermines lipogenic programs (AWAT2, SOAT1, ELOVL4), while simultaneously driving a compensatory but maladaptive upregulation of cholesterologenesis (increased HMGCR and cholesterol accumulation) [57]. This metabolic imbalance is compounded by suppression of the AKT/FoxO axis: reduced phosphorylation of AKT and FoxO1/3a permits their nuclear retention, shifting transcriptional control toward pro-apoptotic and stress-responsive pathways [57]. In parallel, oxidative disequilibrium becomes entrenched, with accumulation of lipid peroxidation and DNA damage markers (4-HNE, 8-OHdG), alongside increased Keap1 and blunted Nrf2/HO-1/SOD1 antioxidant defenses [57].

**Figure 7:**
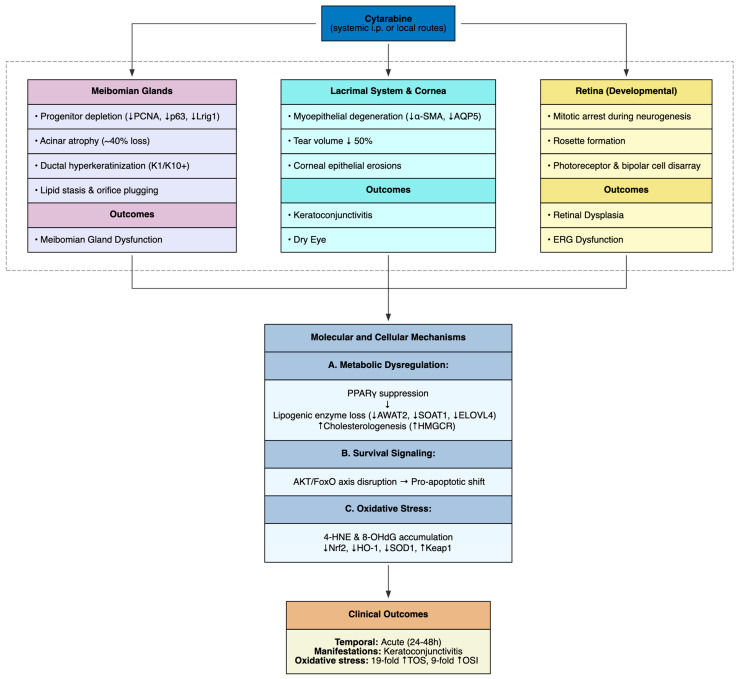
Cytarabine-Induced Ocular Toxicity.

Taken together, these studies reveal cytarabine’s consistent targeting of proliferative ocular compartments through S-phase inhibition, oxidative stress, and developmental interference, culminating in epithelial attrition, glandular atrophy, dyskeratosis, and neuroretinal maldevelopment (Table 5, Figure 7). These preclinical findings mirror clinical experience, where high-dose cytarabine frequently induces keratoconjunctivitis, manifesting as conjunctivitis or keratitis in a substantial proportion of patients [106,107].

### 4.4. Cytarabine-Induced Alopecia

Several studies have investigated cytarabine-induced alopecia in rodent models, highlighting its macroscopic manifestation as complete body hair loss. The study by Jimenez et al. (1992) showed that intraperitoneal Ara-C at 20 mg/kg daily for 7 days in 7-day-old Fisher rats with C51 chloroleukemia resulted in complete alopecia by day 10, whereas co-administration of recombinant human interleukin-1β not only prevented hair loss but also enhanced leukemia suppression, plausibly through IL-1–mediated cell-cycle arrest of follicular matrix cells [64]. In a parallel study by Jimenez et al. (1992) reported that combined cyclophosphamide (50 mg/kg) and Ara-C (50 mg/kg) for 4–5 days induced total alopecia in all Sprague–Dawley rats, while topical or subcutaneous liposomal ImuVert plus N-acetylcysteine conferred 70–100% protection, likely by attenuating oxidative stress and modulating immune responses within follicles [65]. Extending these findings, Jimenez et al. (1992) suggested that IL-1 protected follicles both in vivo and in vitro, suggesting that cytokine-induced quiescence of proliferating matrix cells may allow them to evade Ara-C’s antimitotic effects [66].

The study by Sun et al. (2009) further suggested that Ara-C induced alopecia in rodents, with severity reduced by active hexose correlated compound (AHCC), an immunostimulatory extract thought to act through antioxidant and anti-inflammatory pathways [67]. The study by Hussein et al. (1995) reported that topical minoxidil completely prevented alopecia in Ara-C-treated rats, most likely by enhancing follicular perfusion and counteracting Ara-C-induced anagen disruption [68]. Clinically, minoxidil has also been trialed in chemotherapy-induced alopecia, with low-dose oral formulations accelerating regrowth in some patients [108,109]. Finally, the study by Hagiwara et al. (2011) observed that Ara-C (20 mg/kg i.p. daily for 7 days) induced complete alopecia in 8-day-old Wistar rats, accompanied by dense polymorphonuclear and lymphocytic infiltration of hair follicles, suggesting an inflammatory component, while electron microscopy (EM) showed swollen mitochondria, disrupted cristae and flocculent matrix in hair-matrix cells [69].

**Figure 8:**
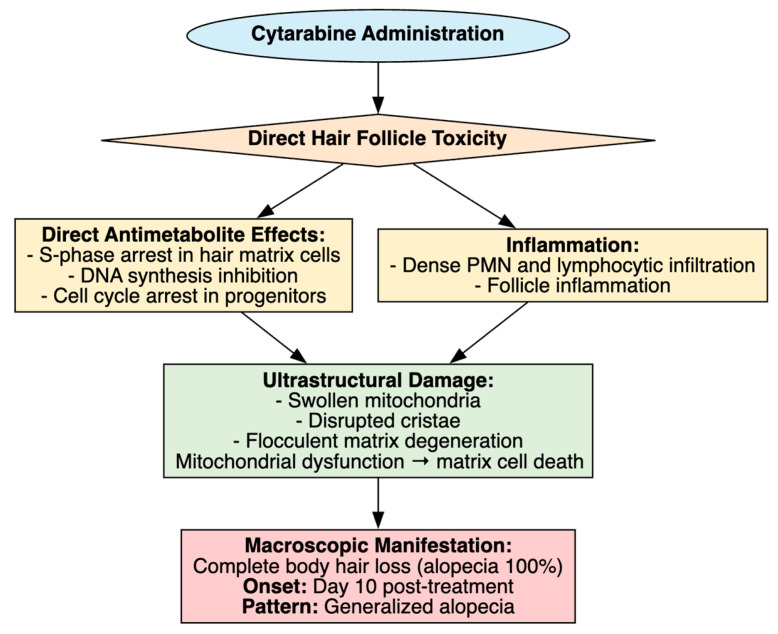
Cytarabine-Induced Alopecia.

Collectively, these studies highlight Ara-C’s direct cytotoxicity to rapidly dividing hair matrix cells, with alopecia serving as a visible marker of its broader antimetabolite effects (Table 6, Figure 8). Clinically, alopecia is most pronounced at higher Ara-C doses, where complete hair loss is more frequent than with standard regimens [110] and it is often distressing, with over 50% of affected patients considering it the worst aspect of chemotherapy [111]. Advances in formulation, such as liposomal CPX-351, which has shown markedly lower alopecia rates (11%) compared with conventional cytarabine plus daunorubicin, suggest that drug delivery innovations may offer the most realistic path toward mitigating this psychologically burdensome toxicity [112].

### 4.5. Cytarabine-Induced Hepatotoxicity

Cytarabine-induced hepatotoxicity has been consistently suggested in preclinical models, revealing patterns of oxidative stress, inflammatory activation and structural injury. In BALB/c nude mice given i.p. cytarabine at 2.5 mg/kg daily for up to 20 days, the study by Sun et al. (2019) reported progressive liver atrophy with concomitant weight loss, mechanistically linked to G1/S cell-cycle arrest via upregulation of the INK4 family genes (CDKN2A–D) and inhibition of the CDK4/cyclin D1 complex [70]. Histopathology showed growth-restrictive architectural disruption [70]. The study by Kolure et al. (2023) extended these findings in pregnant Sprague–Dawley rats exposed orally to 25 mg/kg daily from gestational days 8–20, documenting maternal hepatotoxicity with marked elevations in AST, ALT, urea, and creatinine, increased hepatic MDA, and suppression of antioxidant defenses (CAT, SOD, GSH, GSH-Px) [71]. H&E sections revealed lobular disorganization, vacuolization, pycnotic nuclei, and sinusoidal dilation. In rabbits treated intraperitoneally with 50 mg/kg daily for 7 days, the study by Al-Jammas et al. (2020) observed mononuclear and Kupffer cell infiltrates, portal fibrosis, bile-duct hyperplasia, vascular congestion, coagulative necrosis and sinusoidal distension [72]. Finally, the study by Dudina et al. (2018) reported acute cytotoxic liver injury in Wistar rats administered intravenous cytarabine at 2 g/m^2^ daily for 5 days, with elevated AST, ALT, GGTP and ALP, central-lobular necrosis, karyolysis, vacuolar degeneration, steatosis, portal fibrosis and bile-duct hyperplasia [73]. Mechanistically, ELISA and immunohistochemistry revealed increased TNF-α and HGF, reduced IL-10, upregulated Bcl-2 and diminished Ki-67, indicating a dysregulated balance of inflammation, apoptosis and regeneration [73].

**Figure 9:**
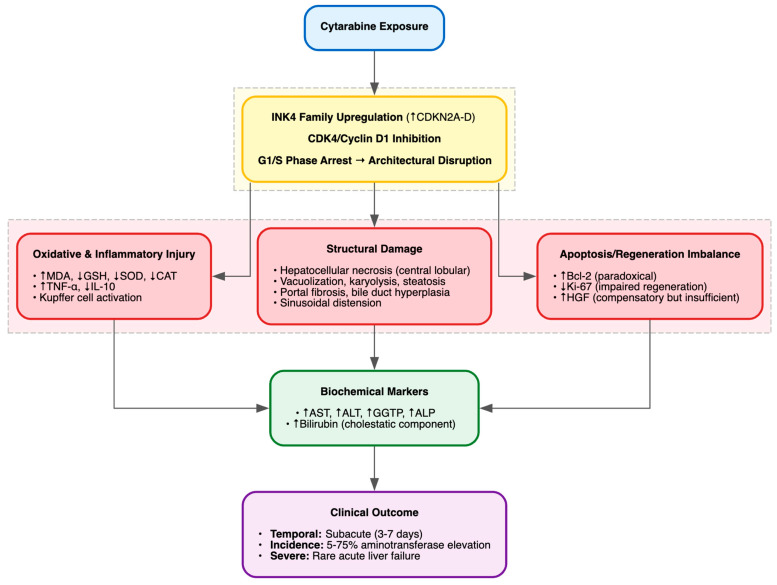
Cytarabine-Induced Hepatotoxicity.

Summarizing, these studies underscore a hepatotoxic profile driven by cell-cycle arrest, oxidative imbalance, and pro-inflammatory signaling, yet the evidence base remains narrow and methodologically limited (Table 7, Figure 9). Clinically, cytarabine, especially at high doses, has been associated with hepatotoxicity ranging from transient aminotransferase elevations in 5–75% of patients to rare but severe cases of acute liver failure [113]. Cases reports describe acute hepatic failure with elevated transaminases, mild hyperbilirubinemia and preserved ALP [114], as well as isolated direct hyperbilirubinemia suggestive of cholestatic injury [115]. These clinical observations echo preclinical findings of hepatocellular necrosis, cholestasis and inflammatory injury, underscoring the translational relevance but also exposing the lack of systematic evaluation.

### 4.6. Cytarabine-Induced Renal Toxicity

In preclinical models, cytarabine has been shown to induce nephrotoxicity primarily through disruption of DNA synthesis and inhibition of proliferative capacity, as shown in three key studies. The study by Percy et al. (1974) administered cytarabine at 3.125–50 mg/kg daily for five consecutive days beginning on postnatal day 1, 5 or 10 in ICR Swiss albino mice and Sprague–Dawley rats [35]. They reported developmental defects, including kidney lesions characterized by focal cortical dysplasia, subcapsular nests of undifferentiated primordial renal cells and arrested glomerular and tubular development, with severity greatest when dosing began on postnatal day 1 at doses ≥25 mg/kg/day. In a subsequent study, the study by Percy (1975) examined late fetal exposure in ICR Swiss albino mice (gestational days 16–18) and Sprague–Dawley rats (gestational days 18–20) using subcutaneous injections of 12.5–50 mg/kg/day for three days [36]. Cytarabine produced dose-dependent renal malformations, most pronounced at 50 mg/kg/day, including focal subcapsular microcysts composed of dilated tubules lined by vacuolated epithelium and nests of primordial cells, consistent with arrested nephrogenesis [36]. Mechanistically, these effects reflected impaired pyrimidine incorporation into DNA, leading to defective progenitor cell division and disrupted organogenesis [36]. More recently, the study by Sun et al. (2019) showed cytarabine-induced renal atrophy in 5–8-week-old BALB/c nude mice treated intraperitoneally with 2.5 mg/kg daily for up to 20 days [70]. Findings showed dose-dependent body-weight loss, gross kidney atrophy and histological evidence of inflammation and architectural disruption, mediated by upregulation of the INK4 family genes (CDKN2A–D) and inhibition of the CDK4/cyclin D1 complex, resulting in G1/S cell cycle arrest and impaired tissue growth [70].

**Figure 10:**
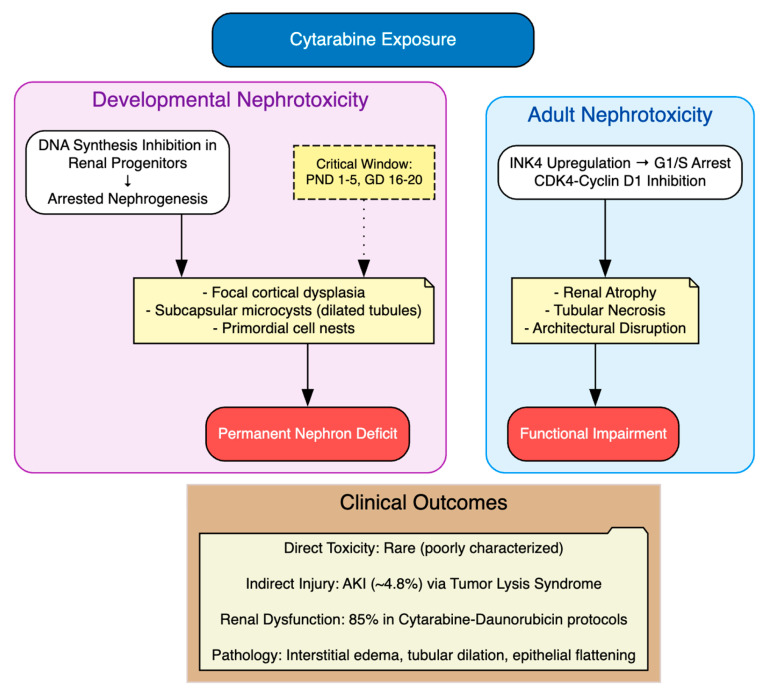
Cytarabine-Induced Nephrotoxicity.

To sum up, these studies establish cytarabine’s nephrotoxic potential via proliferative inhibition, with consequences for both developmental and adult renal integrity. Yet, translation to the clinical setting remains problematic: direct nephrotoxicity in humans is poorly characterized and often inferred indirectly (Table 8, Figure 10). Cytarabine has been associated with acute kidney injury (AKI) in approximately 4.8% of patients, most often secondary to tumor lysis syndrome (TLS) with urate and calcium phosphate deposition in renal tubules [116]. Clinical regimens incorporating cytarabine have also been linked to high rates of renal impairment. For example, 85% of patients receiving a cytarabine–daunorubicin protocol developed renal dysfunction, with pathology showing interstitial edema, tubular dilatation, epithelial flattening, focal atypia and mitotic figures in tubular epithelium [103]. Rare but notable cases of direct renal dysfunction have been described, such as a 49-year-old woman with myelodysplastic syndrome who developed transient acute renal failure and hepatic dysfunction during low-dose cytarabine therapy, possibly reflecting an allergic mechanism [117].

### 4.7. Cytarabine-Induced Developmental Toxicity

The developmental toxicity of cytarabine is well-established across rodent and non-mammalian models, manifesting as fetal growth restriction, congenital malformations, skeletal anomalies and neurodevelopmental impairments. These outcomes are consistently attributed to its antimetabolite activity, which disrupts DNA synthesis and triggers apoptosis.

In prenatal exposure studies, Ara-C produced clear, dose-dependent fetotoxicity, including resorptions, reduced fetal weight and length and external malformations such as phocomelia, oligodactyly, brachydactyly and hematomas. Skeletal ossification was markedly delayed, with diminished ossification centers, reduced bone mineral content and histopathological evidence of impaired ossification in the skull, vertebrae, sternum and limbs [36,80,82]. Limb malformations, particularly polydactyly and syndactyly, were notable, with histopathology revealing extra metacarpals and phalanges. Mechanistically, these defects were linked to expanded FGF4 expression in limb buds and disrupted Wnt/β-catenin signaling, driving premature differentiation and reduced neuroepithelial proliferation [30,81,84,85,86,87,88,89,90,91,92,93,94,95,96].

Neurodevelopmental effects included neural tube defects like exencephaly with disorganized neuroepithelium, cerebellar hypoplasia with Purkinje and granule cell loss, misalignment and heterotopic granule cells, as well as hydrocephalus and microcephaly with gray matter heterotopia. These were mediated by p53-dependent apoptosis, cell-cycle arrest, oxidative stress, and cytoskeletal disruption, highlighting the vulnerability of the developing nervous system to Ara-C [32,33,34,35,38,39,40,41,42,43,44,83]. In Drosophila, Ara-C induced developmental delay, reduced pupal size and eclosion rates and midgut epithelial injury characterized by edema, truncated microvilli, and mitochondrial rupture. These effects were mediated by oxidative stress, apoptosis and upregulation of the JAK-STAT and JNK signaling pathways [55].

**Figure 11:**
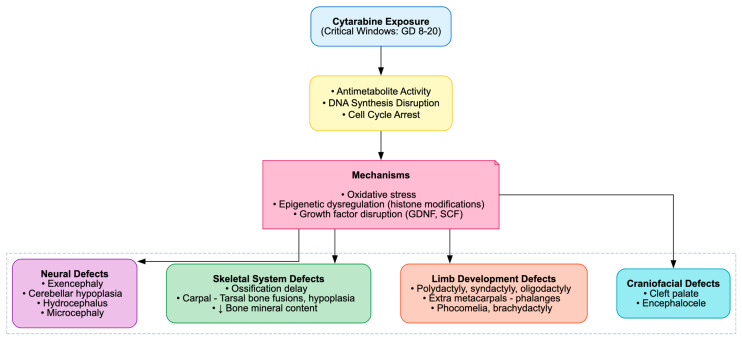
Cytarabine-Induced Developmental Toxicity.

Postnatal and juvenile exposures further exacerbated cerebellar neuronal injury, dendritic retraction and long-term spermatogenic impairment, with seminiferous tubule distortion, vacuolization, and reduced germ-cell layers. These outcomes were associated with oxidative stress, histone modifications, and dysregulation of growth factors such as GDNF and SCF (Table 9, Figure 11) [22,23,24,25,26,27,28,29,31].

### 4.8. Other Cytarabine-Induced Toxicities

Pulmonary toxicity, though rarely investigated, was exemplified by Bilgin et al. [79] (2020). They reported non-cardiogenic diffuse alveolar edema and hemorrhage, and inflammatory infiltration in rats, with increased oxidative stress markers (MDA and GSH depletion) and NF-κB activation with TNF-α upregulation, after i.p. cytarabine 200 mg/kg for 14 days. These findings raise the possibility that cytarabine may contribute to respiratory compromise in susceptible patients.

Salivary gland toxicity, while less immediately life-threatening, illuminates another troubling blind spot. Saif A-J et al. (2024) [50] documented parotid gland acinar necrosis, ductal degeneration and stromal thickening and fibrosis in rabbits receiving 60 mg/kg i.p. for 10 days, with TNF-α elevation and Bcl-2 modulation indicating apoptotic injury. This mirrors clinical observations of xerostomia and sialadenitis that impair quality of life [1].

Reproductive toxicity remains strikingly underexplored. The study by Khaleel et al. (2022) [118] addressed the critical question of pediatric exposure and reported that juvenile exposure led to irreversible azoospermia, persistent testicular atrophy and a complete loss of spermatogonia that stemmed from dysregulation of GDNF, SCF and IL-10. The study by Orth et al. (1988) [119] focused primarily on the cytarabine-induced reduction in the Sertoli cells, confirmed by the reduced level of Androgen-Binding Protein, that can lead to permanent oligospermia after exposure during the perinatal or neonatal period. Moreover, Watanabe et al. (1992) [120] observed that cytarabine causes significant atrophy and weight loss in the prostate and seminal vesicles, possibly by disrupting androgen signaling or protein synthesis. Also, they identified a specific metabolic signature, an increased ratio of Putrescine to Spermidine, which suggests that cytarabine’s metabolic toxicity extends to the biochemical pathways supporting glandular secretion. Finally, Palo AK et al. (2009) [74] showed clastogenicity and a dose-dependent increase in chromosomal aberrations and micronuclei in both bone marrow and spermatogonial cells, raising concerns about genotoxic transmission through the male germline. These findings question the long-term reproductive safety of cytarabine, particularly in younger populations, and underscore the necessity of genetic counseling and potentially delaying conception for survivors of chemotherapy to allow for the clearance of damaged germline cohorts.

Hematopoietic suppression, consistently observed in studies confirms myelotoxicity as a central, dose-limiting adverse effect, yet the mechanistic nuances remain superficially studied. Lee JY et al. (2018) [75] investigated the integrity of the vascular niche in mice treated with 100 mg/kg cytarabine and they observed a structural collapse of the sinusoids, leading to a fragmented and leaky vascular network, and a concurrent depletion of megakaryocytes, likely secondary to the loss of their vascular scaffold. The study also explored the CXCL12/CXCR4 axis and found that a CXCR4 antagonist promoted the restoration of the endothelium and, by mobilizing cells or altering the local signaling dynamics, facilitated the physical reconstruction of the vascular niche, thereby accelerating hematopoietic recovery. Furthermore, Zhu RJ et al. (2013) [77] found that cytarabine triggered massive adipogenesis, with the bone marrow being crowded with adipocytes, physically occupying the space needed for hematopoietic regeneration and secreting adipokines that negatively regulate hematopoiesis, through PPAR-γ activation. Crucially, they reported that BADGE-induced adipogenesis inhibition improved hematopoietic recovery. Wang J et al. (2018) [78] documented a severe reduction in both T and B lymphocyte populations and NK cell activity depression and described lienal peptide’s dual regulatory effects on immune reconstitution. Finally, Castañeda-Yslas IY et al. (2024) [76] assessed genotoxicity in the erythroid lineage and observed a spike in micronucleated erythrocytes, a definitive marker of chromosomal breakage during the erythroblast stage, while, also, evaluating silver nanoparticle protection. Yet, these studies, taken collectively, fail to coalesce into a unified model of cytarabine myelotoxicity.

Taken together, these diverse toxicities reveal cytarabine’s capacity to perturb multiple organ systems in ways that are neither incidental nor fully anticipated by current clinical monitoring (Table 10). The absence of systematic evaluation in domains such as cardiopulmonary and reproductive health reflects a persistent blind spot in translational research.

### 4.9. Limitations

Despite the comprehensive synthesis provided in this systematic review, several limitations warrant consideration. First, the methodological quality of the included animal studies was moderate at best, with consistent deficiencies in randomization, blinding and sample-size justification. Only a minority of studies reported random sequence generation, allocation concealment, or blinded outcome assessment, while none provided formal sample-size calculations. These omissions raise substantial concerns about internal validity, reproducibility and the risk of bias. Without adequate randomization and blinding, observed toxicities may reflect uncontrolled confounders or observer bias rather than true drug effects. Similarly, the absence of sample-size justification increases the likelihood of underpowered experiments, selective outcome reporting and exaggerated effect sizes. Second, although formal publication bias was not detected, the predominance of positive toxicity findings across studies strongly suggests the presence of reporting bias. Negative or null results may be underrepresented in the published literature, either due to selective reporting within studies or non-publication of inconclusive experiments. This skew toward positive outcomes inflates the apparent consistency and severity of cytarabine-induced toxicities, potentially overstating their translational relevance. The lack of transparency regarding unpublished data further limits the ability to assess the true spectrum of cytarabine’s systemic effects. Finally, heterogeneity in animal species, dosing regimens, routes of administration and outcome measures complicates cross-study comparison and limits extrapolation to human contexts. While body-surface area conversions provide approximate human equivalent doses, differences in metabolism, immune responses and developmental biology between species reduce the predictive value of these models.

### 4.10. Translational Implications and Future Perspectives

This systematic synthesis of preclinical evidence underscores several translational priorities that warrant consideration for clinical practice. Several biochemical and histopathological endpoints demonstrated consistent utility across studies and warrant clinical validation. Plasma citrulline, a potential marker of enterocyte mass, exhibited a dose-dependent reduction in animal models of cytarabine-induced mucositis and is already recognized clinically as a predictor of mucositis severity. Similarly, oxidative stress markers, such as MDA 4-HNE and 8-OHdG, were consistently elevated across multiple organ systems prior to overt histopathological damage, suggesting potential as early-warning indicators. Validation of these biomarkers in clinical cohorts receiving cytarabine therapy could enable timely intervention and personalized dose adjustment strategies.

Furthermore, preclinical evidence suggests that organ-specific toxicities exhibit distinct temporal profiles. Neurotoxicity and ocular injury manifested acutely (24–48 h post-administration), whereas hepatic and renal injuries developed subacutely (3–7 days). These temporal patterns imply that monitoring protocols should be tailored accordingly. For patients receiving high-dose cytarabine (≥2 g/m^2^), early neurological assessment (within 48 h), ophthalmological screening and, potentially, prophylactic corticosteroid eye drops and serial measurement of hepatic transaminases and renal function markers (starting day 3) may facilitate early detection and prompt intervention. Also, baseline assessment of cytidine deaminase (CDA) polymorphisms, known to influence ara-CTP accumulation, could inform individualized dosing strategies to mitigate toxicity risk.

Moreover, future clinical trials evaluating cytarabine regimens should incorporate prespecified toxicity endpoints aligned with preclinical observations. Standardized grading systems for cytarabine-specific toxicities, particularly neurotoxicity (cerebellar syndrome, cognitive impairment), gastrointestinal injury (mucositis severity, plasma citrulline) and ocular dysfunction (tear production, corneal fluorescein staining), should be implemented. It is also crucial that future research should prioritize underexplored domains such as cardiotoxicity, pulmonary injury and long-term reproductive outcomes, which remain insufficiently characterized in the current literature. Additionally, trials should stratify patients by known risk factors, including renal function, age and CDA polymorphism status, to enable subgroup analyses and identification of high-risk populations. Finally, mechanistic substudies incorporating molecular profiling, such as circulating microRNAs, proteomic and metabolomic signatures may elucidate early predictors of toxicity onset and inform adaptive dosing algorithms.

## 5. Conclusions

Preclinical data from 81 studies indicate that cytarabine induces dose- and regimen-dependent multi-organ toxicities in animal models, primarily affecting rapidly proliferating tissues through mechanisms such as oxidative stress, inflammation, apoptosis and cell-cycle disruption. Neurotoxicity was the most frequently observed adverse effect, followed by gastrointestinal mucositis, ocular injuries, alopecia, hepatotoxicity, nephrotoxicity and developmental anomalies. Pulmonary and reproductive toxicities were less studied and cardiotoxicity remains unassessed.

## Figures and Tables

**Table 1:** Population, Intervention, Comparator, Outcome table (PICO).

P (Population)	Preclinical animal models (mice, rats, rabbits, drosophila) of all species and breeds
I (Intervention)	Administration of cytosine arabinoside (Ara-C)
C (Comparator)	Control groups with no agent administered.
O (Outcome)	Main toxicity of cytarabine addressed; biochemical markers results (if available); histopathology results (if available); immunohistochemistry results (if available); proposed pathophysiological mechanisms (if available).

**Table 2:** Summary of Findings and Certainty of Evidence Table.

Outcome (Toxicity Type)	Number of Studies (Animals)	Summary of Findings		Certainty of Evidence (GRADE)	Reasons for Rating
		Primary Pathologies Observed	Key Mechanistic Drivers		
Neurotoxicity	23 (*n* ≈ 300–600; primarily mice and rats)	Cerebellar degeneration (Purkinje cell loss), dendritic atrophy, cognitive deficits, ataxia.	Oxidative stress, apoptosis (p53, caspase-3), epigenetic modification, NF-H degradation.	⊕⊕◯◯ Low	Downgraded one level for risk of bias (unclear randomization/blinding in >70% of studies) and one level for indirectness (preclinical models). No serious inconsistency or imprecision.
Gastrointestinal toxicity	11 (*n* ≈ 200–400; mice, rats, Drosophila)	Mucositis, villus atrophy/blunting, crypt necrosis, malabsorption, barrier failure.	Inflammation (TNF-α, IL-6), M1 macrophage polarization, oxidative stress, apoptosis.	⊕⊕◯◯ Low	Downgraded one level for risk of bias and one level for indirectness. Minor inconsistency in severity across doses; no serious imprecision.
Ocular toxicity	8 (*n* ≈ 150–300; mice, rats, rabbits)	Meibomian gland dysfunction, corneal epithelial erosion, retinal dysplasia.	Lipid metabolic dysregulation (PPARγ suppression), oxidative stress, stem cell depletion.	⊕⊕◯◯ Low	Downgraded one level for risk of bias and one level for indirectness. Consistent findings but small samples contribute to imprecision.
Alopecia	6 (*n* ≈ 100–200; primarily rats)	Macroscopic hair loss in 100% of exposed animals, with histopathology showing follicular inflammation.	Direct cytotoxicity to hair matrix cells, inflammation (IL-1 dependent).	⊕◯◯◯ Very low	Downgraded one level for risk of bias, one for indirectness, and one for imprecision (small cohorts, qualitative assessments). Potential publication bias.
Hepatotoxicity	4 (*n* ≈ 80–150; mice, rats, rabbits)	Hepatocellular necrosis, steatosis, fibrosis, enzyme elevation (ALT/AST).	Oxidative stress, G1/S cell cycle arrest (INK4 upregulation).	⊕◯◯◯ Very low	Downgraded one level for risk of bias, one for indirectness, and one for imprecision (few studies, sparse quantitative data).
Nephrotoxicity	3 (*n* ≈ 60–100; mice, rats)	Tubular necrosis, renal atrophy, developmental nephron deficit.	Inhibition of proliferation, developmental arrest, DNA synthesis inhibition.	⊕◯◯◯ Very low	Downgraded one level for risk of bias, one for indirectness, and one for imprecision (limited studies, small samples).
Developmental toxicity	27 (*n* ≈ 500–800; rodents, Drosophila)	Teratogenicity (limb defects, cleft palate), skeletal dysplasia, fetal growth restriction.	Wnt signaling suppression, massive apoptosis in limb buds, DNA synthesis inhibition.	⊕⊕◯◯ Low	Downgraded one level for risk of bias and one for indirectness. Some inconsistency in timing/dose effects; no serious imprecision despite overlaps.

**Table 3:** Neurotoxicity reported from individual studies.

Author, Year	Animal Model	Dosing Regimen	Primary Pathologies Observed	Key Mechanistic and Molecular Drivers
Alexander TC et al., 2018 [22]	Juvenile male C57BL/6 mice (P21)	5 mg/kg Ara-C + 10 mg/kg MTX, i.t. weekly × 3 wks	Cognitive impairment (MWM deficits); Dendritic atrophy in hippocampus; Selective loss of mature mushroom spines in DG, CA1, CA3.	Synaptic remodeling without necrosis; Dendritic retraction; Systemic and intrathecal inflammation (elevated leukocytes); Disruption of synaptic plasticity.
Fremouw T et al., 2012 [23]	Male C57BL/6J mice (8 wks)	275 mg/kg i.p. daily × 5 days	Significant weight loss (~9%); Numerical trends toward cognitive deficit (though not statistically significant in this specific protocol).	Systemic metabolic stress affecting cognition; Potential threshold effect for measurable memory decline.
Li CQ et al., 2008 [24]	Male Sprague–Dawley rats	400 mg/kg i.p. daily × 5 days	Impaired remote spatial memory; Apical dendritic retraction in Anterior Cingulate Cortex (shorter length, fewer branches/spines).	Selective dendritic remodeling in cortical layer II/III pyramidal neurons; Synaptic plasticity disruption in executive centers.
Patel RS et al., 2012 [27]	Juvenile Sprague–Dawley rats	50, 100, or 200 mg/kg i.p. daily for 5 or 14 days	Motor deficits (rotarod, gait); Purkinje cell misalignment/loss; Granule cell depletion in all lobes.	Oxidative stress (↑ MDA, ↓ GSH); DNA damage (Comet assay); Apoptosis (↑ p53, caspase-3); Epigenetic changes (↑ H3 acetylation, ↑ H3K4me, ↓ H3K9me).
Koros C et al., 2007 [29]	Adult male Wistar rats	400 mg/kg i.p. daily × 5 days	Ataxic gait; Rotarod deficits; Purkinje cell monolayer disruption; Granule cell cytotoxicity.	Cytoskeletal disruption (↓ Neurofilament in molecular layer); Dysregulation of calcium buffering (↑ Calbindin in Purkinje cells).
Koros C et al., 2009 [28]	Adult male Wistar rats	400 mg/kg i.p. daily × 5 days	Selective reduction in Neurofilament-Heavy (NF-H) isoform (~40%); Loss of axonal NF immunoreactivity.	Selective proteolysis or synthesis inhibition of NF-H isoform; Oxidative stress (mitigated by NAC).
Guzmán DC et al., 2018 [25]	Male Wistar rats (4 wks)	0.6 g/kg i.v. daily × 5 days	Neuronal pyknosis, cell shrinkage, vacuolization in cortex, striatum, cerebellum/medulla.	Neurotransmitter depletion (↓ Dopamine, ↓ 5-HIAA); Oxidative stress (↑ TBARS, ↓ GSH).
Guzmán DC et al., 2016 [26]	Male Wistar rats (~100 g)	70 mg/kg i.p. daily × 5 days	Biochemical alterations in brain regions (no histology).	Oxidative/Nitrosative stress; Monoamine depletion (↓ Dopamine); Na+, K+-ATPase inhibition.
Guzmán DC et al., 2024 [37]	Female Wistar rats (4 wks)	0.08 mM single i.p. injection	Altered dopamine levels in striatum/cerebellum; Oxidative stress markers.	Disruption of dopaminergic metabolism; Modulation of oxidative damage by oligoelements.
Salimi A et al., 2023 [31]	Adult male Wistar rats	70 mg/kg i.p. daily × 5 days	Midbrain neuronal loss; Cytoplasmic depletion; Granule layer fragmentation.	Mitochondrial dysfunction (↓ SDH, swelling, ROS generation, ΔΨm collapse); Oxidative stress; AChE/BChE inhibition.
Takano T et al., 2006 [32]	Pregnant ICR mice (offspring analyzed)	30 mg/kg i.p. on GD 13.5 and 14.5	Microcephaly; Gray-matter heterotopia; Disrupted cortical lamination; Subependymal nodules.	Massive apoptosis in proliferative zones (VZ/IZ); Failure of radial glial scaffold (↓ Nestin); Premature neuronal differentiation; Disrupted migration.
Yamauchi H et al., 2004 [33]	Pregnant Slc: Wistar rats	100 mg/kg i.p. daily × 7 days (GD13 assessment)	Neuroepithelial apoptosis in ventricular zone; Suppression of mitoses.	p53-dependent apoptosis (↑ p53 protein, ↑ p21/bax mRNA); Cell cycle arrest in fetal brain.
Shimada M et al., 1975 [34]	Newborn ICR-JCL mice	30 or 50 mg/kg s.c. on PND 2–4	Cerebellar hypoplasia; EGL necrosis; Heterotopic granule cells; Disorganized Purkinje layer.	Acute necrosis of External Granular Layer (EGL); Aberrant migration of regenerating granule cells; Persistent dysplasia.
Yamano T et al., 1980 [40]	ICR-JCL mouse pups	30 mg/kg s.c. on PND 2–4	Heterotopic granule cells in molecular layer; Disorganized Purkinje layer.	Synaptic mismatch (Granule cells synapse on mossy fibers in molecular layer); EGL disruption preventing normal migration.
Yamano T et al., 1983 [44]	ICR-JCL suckling mice	30 mg/kg s.c. daily × 3 days (various schedules)	Timing-dependent cerebellar dysplasia; Heterotopias; Purkinje dendritic arborization defects.	Critical window of EGL destruction determines severity of cytoarchitectural disarray; Purkinje cell defects secondary to granule cell loss.
Matsutani T et al., 1983 [39]	Wistar-Imamichi rats (Fetal or Neonatal)	280 mg/kg i.p. (Fetal) or 30 mg/kg s.c. (Neonatal)	Cerebellar hypoplasia; Ataxia; Altered DNA content and monoamine levels.	Growth retardation; Disrupted monoamine neurotransmitter development (↑ NE, 5-HT relative to tissue weight).
Kasubuchi Y et al., 1977 [41]	ICR-JCL mice	30 mg/kg i.p. on GD 13.3–14	Transplacental dysgenetic hydrocephalus; Matrix zone destruction; Cystic lesions.	S-phase specific necrosis of ventricular matrix cells; Ependymal denudation leading to aqueduct stenosis/hydrocephalus.
Percy DH et al., 1974 [35]	Mice and Rats (Postnatal)	3.125–50 mg/kg s.c. daily × 5 days	Cerebellar hypoplasia; Loss of lamination; Retinal/Renal dysplasia.	Inhibition of postnatal gliogenesis/neurogenesis; Dose-dependent arrest of developmental sequences.
Percy DH, 1975 [36]	Mice and Rats (Prenatal)	12.5–50 mg/kg s.c. daily × 3 days	Segmental cerebellar hypoplasia; Retinal dysplasia; Renal microcysts.	Stage-dependent teratogenicity matching organogenesis windows; Cellular necrosis in proliferative zones.
Narang HK, 1982 [38]	New Zealand albino rabbits	10–20 mg/kg s.c. (various schedules)	Optic nerve atrophy; Spongy degeneration; Gliosis; Neurologic signs (ataxia).	Macrophage infiltration; Demyelination/remyelination attempts; Synergistic toxicity with HSV infection context.
Elmer GI et al., 2004 [43]	Pregnant Sprague–Dawley rats	30 mg/kg i.p. on GD 19.5 & 20.5	Adult-onset sensorimotor gating deficits (PPI); Subtle hippocampal disorganization.	Disruption of late-stage neurogenesis; Dopaminergic/Glutamatergic signaling perturbation (schizophrenia model).
Adlard BP et al., 1975 [42]	Lister hooded rats	50 mg/kg (prenatal) or 250 mg/kg (postnatal)	Impaired brain growth; Cerebellar stunting; Learning deficits in T-maze.	Growth retardation; Permanent cognitive sequelae from developmental insult; Toxicity > Adenine Arabinoside (Ara-A).
Guan Z et al., 2023 [30]	Pregnant C57BL/6 mice	Single i.p. 22.5 mg/kg (optimal for NTD)	Neural Tube Defects (Exencephaly); Disorganized neuroepithelium.	Suppression of Wnt/β-catenin signaling; Premature differentiation (Gliogenic shift); Apoptosis (Caspase-3).

**Table 4:** Gastrointestinal toxicity reported from individual studies.

Author, Year	Animal Model	Dosing Regimen	Primary Pathologies Observed	Key Mechanistic and Molecular Drivers
Li JJ et al., 2023 [46]	Male C57BL/6 mice	100 mg/kg i.p. daily × 7 days	Ileal villus shortening/flattening; Crypt necrosis; Epithelial vacuolation; Muscularis edema; Weight loss.	M1 Macrophage polarization (↑ CD86, iNOS); Pro-inflammatory cytokines (↑ TNF-α, IL-6, ↓ IL-10); AKT signaling suppression; Tight junction loss (↓ ZO-1/Occludin).
de Souza Silva PM et al., 2018 [45]	BALB/c mice	1.8 mg/mouse i.p. q12h × 4 doses	50% loss of mitotic activity in crypts; Leukopenia; DNA damage in leukocytes.	Genotoxicity (DNA strand breaks); Mitotic arrest in crypt progenitors; Synergism with dietary modulation.
Ramos MG et al., 1997 [47]	Swiss NMRI mice	3.6 mg/day i.p. × 2–4 days	Villus shortening; Enterocyte necrosis; Lamina propria inflammation; Hepatic necrosis.	Nutritional modulation (worsened by elemental diet); SCFA deficiency; Inflammation.
Ramos MG et al., 1999 [48]	Germ-free mice	3.6 mg/day i.p. × 2 days	Severe villus shortening; Enterocyte loss/necrosis; Inflammation.	Mucosal atrophy independent of microbiome; Mitigation by exogenous SCFAs; Epithelial necrosis.
Chu W et al., 2023 [49]	Male C57BL/6 mice	100 mg/kg i.p. daily × 7 days	Villus atrophy; Crypt necrosis; Epithelial vacuolization; Edema; Anorexia.	M1 Macrophage polarization via JAK2/STAT1 signaling; Inflammation (↑ TNF-α, IL-6, ↓ IL-10); iNOS activation.
Minden MD et al., 2024 [51]	Male BALB/c mice	30 mg/kg i.p. BID × 5 days (High toxicity model)	100% mortality by Day 3; Severe crypt/villus destruction; Dysbiosis.	Enterocyte mass depletion (↓ Plasma Citrulline); Gut microbiota shift (↓ Firmicutes, ↑ Bacteroidetes); Rescue by GLP-2 analog.
Park M-R et al., 2023 [104]	Male C57BL/6 mice	100 mg/kg i.v. daily × 4 days	Cachexia with lipid malabsorption (retained fecal lipids); Chylomicron retention in enterocytes.	Lymphatic vessel remodeling (Lacteal junction “zippering” via VE-cadherin); VEGFR2/AKT phosphorylation; Impaired lipid transport; Mitochondrial/Golgi stress.
Porsani MYH et al., 2017 [52]	Male Balb/C mice	15 mg/kg i.p. q12h × 4 doses	Reduced villus height/width and crypt depth; Leukopenia.	Immune suppression (↓ IL-10, ↑ IFN-γ); Synergistic protection by β-glucan/glutamine.
Elli M et al., 2009 [53]	Male BALB/c mice	3.6 mg/mouse i.p. daily × 5 days	Marked villus atrophy; Crypt hyperplasia; Inflammatory infiltrate.	Mucosal injury mitigated by Vitamin A; Enterocyte loss.
Chwalinski S et al., 1989 [54]	Male BDF1 mice	200 + 100 µg/g i.p. (12 h apart)	Selective destruction of crypt cells above Paneth zone; Paneth cells spared.	S-phase specific cytotoxicity; Regeneration originates from surviving Paneth/stem cell zone; Spatial specificity of apoptosis.
Han S et al., 2023 [55]	*Drosophila melanogaster*	1–10 mM in food (continuous)	Midgut shortening; Epithelial edema; Microvilli truncation; Mitochondrial rupture.	Oxidative stress (↑ ROS, GST); Innate immune activation (Toll, IMD); Apoptosis (Reaper, Drice); JAK-STAT/JNK signaling upregulation.
Chen T, 1982 [56]	Male Swiss-Webster mice	50 mg/kg i.p. daily × 5 days	Impaired active transport of Glucose, Amino acids, Electrolytes (Na+, Cl−).	Functional transporter defect; Reduced transmucosal potential difference; Altered mucosal electrophysiology.

**Table 5:** Ocular toxicity reported from individual studies.

Author, Year	Animal Model	Dosing Regimen	Primary Pathologies Observed	Key Mechanistic and Molecular Drivers
Liu et al., 2024 [57]	Male C57BL/6J mice	50 mg/kg i.p. daily × 7 days	Meibomian Gland Dysfunction (plugging, atrophy); Lacrimal gland hyposecretion; Corneal epithelial defects.	Suppression of PPARγ signaling; Altered lipid metabolism (↓ AWAT2, ↑ Cholesterol); Ductal hyperkeratinization (↑K1/K10); Oxidative stress (↑ 4-HNE, ↓ Nrf2); ↓ AKT/FoxO signaling.
Balci YI et al., 2017 [58]	Mature Wistar rats	400 mg/kg i.p. daily × 5 days	Oxidative stress in cornea/conjunctiva (Biochemical assessment).	Oxidative injury (↑ Total Oxidant Status, ↑ Oxidative Stress Index); Mitigated by NAC.
Rootman J et al., 1983 [59]	Female NZ White rabbits	37.5 mg/kg Subconjunctival vs. IV	Focal superficial conjunctival erosions; Transient infiltrates (Subconjunctival route).	Local toxicity due to high tissue concentration (15× higher in anterior chamber vs. IV); Polymorphonuclear infiltration; Route-dependent toxicity.
Diets-Ouwehand J et al., 1992 [60]	Chinchilla rabbits	600–2700 µg Intravitreal	Blood–retina barrier leakage (Fluorescein); ERG deficits (b-wave reduction); Synaptic pedicle disorganization (EM).	Retinal neurotoxicity at high local doses; Synaptic vesicle disorganization; Barrier breakdown; Photoreceptor dysfunction.
Percy DH et al., 1977 [61]	Newborn Sprague–Dawley rats	15 mg/kg s.c. PND 1–5	Retinal dysplasia; Rosette formation; Photoreceptor misalignment; Pigment epithelium reaction.	Disruption of postnatal retinal histogenesis; Phagocyte infiltration; Persistent cellular degeneration; Bipolar cell displacement.
Shimada M et al., 1973 [62]	Newborn ICR-JCL mice	30–50 mg/kg s.c. PND 2–4	Widespread rosette formation in outer nuclear layer; Heterotopic ganglion cells.	Selective necrosis of undifferentiated outer nuclear layer; Failure of differentiation; Heterotopic migration.
Kaufman HE et al., 1964 [63]	NZ White rabbits	1.0% Ophthalmic drops q2h × 5 days	Corneal epithelial opacities (“glittering”); Punctate staining; Iritis.	Inhibition of DNA synthesis in corneal epithelium; Megaloblastic epithelial changes; Loss of glycolytic enzymes; Basal cell toxicity.
Percy DH, 1975 [36]	Rats (Prenatal exposure)	50 mg/kg s.c. GD 18–20	Retinal dysplasia; Central retinal thinning; Rosette formation.	Teratogenic disruption of late-fetal retinal layering; Dose-dependent severity.

**Table 6:** Alopecia reported from individual studies.

Author, Year	Animal Model	Dosing Regimen	Primary Pathologies Observed	Key Mechanistic and Molecular Drivers
Jimenez JJ et al., 1992 [64]	Fisher rats (7 days old)	20 mg/kg i.p. daily × 7 days	Complete body alopecia (100% incidence).	Direct cytotoxicity to hair matrix; Protection by IL-1β (induced quiescence/G1 arrest).
Jimenez JJ et al., 1992 [65]	Sprague-Dawley rats	50 mg/kg Ara-C + 50 mg/kg CTX	100% alopecia (macroscopic).	Synergistic toxicity with cyclophosphamide; Protection by ImuVert/NAC (Antioxidant/Immune modulation).
Sun B et al., 2009 [67]	Sprague–Dawley rat pups	30 mg/kg i.p. daily × 7 days	Severe alopecia (75–100% loss); Follicle atrophy; Loss of follicle number.	Hair follicle toxicity mitigated by AHCC (immunostimulant/antioxidant).
Hussein AM, 1995 [68]	Sprague–Dawley rat pups	75 mg/kg i.p. daily × 5 days	100% complete alopecia.	Hair follicle arrest; Protection by Minoxidil (Topical or SC).
Hagiwara S et al., 2011 [69]	Wistar rat pups	20 mg/kg i.p. daily × 7 days	Complete alopecia; Dense inflammatory infiltrates in follicles.	Mitochondrial damage (swelling, disrupted cristae) in hair matrix cells; Inflammation; Protection by zinc/lipoic acid derivative.
Jimenez JJ et al., 1992 [66]	Rats	In vitro and In vivo	Protection of hair follicles.	Mechanism confirmation: IL-1 protects by arresting cells in G1, evading S-phase toxicity.

**Table 7:** Hepatotoxicity reported from individual studies.

Author, Year	Animal Model	Dosing Regimen	Primary Pathologies Observed	Key Mechanistic and Molecular Drivers
Kolure R et al., 2023 [71]	Pregnant Sprague Dawley rats	25 mg/kg p.o. daily GD 8–20	Hepatic vacuolization; Disrupted lobular architecture; Pycnotic nuclei; Sinusoidal dilation.	Oxidative stress (↑ MDA, ↓ SOD/CAT/GSH); Serum enzyme elevation (AST/ALT/ALP); Maternal toxicity.
Saif A-J et al., 2020 [72]	NZ White rabbits	50 mg/kg i.p. daily × 7 days	Coagulative necrosis of periportal hepatocytes; Portal fibrosis; Bile duct hyperplasia; Congestion.	Inflammatory infiltration (Mononuclear/Kupffer cells); Fibrotic remodeling; Sinusoidal distension.
Dudina MO et al., 2018 [73]	Wistar rats	2 g/m^2^ i.v. daily × 5 days	Centrilobular necrosis; Steatosis; Portal fibrosis; Vacuolization; Karyolysis.	Pro-inflammatory cytokines (↑ TNF-α, ↓ IL-10); Apoptosis/Survival imbalance (↑ Bcl-2, ↓ Ki-67); ↑ HGF (repair response); Enzyme leakage.
Sun F et al., 2019 [70]	BALB/c nude mice	2.5 mg/kg i.p. daily × 20 days	Liver atrophy; Weight loss.	G1/S cell cycle arrest; Upregulation of INK4 family inhibitors (CDKN2A–D); Suppression of CDK4/Cyclin D1 complex.

**Table 8:** Renal toxicity reported from individual studies.

Author, Year	Animal Model	Dosing Regimen	Primary Pathologies Observed	Key Mechanistic and Molecular Drivers
Sun F et al., 2019 [70]	BALB/c nude mice	2.5 mg/kg i.p. daily × 20 days	Renal atrophy; Inflammation; Structural disruption.	Induction of cell cycle inhibitors (INK4 family); Impaired tissue growth/maintenance; CDK4/Cyclin D1 suppression.
Percy DH et al., 1974 [35]	Mice and Rats (Postnatal)	3.125–50 mg/kg s.c. PND 1–5	Focal cortical dysplasia; Subcapsular nests of primordial cells; Glomerular arrest.	Arrest of postnatal nephrogenesis; Inhibition of DNA synthesis in cortex; Dose-dependent dysplasia.
Percy DH, 1975 [36]	Rats (Prenatal)	50 mg/kg s.c. GD 18–20	Focal subcapsular microcysts; Dilated tubules; Vacuolated epithelium.	Teratogenic disruption of renal development; Cystogenesis; Primordial cell nests indicating arrest.

**Table 9:** Developmental toxicity reported from individual studies.

Author, Year	Animal Model	Dosing Regimen	Primary Pathologies Observed	Key Mechanistic and Molecular Drivers
Namoju R et al., 2021 [80]	Pregnant rats	12.5–25 mg/kg i.p. GD 8–14	Limb reduction defects; Oligodactyly; Impaired ossification; Resorptions.	Placental oxidative stress (↑ MDA, ↓ GSH); Maternal toxicity; Reduced bone Ca/P content.
Zhao X et al., 2020 [81]	Pregnant Sprague–Dawley rats	100 mg/kg i.p. single dose (GD 11–14)	Thumb polydactyly/syndactyly (peak at GD 12.5); Extra metacarpals.	Expansion of FGF4 expression domain in limb bud; Persistence of AER; Disrupted patterning.
Chilaka KN et al., 2024 [82]	Pregnant rats	12.5–25 mg/kg i.p. GD 8–21	F1 male reproductive toxicity (Testicular atrophy, sperm defects).	Oxidative stress in fetal testis; Hormonal disruption (↓ Testosterone, FSH, LH); Sertoli/Leydig cell damage; Altered steroidogenic enzymes.
Yamauchi H et al., 2003 [83]	Pregnant Wistar rats	250 mg/kg i.p. GD 13	Fetal hypoplasia; Placental labyrinth thinning; Widespread apoptosis.	Acute apoptosis in neuroepithelium and mesenchyme (3–12 h post dose); TUNEL+ cells in multiple fetal tissues.
Kochhar DM et al., 1978 [84]	Pregnant ICR mice	2–200 mg/kg i.p. GD 10.5–12	Limb defects (micromelia to adactyly); Embryolethality.	DNA synthesis inhibition; Mesenchymal necrosis; Stage-dependent phenotype; AER thickening.
Manson JM et al., 1977 [85]	Pregnant ICR mice	40 mg/kg i.p. GD 10–12	Limb blisters; Adactyly.	Mesenchymal necrosis; Blisters represent fluid accumulation over necrotic tissue; In vitro correlation.
Rahman ME et al., 1994 [86]	Pregnant ICR mice	5 mg/kg i.p. GD 10.5	Carpal/Tarsal bone fusions and absence; Oligodactyly.	Specific sensitivity of carpal/tarsal ossification centers; Correlation with digit defects.
Rahman ME et al., 1996 [87]	Pregnant ICR mice	0.5–2.0 mg/kg i.p. GD 10.5	Carpal/Tarsal anomalies (independent of digit defects).	High sensitivity of wrist/ankle bones to antiproliferative agents compared to digits.
Chiang H et al., 1995 [88]	Pregnant Swiss mice	10 mg/kg i.p. GD 9	Cleft palate; Cleft lip; Resorptions.	Synergism with pulsed magnetic fields enhancing teratogenicity.
Chiba K et al., 1996 [89]	Pregnant ICR mice	5–7.5 mg/kg i.p. GD 8–11	Hip joint anomalies (dysplasia, pseudoarthrosis); Femoral defects.	Disruption of hip/femur ossification centers; Co-occurrence with hindlimb defects.
Ritter EJ et al., 1971 [90]	Pregnant Wistar rats	25–200 mg/kg i.p. GD 12	Limb malformations; Growth retardation; Resorptions.	Correlation between duration of DNA synthesis inhibition and teratogenicity severity.
Ritter EJ et al., 1973 [91]	Pregnant Wistar rats	200 mg/kg (Ara-CP) GD 12	Ectrodactyly.	Delayed/Prolonged DNA synthesis inhibition by palmitate ester leading to specific limb defects.
Scott WJ et al., 1975 [92]	Pregnant rats	100 mg/kg i.p. GD 10–11	Polydactyly (Preaxial).	Synchronized S-phase peaks; Lack of normal preaxial cell death zone; Ectodermal ridge thickening.
Goto T et al., 1987 [93]	Pregnant ICR mice	2.5–10 mg/kg i.p. GD 9.5–10.5	Polydactyly/Oligodactyly.	Sex differences in susceptibility (Males > Females for oligodactyly); Dose-dependent shift in lesion type.
Endo A et al., 1987 [94]	Pregnant CD-1 mice	5 mg/kg i.p. GD 11	Digit defects; Cleft palate.	Circadian susceptibility.
Rahman ME et al., 1995 [95]	Pregnant ICR mice	5 mg/kg i.p. GD 9.5–12.5	Carpal/Tarsal fusions.	Broad critical period for carpal/tarsal defects compared to digits.
Chaube S et al., 1968 [96]	Pregnant Wistar rats	2.5–900 mg/kg i.p. GD 5–12	Cleft palate; Encephalocele; Limb deformities.	Prevention by Deoxycytidine (CdR); Lack of deaminase in embryo leads to high drug exposure.
Guan Z et al., 2023 [30]	Pregnant C57BL/6 mice	22.5 mg/kg i.p. GD 7.5	Neural Tube Defects (Exencephaly); Growth retardation.	Inhibition of Wnt/β-catenin; Gliogenic shift; Apoptosis (Caspase-3).
Yamauchi H et al., 2004 [101]	Wistar Pregnant rats	250 mg/kg i.p. single on GD13	Placental injury: labyrinth zone trophoblastic cell apoptosis, labyrinth thinning	p53 peak at 1–3 h; Proliferation fell by 3–6 h; Apoptosis (TUNEL, caspase-3) at 6 h; p21, cyclin G1, fas mRNAs peak at 9 h

**Table 10:** Other reported toxicities from individual studies.

Author, Year	Animal Model	Dosing Regimen	Primary Pathologies Observed	Key Mechanistic and Molecular Drivers
Bilgin AO et al., 2020 [79]	Male Wistar rats	200 mg/kg i.p. daily × 14 days	Pulmonary edema; Alveolar hemorrhage; Inflammation.	Lung oxidative stress (↑ MDA, ↓ GSH); Inflammation (↑ TNF-α, NF-κB); Mitigated by Rutin.
Saif A-J et al., 2024 [50]	NZ White rabbits	60 mg/kg i.p. daily × 10 days	Parotid gland acinar necrosis; Ductal necrosis; Stromal thickening.	Inflammation (↑ TNF-α); Apoptosis (Bcl-2 modulation); Fibrosis.
Khaleel B et al., 2022 [118]	Juvenile C57BL/6 mice	140 mg/kg i.p. × 3 doses	Testicular atrophy; Seminiferous tubule apoptosis; Permanent Loss of spermatogonia.	Niche disruption: ↓ GDNF, ↓ SCF, ↓ IL-10.
Orth JM et al., 1988 [119]	Rat pups (PND 2)	Intratesticular injection	Reduced adult Sertoli cell number (−54%); Reduced testis size.	Selective inhibition of Sertoli cell proliferation in neonatal period; ↓ ABP.
Watanabe S et al., 1992 [120]	Male Sprague–Dawley rats	63 mg/kg s.c. daily × 5 days	Atrophy of prostate and seminal vesicles; Weight loss.	Polyamine dysregulation (↑ Putrescine/Spermidine ratio).
Palo AK et al., 2009 [74]	Swiss albino mice	100–200 mg/kg i.p. single	Bone marrow chromosomal aberrations; Germ cell genotoxicity.	Clastogenicity (Breaks, fragments); Micronuclei formation.
Lee JY et al., 2018 [75]	C57BL/6J mice	100 mg/kg i.p. single	Bone marrow sinusoidal collapse; Megakaryocyte depletion.	Vascular niche disruption; CXCL12/CXCR4 axis.
Zhu RJ et al., 2013 [77]	Female C57BL/6J mice	0.5 g/kg i.p. daily × 4 days	Marrow adipogenesis (Fatty marrow); Sinus dilation; Hemorrhage.	Adipocyte hyperplasia via PPARγ; Inhibition of hematopoiesis by fatty marrow; Rescued by PPARγ antagonist.
Wang J et al., 2018 [78]	Male C57BL/6 mice	250 mg/kg i.p. daily × 3 days	Immunosuppression (Lymphoid depletion (T/B/NK)).	Functional immune impairment.
Castañeda-Yslas IY et al., 2024 [76]	Male BALB/c mice	6 mg/kg i.p. (single or ×3)	Myelosuppression; Genotoxicity (Micronucleated erythrocytes).	Genotoxicity; DNA strand breaks
Cano F et al., 2008 [121]	Translocator mice	100 mg/kg i.v. daily × 5 days	Granulocyte clearance; Spleen size normalization (Efficacy model).	Therapeutic cytoreduction; Used to validate leukemia model efficacy.

## Data Availability

No new data were created or analyzed in this study. Data sharing is not applicable to this article.

## Supplementary Materials

The following supporting information can be downloaded at: https://www.mdpi.com/article/10.3390/cimb48010004/s1.

## Abbreviations

The following abbreviations are used in this manuscript:

↓	Decreased
↑	Increased
AML	Acute Myeloid Leukemia
Ara-C	Cytarabine (Cytosine Arabinoside)
ara-CTP	Cytarabine Triphosphate
CDA	Cytidine Deaminase
i.p.	Intraperitoneal
i.v.	Intravenous
s.c.	Subcutaneous
i.t.	Intrathecal
GD	Gestational Day
MWM	Morris Water Maze
ACC	Anterior Cingulate Cortex
DG	Dentate Gyrus
CA1/CA3	Cornu Ammonis regions of hippocampus
NF	Neurofilament
NF-H/NF-M/NF-L	Neurofilament Heavy/Medium/Light isoforms
MDA	Malondialdehyde
GSH	Glutathione (reduced)
GSSG	Glutathione disulfide (oxidized)
SOD	Superoxide Dismutase
CAT	Catalase
GSH-Px	Glutathione Peroxidase
TBARS	Thiobarbituric Acid Reactive Substances
ROS	Reactive Oxygen Species
ΔΨm	Mitochondrial Membrane Potential
TUNEL	Terminal deoxynucleotidyl transferase dUTP Nick End Labeling
H3K9me	Histone H3 Lysine 9 Methylation
H3K4me	Histone H3 Lysine 4 Methylation
H3 acetylation	Histone H3 Acetylation
GFAP	Glial Fibrillary Acidic Protein
PH3	Phospho-Histone H3
Wnt	Wingless/Integrated signaling pathway
JAK-STAT	Janus Kinase—Signal Transducer and Activator of Transcription
JNK	c-Jun N-terminal Kinase
IMD	Immune Deficiency pathway (Drosophila)
TNF-α	Tumor Necrosis Factor alpha
IL-6/IL-10	Interleukin-6/Interleukin-10
PPARγ	Peroxisome Proliferator-Activated Receptor gamma
AQP5	Aquaporin 5
α-SMA	Alpha-Smooth Muscle Actin
PCNA	Proliferating Cell Nuclear Antigen
ZO-1	Zonula Occludens-1
VE-cadherin	Vascular Endothelial Cadherin
VEGFR2	Vascular Endothelial Growth Factor Receptor 2
AKT	Protein Kinase B
FoxO1/FoxO3a	Forkhead Box O1/O3a
Keap1	Kelch-like ECH-associated protein 1
Nrf2	Nuclear Factor Erythroid 2–Related Factor 2
HO-1	Heme Oxygenase-1
OSI	Oxidative Stress Index
TOS	Total Oxidant Status
AST	Aspartate Aminotransferase
ALT	Alanine Aminotransferase
ALP	Alkaline Phosphatase
GGTP	Gamma-Glutamyl Transpeptidase
HGF	Hepatocyte Growth Factor
Bcl-2	B-cell lymphoma 2 (anti-apoptotic protein)
NOS	Newcastle–Ottawa Scale
ARRIVE	Animal Research: Reporting of In Vivo Experiments
SYRCLE	Systematic Review Centre for Laboratory Animal Experimentation
CAMARADES	Collaborative Approach to Meta-Analysis and Review of Animal Data from Experimental Studies
PRISMA	Preferred Reporting Items for Systematic Reviews and Meta-Analyses
PROSPERO	International Prospective Register of Systematic Reviews
GRADE	Grading of Recommendations Assessment, Development and Evaluation
PICO	Population, Intervention, Comparator, Outcome
EM	Electron Microscopy
IHC	Immunohistochemistry
H&E	Hematoxylin and Eosin
SCF	Stem Cell Factor
GDNF	Glial cell line-Derived Neurotrophic Factor
MCSF	Macrophage Colony-Stimulating Factor
FSH	Follicle-Stimulating Hormone
LH	Luteinizing Hormone
3β-HSD/17β-HSD	3β-/17β-Hydroxysteroid Dehydrogenase
ABP	Androgen-Binding Protein
NAC	N-Acetylcysteine
AHCC	Active Hexose Correlated Compound
ALA	Alpha-Lipoic Acid
AS-IV	Astragaloside IV
GQBZP	Guiqi Baizhu Prescription
LP	Lienal Peptide
AgNPs	Silver Nanoparticles
BADGE	Bisphenol A Diglycidyl Ether (PPARγ antagonist)
CPX-351	Liposomal Cytarabine + Daunorubicin formulation
HU	Hydroxyurea
Ara-CP	Cytarabine Palmitate (long-acting formulation)
Ara-A	Adenine Arabinoside
Ara-U	Uracil Arabinoside
CdR	Deoxycytidine
dCMP/CMP/CDP/CR/TdR	Deoxycytidine Monophosphate/Cytidine Monophosphate/Cytidine Diphosphate/Cytidine/Thymidine
5-aza-C	5-Azacytidine
3MG	3-O-Methylglucose
NE	Norepinephrine
DA	Dopamine
5-HT	Serotonin
5-HIAA	5-Hydroxyindoleacetic Acid
CNPase	2′,3′-Cyclic-Nucleotide 3′-Phosphodiesterase
MAP-2	Microtubule-Associated Protein 2
VZ	Ventricular Zone
IZ	Intermediate Zone
GE	Ganglionic Eminence
EGL	External Granular Layer
IGL	Internal Granular Layer
ML	Molecular Layer
BM	Bone Marrow
HSC	Hematopoietic Stem Cell
HPC	Hematopoietic Progenitor Cell
CFU	Colony-Forming Unit
WBC	White Blood Cell
Hb	Hemoglobin
TAS	Total Antioxidant Status
tGSH	Total Glutathione
NF-κB	Nuclear Factor kappa-light-chain-enhancer of activated B cells
SDS	Sodium Dodecyl Sulfate
TEM	Transmission Electron Microscopy
EB	Embryoid Body
mESC	Mouse Embryonic Stem Cell
CFS	Corneal Fluorescein Staining
AWAT2	Acyl-CoA:Wax Alcohol Acyltransferase 2
SOAT1	Sterol O-Acyltransferase 1
ELOVL4	Elongation of Very Long Chain Fatty Acids Protein 4
HMGCR	3-Hydroxy-3-Methylglutaryl-CoA Reductase
SDH	Succinate Dehydrogenase
AER	Apical Ectodermal Ridge
SCFAs	Short-Chain Fatty Acids

## References

[1] Di Francia R., Crisci S., De Monaco A., Cafiero C., Re A., Iaccarino G., De Filippi R., Frigeri F., Corazzelli G., Micera A. (2021). Response and Toxicity to Cytarabine Therapy in Leukemia and Lymphoma: From Dose Puzzle to Pharmacogenomic Biomarkers. Cancers.

[2] Li A., Fridley B., Kalari K., Jenkins G., Batzler A., Safgren S., Hildebrandt M., Ames M., Schaid D., Wang L. (2008). Gemcitabine and Cytosine Arabinoside Cytotoxicity: Association with Lymphoblastoid Cell Expression. Cancer Res..

[3] Gökbuget N., Hoelzer D. (2009). Treatment of Adult Acute Lymphoblastic Leukemia. Semin. Hematol..

[4] Friedberg J.W. (2011). Relapsed/Refractory Diffuse Large B-Cell Lymphoma. Hematology/the Education Program of the American Society of Hematology. Am. Soc. Hematol. Educ. Program.

[5] Burnett A., Wetzler M., Löwenberg B. (2011). Therapeutic Advances in Acute Myeloid Leukemia. J. Clin. Oncol..

[6] Evrard A., Lacarelle B., Ciccolini J. (2013). Severe or Lethal Toxicities with Nucleosidic Analogs: Time for Action. Pharmacogenomics.

[7] Chagnon K., Boissel N., Raffoux E., Dombret H., Tazi A., Bergeron A. (2009). A New Pattern of Cytosine-Arabinoside-Induced Lung Toxicity. Br. J. Haematol..

[8] Ciccolini J., Evrard A., M’Batchi L., Pourroy B., Mercier C., Iliadis A., Lacarelle B., Verschuur A., Ouafik L., André N. (2012). CDA Deficiency as a Possible Culprit for Life-Threatening Toxicities after Cytarabine plus 6-Mercaptopurine Therapy: Pharmacogenetic Investigations. Pharmacogenomics.

[9] Baker W.J., Royer G.L., Weiss R.B. (1991). Cytarabine and Neurologic Toxicity. J. Clin. Oncol..

[10] Mayer R.J., Davis R.B., Schiffer C.A., Berg D.T., Powell B.L., Schulman P., Omura G.A., Moore J.O., McIntyre O.R., Frei E. (1994). Intensive Postremission Chemotherapy in Adults with Acute Myeloid Leukemia. N. Engl. J. Med..

[11] McGrail L.H., Sehn L.H., Weiss R.B., Robson M.R., Antin J.H., Byrd J.C. (1999). Pancreatitis during Therapy of Acute Myeloid Leukemia: Cytarabine Related?. Ann. Oncol..

[12] McBride C.E., Yavorski R.T., Moses F.M., Robson M.E., Solimando D.A., Byrd J.C. (1996). Acute Pancreatitis Associated with Continuous Infusion Cytarabine Therapy: A Case Report. Cancer.

[13] Powell B.L., Zekan P.J., Muss H.B., Richards F., Lyerly E.S., Capizzi R.L. (1986). Ara-C Syndrome during Low-dose Continuous Infusion Therapy. Med. Pediatr. Oncol..

[14] Moore J.O., George S.L., Dodge R.K., Amrein P.C., Powell B.L., Kolitz J.E., Baer M.R., Davey F.R., Bloomfield C.D., Larson R.A. (2005). Sequential Multiagent Chemotherapy Is Not Superior to High-Dose Cytarabine Alone as Postremission Intensification Therapy for Acute Myeloid Leukemia in Adults under 60 Years of Age: Cancer and Leukemia Group B Study 9222. Blood.

[15] Konstantinidis I., Tsokkou S., Grigoriadis S., Chrysavgi L., Gavriilaki E. (2024). Cardiotoxicity in Acute Myeloid Leukemia in Adults: A Scoping Study. Cancers.

[16] Nagahata Y., Kondo T., Ono Y., Hiramoto N., Kitano T., Hishizawa M., Yamashita K., Hashimoto H., Ishikawa T., Takaori-Kondo A. (2020). High-Dose Cytarabine Chemotherapy (≥4 g/M2/Day) before Allogeneic Hematopoietic Stem Cell Transplantation for Non-Core-Binding-Factor AML in the First Complete Remission. Leuk. Lymphoma.

[17] Page M.J., McKenzie J.E., Bossuyt P.M., Boutron I., Hoffmann T.C., Mulrow C.D., Shamseer L., Tetzlaff J.M., Akl E.A., Brennan S.E. (2021). The PRISMA 2020 Statement: An Updated Guideline for Reporting Systematic Reviews. BMJ.

[18] Richardson W.S., Wilson M.C., Nishikawa J., Hayward R.S. (1995). The Well-Built Clinical Question: A Key to Evidence-Based Decisions. ACP J. Club.

[19] Hooijmans C.R., Rovers M.M., De Vries R.B.M., Leenaars M., Ritskes-Hoitinga M., Langendam M.W. (2014). SYRCLE’s Risk of Bias Tool for Animal Studies. BMC Med. Res. Methodol..

[20] Bahor Z., Liao J., Currie G., Ayder C., MacLeod M., McCann S.K., Bannach-Brown A., Wever K., Soliman N., Wang Q. (2021). Development and Uptake of an Online Systematic Review Platform: The Early Years of the CAMARADES Systematic Review Facility (SyRF). BMJ Open Sci..

[21] Wells G.A., Shea B., O’Connell D., Peterson J., Welch V., Losos M., Tugwell P. The Newcastle-Ottawa Scale (NOS) for Assessing the Quality of Nonrandomised Studies in Meta-Analyses. https://ohri.ca/en/who-we-are/core-facilities-and-platforms/ottawa-methods-centre/newcastle-ottawa-scale.

[22] Alexander T.C., Simecka C.M., Kiffer F., Groves T., Anderson J., Carr H., Wang J., Carter G., Allen A.R. (2018). Changes in Cognition and Dendritic Complexity Following Intrathecal Methotrexate and Cytarabine Treatment in a Juvenile Murine Model. Behav. Brain Res..

[23] Fremouw T., Fessler C.L., Ferguson R.J., Burguete Y. (2012). Recent and Remote Spatial Memory in Mice Treated with Cytosine Arabinoside. Pharmacol. Biochem. Behav..

[24] Li C.Q., Liu D., Huang L., Wang H., Zhang J.Y., Luo X.G. (2008). Cytosine Arabinoside Treatment Impairs the Remote Spatial Memory Function and Induces Dendritic Retraction in the Anterior Cingulate Cortex of Rats. Brain Res. Bull..

[25] Guzmán D.C., Herrera M.O., Peraza A.V., Brizuela N.O., García E.H., Olguín H.J., Mejía G.B., del Ángel D.S., Ochoa A.R. (2018). Cambios Bioquímicos e Histológicos Producidos Por Edulcorantes y Citarabina En El Cerebro de Ratas Jóvenes. Nutr. Hosp..

[26] Calderón Guzmán D., Osnaya Brizuela N., Ortíz Herrera M., Juárez Olguín H., Hernández García E., Valenzuela Peraza A., Barragán Mejía G. (2016). Oleic Acid Protects Against Oxidative Stress Exacerbated by Cytarabine and Doxorubicin in Rat Brain. Anticancer Agents Med. Chem..

[27] Patel R.S., Rachamalla M., Chary N.R., Shera F.Y., Tikoo K., Jena G. (2012). Cytarabine Induced Cerebellar Neuronal Damage in Juvenile Rat: Correlating Neurobehavioral Performance with Cellular and Genetic Alterations. Toxicology.

[28] Koros C., Kitraki E. (2009). Neurofilament Isoform Alterations in the Rat Cerebellum Following Cytosine Arabinoside Administration. Toxicol. Lett..

[29] Koros C., Papalexi E., Anastasopoulos D., Kittas C., Kitraki E. (2007). Effects of AraC Treatment on Motor Coordination and Cerebellar Cytoarchitecture in the Adult Rat: A Possible Protective Role of NAC. Neurotoxicology.

[30] Guan Z., Liang Y., Zhu Z., Yang A., Li S., Guo J., Wang F., Yang H., Zhang N., Wang X. (2023). Cytosine Arabinoside Exposure Induced Cytotoxic Effects and Neural Tube Defects in Mice and Embryo Stem Cells. Ecotoxicol. Environ. Saf..

[31] Salimi A., Shabani M., Nikjou A., Choupani M., Biniyaz M. (2023). Exploring the Possible Mitoprotective and Neuroprotective Potency of Thymoquinone, Betanin, and Vitamin D against Cytarabine-Induced Mitochondrial Impairment and Neurotoxicity in Rats’ Brain. J. Biochem. Mol. Toxicol..

[32] Takano T., Akahori S., Takeuchi Y., Ohno M. (2006). Neuronal Apoptosis and Gray Matter Heterotopia in Microcephaly Produced by Cytosine Arabinoside in Mice. Brain Res..

[33] Yamauchi H., Katayama K.I., Ueno M., Uetsuka K., Nakayama H., Doi K. (2004). Involvement of P53 in 1-β-d-Arabinofuranosylcytosine-Induced Rat Fetal Brain Lesions. Neurotoxicol. Teratol..

[34] Shimada M., Wakaizumi S., Kasubuchi Y., Kusonoki T. (1975). Cytarabine and Its Effect on Cerebellum of Suckling Mouse. Arch. Neurol..

[35] Percy D.H., Albert D.M. (1974). Developmental Defects in Mice and Rats Treated Postnatally with Cytosine Arabinoside. Exp. Mol. Pathol..

[36] Percy D.H. (1975). Teratogenic Effects of the Pyrimidine Analogues 5-lododeoxyuridine and Cytosine Arabinoside in Late Fetal Mice and Rats. Teratology.

[37] Guzmán D.C., Brizuela N.O., Herrera M.O., Olguín H.J., Peraza A.V., Ruíz N.L., Mejía G.B. (2024). Intake of Oligoelements with Cytarabine or Etoposide Alters Dopamine Levels and Oxidative Damage in Rat Brain. Sci. Rep..

[38] Narang H.K. (1982). Pathological Findings of Adenine Arabinoside (Ara-A) and Cytarabine (Ara-C) in the Treatment of Herpes Simplex Encephalitis in Rabbit Model. Antivir. Res..

[39] Matsutani T., Tamaru M., Hayakawa Y., Nagayoshi M., Nakahara T., Tsukada Y. (1983). A Neurochemical Study of Developmental Impairment of the Brain Caused by the Administration of Cytosine Arabinoside during the Fetal or Neonatal Period of Rats. Neurochem. Res..

[40] Yamano T., Shimada M., Ohota S., Abe Y., Nakao K., Ohoya N. (1980). Formation of Heterotopic Granule Cell in Mouse Cerebellum after Neonatal Administration of Cytosine Arabinoside. Acta Neuropathol..

[41] Kasubuchi Y., Wakaizumi S., Shimada M., Kusunoki T. (1977). Cytosine Arabinoside-induced Transplacental Dysgenetic Hydrocephalus in Mice. Teratology.

[42] ADLARD B.P.F., DOBBING J., SANDS J. (1975). A Comparison of the Effects of Cytosine Arabinoside and Adenine Arabinoside on Some Aspects of Brain Growth and Development in the Rat. Br. J. Pharmacol..

[43] Elmer G.I., Sydnor J., Guard H., Hercher E., Vogel M.W. (2004). Altered Prepulse Inhibition in Rats Treated Prenatally with the Antimitotic Ara-C: An Animal Model for Sensorimotor Gating Deficits in Schizophrenia. Psychopharmacology.

[44] Yamano T., Shimada M., Abe Y., Ohta S., Ohno M. (1983). Destruction of External Granular Layer and Subsequent Cerebellar Abnormalities. Acta Neuropathol..

[45] de Souza Silva P.M., de Sousa R.V., Simão A.A., Cesar P.H.S., Trento M.V.C., Marcussi S. (2018). Protective Effect of β-D-Glucan and Glutamine on the Genomic Instability Induced by Cytarabine/Ara-C in BALB/c Mice. Int. J. Biol. Macromol..

[46] Li J.J., Li Y.L., Chu W., Li G.Q., Zhang M., Dong J.J., Li L., Li C.H., Zhang J.B., Li J.W. (2023). Astragaloside IV Alleviates Cytarabine-Induced Intestinal Mucositis by Remodeling Macrophage Polarization through AKT Signaling. Phytomedicine.

[47] Ramos M.G., Bambirra E.A., Cara D.C., Vieira E.C., Alvarez-Leite J.I. (1997). Oral Administration of Short-Chain Fatty Acids Reduces the Intestinal Mucositis Caused by Treatment with Ara-C in Mice Fed Commercial or Elemental Diets. Nutr. Cancer.

[48] Ramos M.G., Bambirra E.A., Nicoli J.R., Cara D.C., Vieira E.C., Alvarez-Leite J. (1999). Protection by Short-Chain Fatty Acids against 1-β-D-Arabinofuranosylcytosine-Induced Intestinal Lesions in Germfree Mice. Antimicrob. Agents Chemother..

[49] Chu W., Li Y.-L., Li J.-J., Lin J., Li M., Wang J., He J.-Z., Zhang Y.-M., Yao J., Jin X.-J. (2023). Guiqi Baizhu Prescription Ameliorates Cytarabine-Induced Intestinal Mucositis by Targeting JAK2 to Inhibit M1 Macrophage Polarization. Biomed. Pharmacother..

[50] Al-Jammas S., Al-Allaf L.I.K., Saeed M.G. (2024). Attenuating Effects of α-Tocopherol on Cytarabine-Induced Toxicity in Parotid Salivary Gland of Rabbits: A Histological and Immunohistochemical Study. Iran. J. Vet. Med..

[51] Minden M.D., Audiger C., Chabot-Roy G., Lesage S., Delisle J.S., Biemans B., Dimitriadou V. (2024). The Long-Acting Glucagon-Like Peptide-2 Analog Apraglutide Enhances Intestinal Protection and Survival After Chemotherapy and Allogeneic Transplantation in Mice. Ann. Transpl..

[52] Porsani M.Y.H., Paludetti M., Orlando D.R., Peconick A.P., Costa R.C., Oliveira L.E.D., Zangeronimo M.G., Sousa R.V. (2017). Protective Effect of β-Glucan and Glutamine on Intestinal and Immunological Damage in Mice Induced by Cytarabine (Ara-C). Pesqui. Veterinária Bras..

[53] Elli M., Aydin O., Bilge S., Bozkurt A., Dagdemir A., Pinarli F.G., Acar S. (2009). Protective Effect of Vitamin A on ARA-C Induced Intestinal Damage in Mice. Tumori.

[54] Chwalinski S., Potten C.S. (1989). Crypt Base Columnar Cells in Ileum of BDF1 Male Mice—Their Numbers and Some Features of Their Proliferation. Am. J. Anat..

[55] Han S., Xiu M., Li S., Shi Y., Wang X., Lin X., Cai H., Liu Y., He J. (2023). Exposure to Cytarabine Causes Side Effects on Adult Development and Physiology and Induces Intestinal Damage via Apoptosis in Drosophila. Biomed. Pharmacother..

[56] Chen T.S. (1982). Effects of Arabinosyl Cytosine and 5-Azacytidine on the Intestinal Absorption of Nutrients. Toxicol. Appl. Pharmacol..

[57] Liu R., Xue J., Han J., Tu M., Wang W., Chen Z., Qian X., Xiao B., Liang L. (2024). Cytarabine Chemotherapy Induces Meibomian Gland Dysfunction. Ocul. Surf..

[58] Balci Y.I., Acer S., Yagci R., Kucukatay V., Sarbay H., Bozkurt K., Polat A. (2017). N-Acetylcysteine Supplementation Reduces Oxidative Stress for Cytosine Arabinoside in Rat Model. Int. Ophthalmol..

[59] Rootman J., Gudauskas G., Kumi C. (1983). Subconjunctival versus Intravenous Cytosine Arabinoside: Effect of Route of Administration and Ocular Toxicity. Investig. Ophthalmol. Vis. Sci..

[60] Diets-Ouwehand J.J.A.T., de Keizer R.J.W., Vrensen G.F.J.M., Groen-Jansen S., van Best J.A. (1992). Toxicity of 1-(β-d-Arabinofuranosyl)Cytosine after Intravitreal Injection in the Rabbit Eye. Graefe’s Arch. Clin. Exp. Ophthalmol..

[61] Percy D.H., Danylchuk K.D. (1977). Experimental Retinal Dysplasia Due to Cytosine Arabinoside. Investig. Ophthalmol. Vis. Sci..

[62] Shimada M., Wakaizumi S., Kasubuchi Y., Kusunoki T., Nakamura T. (1973). Cytosine Arabinoside and Rosette Formation in Mouse Retina. Nature.

[63] Kaufman H.E., Capella J.A., Maloney E.D., Robbins J.E., Cooper G.M., Uotila M.H. (1964). Corneal Toxicity of Cytosine Arabinoside. Arch. Ophthalmol..

[64] Jimenez J.J., Huang H.S. (1992). IL-1 Synergizes with ARA-C in Aborting the Development of Chloroleukemia While Protecting from ARA-C-Induced Alopecia in the Rat Model. Cytokine.

[65] Jimenez J.J., Huang H.S., Yunis A.A. (1992). Treatment with Imuvert/JV-Acetylcysteine Protects Rats from Cyclophosphamide/Cytarabine-Induced Alopecia: Original Article. Cancer Investig..

[66] Jimenez J.J., Sawaya M.E., Yunis A.A. (1992). Interleukin 1 Protects Hair Follicles from Cytarabine (ARA-C)-Induced Toxicity in Vivo and in Vitro. FASEB J..

[67] Sun B., Wakame K., Sato E., Nishioka H., Aruoma O.I., Fujii H. (2009). The Effect of Active Hexose Correlated Compound in Modulating Cytosine Arabinoside-Induced Hair Loss, and 6-Mercaptopurine- and Methotrexate-Induced Liver Injury in Rodents. Cancer Epidemiol..

[68] Hussein A.M. (1995). Protection Against Cytosine Arabinoside-Induced Alopecia By Minoxidil in a Rat Animal Model. Int. J. Dermatol..

[69] Hagiwara S., Uchida T., Koga H., Inomata M., Yoshizumi F., Moriyama M., Kitano S., Noguchi T. (2011). The α-Lipoic Acid Derivative Sodium Zinc Dihydrolipoylhistidinate Reduces Chemotherapy-Induced Alopecia in a Rat Model: A Pilot Study. Surg. Today.

[70] Sun F., Li N., Tong X., Zeng J., He S., Gai T., Bai Y., Liu L., Lu K., Shen J. (2019). Ara-c Induces Cell Cycle G1/S Arrest by Inducing Upregulation of the INK4 Family Gene or Directly Inhibiting the Formation of the Cell Cycle-Dependent Complex CDK4/Cyclin D1. Cell Cycle.

[71] Kolure R., Nachammai V., Manjula S.N., Godela R., Bhavani D.S., Rajendra Y. (2023). Hepatoprotective Effect of Swertiamarin from Cytarabine Induced Hepatotoxicity in Pregnant Rats. Res. J. Pharm. Technol..

[72] Al-Jammas S., Al-Saraj A. (2020). The Histological Changes Induced by Cytarabine on Rabbits Livers (with and without Vitamin E Administration). Iraqi J. Vet. Sci..

[73] Dudina M.O., Suslova I.R., Khalzova M.S., Dergunova J.V., Kogan E.A., Roshchin D.A., Samyshina E.A., Morozov M.A., Dydykin S.S. (2018). Molecular and Cellular Mechanisms of Acute Cytotoxic Liver Damage as Potential Biological Targets for Magnesium-Containing Cell-Protective Drug. Res. Results Pharmacol..

[74] Palo A.K., Sahoo D., Choudhury R.C. (2009). Cytosine Arabinoside-Induced Cytogenotoxicity in Bone Marrow and Spermatogonial Cells of Mice and Its Potential Transmission through the Male Germline. Mutat. Res..

[75] Lee J.Y., Han A.R., Hwang H.-S., Kim D.C., Min W.S., Kim H.J. (2018). Role of CXCR4 Antagonist in Megakaryocyte Reinstatement with Increased Sinusoidal Vessel Density. Oxygen Transport to Tissue XL.

[76] Castañeda-Yslas I.Y., Torres-Bugarín O., Arellano-García M.E., Ruiz-Ruiz B., García-Ramos J.C., Toledano-Magaña Y., Pestryakov A., Bogdanchikova N. (2024). Protective Effect of Silver Nanoparticles Against Cytosine Arabinoside Genotoxicity: An In Vivo Micronucleus Assay. Int. J. Environ. Res. Public Health.

[77] Zhu R.J., Wu M.Q., Li Z.J., Zhang Y., Liu K.Y. (2013). Hematopoietic Recovery Following Chemotherapy Is Improved by BADGE-Induced Inhibition of Adipogenesis. Int. J. Hematol..

[78] Wang J., Zheng M., Min Q., Gao Y., Sun W. (2018). The Dual Regulatory Function of Lienal Peptide on Immune System. Int. Immunopharmacol..

[79] Bilgin A.O., Mammadov R., Suleyman B., Unver E., Ozcicek F., Soyturk M., Cimen F.K., Kurt N., Suleyman H. (2020). Effect of Rutin on Cytarabine-Associated Pulmonary Oedema and Oxidative Stress in Rats. Acad. Bras. Cienc..

[80] Namoju R., Chilaka N.K. (2021). Alpha-Lipoic Acid Ameliorates Cytarabine-Induced Developmental Anomalies in Rat Fetus. Hum. Exp. Toxicol..

[81] Zhao X., Yang W., Li G., Dong H., Hou J., Cao Z., Guan D. (2020). Expression of Fibroblast Growth Factor 4 in a Rat Model of Polydactyly of the Thumb Induced by Cytarabine. Med. Sci. Monit..

[82] Chilaka K.N., Namoju R. (2024). Maternal Supplementation of Alpha-Lipoic Acid Ameliorates Prenatal Cytarabine-Induced Mutilation in Reproductive Development and Function in F1 Male Adult Rats. Naunyn Schmiedebergs Arch. Pharmacol..

[83] Yamauchi H., Katayama K., Yasoshima A., Uetsuka K., Nakayama H., Doi K. (2003). 1-β-D-Arabinofuranosylcytosine (Ara-C)-Induced Apoptosis in the Rat Fetal Tissues and Placenta. J. Toxicol. Pathol..

[84] Kochhar D.M., Penner J.D., McDay J.A. (1978). Limb Development in Mouse Embryos. II. Reduction Defects, Cytotoxicity and Inhibition of DNA Synthesis Produced by Cytosine Arabinoside. Teratology.

[85] Manson J.M., Dourson M.L., Smith C.C. (1977). Effects of Cytosine Arabinoside on in Vivo and in Vitro Mouse Limb Development. Vitr. J. Tissue Cult. Assoc..

[86] Rahman M.E., Ishikawa H., Watanabe Y., Endo A. (1994). Carpal and Tarsal Bone Anomalies in Mice Induced by Maternal Treatment of Ara-C. Reprod. Toxicol..

[87] Rahman M.E., Ishikawa H., Watanabe Y., Endo A. (1996). Carpal and Tarsal Bone Development Is Highly Sensitive to Three Antiproliferative Teratogens in Mice. Reprod. Toxicol..

[88] Chiang H., Wu R.Y., Shao B.J., Fu Y.D., Yao G.D., Lu D.J. (1995). Pulsed Magnetic Field from Video Display Terminals Enhances Teratogenic Effects of Cytosine Arabinoside in Mice. Bioelectromagnetics.

[89] Chiba K., Ishikawa H., Rahman M.E., Endo A. (1996). Neonatal Mouse Hip Joint and Hindlimb Anomalies Induced by Prenatal Exposure to Ara-C. Reprod. Toxicol..

[90] Ritter E.J., Scott W.J., Wilson J.G. (1971). Teratogenesis and Inhibition of DNA Synthesis Induced in Rat Embryos by Cytosine Arabinoside. Teratology.

[91] Ritter E.J., Scott W.J., Wilson J.G. (1973). Relationship of Temporal Patterns of Cell Death and Development to Malformations in the Rat Limb. Possible Mechanisms of Teratogenesis with Inhibitors of DNA Synthesis. Teratology.

[92] Scott W.J., Ritter E.J., Wilson J.G. (1975). Studies on Induction of Polydactyly in Rats with Cytosine Arabinoside. Dev. Biol..

[93] Goto T., Endo A. (1987). Dose- and Stage-related Sex Difference in the Incidence of Cytosine Arabinoside Induced Digit Anomalies in the Mouse Fetus. Teratology.

[94] Endo A., Sakai N., Ohwada K. (1987). Analysis of Diurnal Difference in Teratogen (Ara-C) Susceptibility in Mouse Embryos by a Progressive Phase-shift Method. Teratog. Carcinog. Mutagen..

[95] Rahman M.E., Ishikawa H., Watanabe Y., Endo A. (1995). Stage Specificity of Ara-C Induced Carpal and Tarsal Bone Anomalies in Mice. Reprod. Toxicol..

[96] Chaube S., Kreis W., Uchida K., Murphy M.L. (1968). The Teratogenic Effect of 1-β-d-Arabinofuranosylcytosine in the Rat: Protection by Deoxycytidine. Biochem. Pharmacol..

[97] Nair A., Jacob S. (2016). A Simple Practice Guide for Dose Conversion between Animals and Human. J. Basic Clin. Pharm..

[98] Damon L.E., Mass R., Linker C.A. (1989). The Association between High-Dose Cytarabine Neurotoxicity and Renal Insufficiency. J. Clin. Oncol..

[99] Salinsky M.C., Levine R.L., Aubuchon J.P., Schutta H.S. (1983). Acute Cerebellar Dysfunction with High-Dose ARA-C Therapy. Cancer.

[100] Phillips N.S., Stratton K.L., Williams A.L.M., Ahles T., Ness K.K., Cohen H.J., Edelstein K., Yasui Y., Oeffinger K., Chow E.J. (2023). Late-Onset Cognitive Impairment and Modifiable Risk Factors in Adult Childhood Cancer Survivors. JAMA Netw. Open.

[101] Yamauchi H., Katayama K.I., Ueno M., Uetsuka K., Nakayama H., Doi K. (2004). Involvement of P53 in 1-β-d-Arabinofuranosylcytosine-Induced Trophoblastic Cell Apoptosis and Impaired Proliferation in Rat Placenta. Biol. Reprod..

[102] Camera A., Andretta C., Villa M.R., Volpicelli M., Picardi M., Rossi M., Rinaldi C.R., Cioppa P.D., Ciancia R., Selleri C. (2003). Intestinal Toxicity during Induction Chemotherapy with Cytarabine-Based Regimens in Adult Acute Myeloid Leukemia. Hematol. J..

[103] Slavin R.E., Dias M.A., Saral R. (1978). Cytosine Arabinoside Induced Gastrointestinal Toxic Alterations in Sequential Chemotherapeutic Protocols.A Clinical-Pathologic Study of 33 Patients. Cancer.

[104] Park M.R., Lee H.J., Jang H.M., Kim N.H., Lee J.S., Jeong Y.T., Kim I., Choi S.H., Seo K.S., Kim D.H. (2023). Cytarabine Induces Cachexia with Lipid Malabsorption via Zippering the Junctions of Lacteal in Murine Small Intestine. J. Lipid Res..

[105] Dekker I.M., Bruggink H., van der Meij B.S., Wierdsma N.J. (2021). State of the Art: The Role of Citrulline as Biomarker in Patients with Chemotherapy- or Graft-versus-Host-Disease-Induced Mucositis. Curr. Opin. Clin. Nutr. Metab. Care.

[106] Higa G.M., Gockerman J.P., Hunt A.L., Jones M.R., Horne B.J. (1991). The Use of Prophylactic Eye Drops during High-Dose Cytosine Arabinoside Therapy. Cancer.

[107] Guthoff T., Tietze B., Meinhardt B., Becher J., Guthoff R. (2010). Cytosine-Arabinoside-Induced Keratopathy: A Model of Corneal Proliferation Kinetics. Ophthalmologica.

[108] Jimenez-Cauhe J., Lo Sicco K.I., Shapiro J., Hermosa-Gelbard A., Burgos-Blasco P., Melian-Olivera A., Ortega-Quijano D., Pindado-Ortega C., Buendia-Castaño D., Asz-Sigall D. (2025). Characterization and Management of Adverse Events of Low-Dose Oral Minoxidil Treatment for Alopecia: A Narrative Review. J. Clin. Med..

[109] Kearney C.A., Gordon A., Markova A., Freites-Martinez A., Tattersall I.W., Shapiro J., Lacouture M.E., Lo Sicco K.I. (2025). Low-Dose Oral Minoxidil during Chemotherapy: A Review of the Mechanism and Current Evidence. Ski. Appendage Disord..

[110] Hospira, Inc. (2022). Cytarabine Injection, Solution for Injection, 20 Mg/ML.

[111] Choi E.K., Kim I.-R., Chang O., Kang D., Nam S.-J., Lee J.E., Lee S.K., Im Y.-H., Park Y.H., Yang J.-H. (2014). Impact of Chemotherapy-Induced Alopecia Distress on Body Image, Psychosocial Well-Being, and Depression in Breast Cancer Patients. Psychooncology.

[112] Chiche E., Rahmé R., Bertoli S., Dumas P.-Y., Micol J.-B., Hicheri Y., Pasquier F., Peterlin P., Chevallier P., Thomas X. (2021). Real-Life Experience with CPX-351 and Impact on the Outcome of High-Risk AML Patients: A Multicentric French Cohort. Blood Adv..

[113] National Institute of Diabetes and Digestive and Kidney Diseases (2017). Cytarabine. LiverTox: Clinical and Research Information on Drug-Induced Liver Injury.

[114] Fu S.H., Flannery A.H., Thompson Bastin M.L. (2019). Acute Hepatotoxicity After High-Dose Cytarabine for the Treatment of Relapsed Acute Myeloid Leukemia: A Case Report. Hosp. Pharm..

[115] Laharwal M.M., Law C.S., Orosz E., Patel A.V. (2020). S2680 Isolated Direct Hyperbilirubinemia Due to Cytarabine Administration. Am. J. Gastroenterol..

[116] da Lyrio R.M.C., Rocha B.R.A., Corrêa A.L.R.M., Mascarenhas M.G.S., Santos F.L., da Maia R.H., Segundo L.B., de Almeida P.A.A., Moreira C.M.O., Sassi R.H. (2024). Chemotherapy-Induced Acute Kidney Injury: Epidemiology, Pathophysiology, and Therapeutic Approaches. Front. Nephrol..

[117] Tanaka M., Kanamori H., Yamaji S., Mishima A., Fujita H., Fujisawa S., Murata T., Koharazawa H., Matsuzaki M., Mohri H. (1999). Low-Dose Cytarabine-Induced Hepatic and Renal Dysfunction in a Patient with Myelodysplastic Syndrome. Anticancer Drugs.

[118] Khaleel B., Lunenfeld E., Kapelushnik J., Huleihel M. (2023). Effect of Granulocyte Colony-Stimulating Factor on the Development of Spermatogenesis in the Adulthood of Juvenile AML Mice Model Treated with Cytarabine. Int. J. Mol. Sci..

[119] Orth J.M., Gunsalus G.L., Lamperti A.A. (1988). Evidence from Sertoli Cell-Depleted Rats Indicates That Spermatid Number in Adults Depends on Numbers of Sertoli Cells Produced during Perinatal Development. Endocrinology.

[120] Watanabe S., Sato S., Nagase S., Saito T. (1992). A Novel Note on the Effect of Ara-C on the Polyamine Content of the Male Accessory Organs of the Rat. Biochem. Pharmacol..

[121] Cano F., Pannel R., Follows G.A., Rabbitts T.H. (2008). Preclinical Modeling of Cytosine Arabinoside Response in Mll-Enl Translocator Mouse Leukemias. Mol. Cancer Ther..

