# Supplementary material for: Unraveling Translational Insights into Systemic Multi-Organ Toxicity of Cytosine Arabinoside (Ara-C): A Systematic Review of Preclinical Animal Evidence
Source: Curr Issues Mol Biol. 2025 Dec 19;48(1):4. doi: 10.3390/cimb48010004 (PMC12840079; doi:10.3390/cimb48010004)
Supplement: Supplementary file 1 [file cimb-48-00004-s001.zip › cimb-4028198-supplementary.pdf]

current issues in molecular biology

Supplementary Table S1: PRISMA 2020 checklist

Section and Topic	Item #	Checklist item	Location where item is reported
**TITLE**			
Title	1	Identify the report as a systematic review.	1
**ABSTRACT**			
Abstract	2	See the PRISMA 2020 for Abstracts checklist.	1
**INTRODUCTION**			
Rationale	3	Describe the rationale for the review in the context of existing knowledge.	2
Objectives	4	Provide an explicit statement of the objective(s) or question(s) the review addresses.	2
**METHODS**			
Eligibility criteria	5	Specify the inclusion and exclusion criteria for the review and how studies were grouped for the syntheses.	3
Information sources	6	Specify all databases, registers, websites, organisations, reference lists and other sources searched or consulted to identify studies. Specify the date when each source was last searched or consulted.	3
Search strategy	7	Present the full search strategies for all databases, registers and websites, including any filters and limits used.	3
Selection process	8	Specify the methods used to decide whether a study met the inclusion criteria of the review, including how many reviewers screened each record and each report retrieved, whether they worked independently, and if applicable, details of automation tools used in the process.	3
Data collection process	9	Specify the methods used to collect data from reports, including how many reviewers collected data from each report, whether they worked independently, any processes for obtaining or confirming data from study investigators, and if applicable, details of automation tools used in the process.	3-4
Data items	10a	List and define all outcomes for which data were sought. Specify whether all results that were compatible with each outcome domain in	3-4

 https://doi.org/10.3390/cimb48010004

Section and Topic	Item #	Checklist item	Location where item is reported
		each study were sought (e.g. for all measures, time points, analyses), and if not, the methods used to decide which results to collect.	
	10b	List and define all other variables for which data were sought (e.g. participant and intervention characteristics, funding sources). Describe any assumptions made about any missing or unclear information.	3-4
Study risk of bias assessment	11	Specify the methods used to assess risk of bias in the included studies, including details of the tool(s) used, how many reviewers assessed each study and whether they worked independently, and if applicable, details of automation tools used in the process.	4
Effect measures	12	Specify for each outcome the effect measure(s) (e.g. risk ratio, mean difference) used in the synthesis or presentation of results.	N/A
Synthesis methods	13a	Describe the processes used to decide which studies were eligible for each synthesis (e.g. tabulating the study intervention characteristics and comparing against the planned groups for each synthesis (item #5)).	4
	13b	Describe any methods required to prepare the data for presentation or synthesis, such as handling of missing summary statistics, or data conversions.	4
	13c	Describe any methods used to tabulate or visually display results of individual studies and syntheses.	4
	13d	Describe any methods used to synthesize results and provide a rationale for the choice(s). If meta-analysis was performed, describe the model(s), method(s) to identify the presence and extent of statistical heterogeneity, and software package(s) used.	44
	13e	Describe any methods used to explore possible causes of heterogeneity among study results (e.g. subgroup analysis, meta-regression).	
	13f	Describe any sensitivity analyses conducted to assess robustness of the synthesized results.	4
Reporting bias assessment	14	Describe any methods used to assess risk of bias due to missing results in a synthesis (arising from reporting biases).	4
Certainty assessment	15	Describe any methods used to assess certainty (or confidence) in the body of evidence for an outcome.	4
**RESULTS**			
Study selection	16a	Describe the results of the search and selection process, from the number of records identified in the search to the number of studies	4-5

https://doi.org/10.3390/cimb48010004

Section and Topic	Item #	Checklist item	Location where item is reported
		included in the review, ideally using a flow diagram.	
	16b	Cite studies that might appear to meet the inclusion criteria, but which were excluded, and explain why they were excluded.	4-5
Study characteristics	17	Cite each included study and present its characteristics.	5
Risk of bias in studies	18	Present assessments of risk of bias for each included study.	7
Results of individual studies	19	For all outcomes, present, for each study: (a) summary statistics for each group (where appropriate) and (b) an effect estimate and its precision (e.g. confidence/credible interval), ideally using structured tables or plots.	7 & Tables 2-10 and S6
Results of syntheses	20a	For each synthesis, briefly summarise the characteristics and risk of bias among contributing studies.	7 & Tables 2-10
	20b	Present results of all statistical syntheses conducted. If meta-analysis was done, present for each the summary estimate and its precision (e.g. confidence/credible interval) and measures of statistical heterogeneity. If comparing groups, describe the direction of the effect.	N/A
	20c	Present results of all investigations of possible causes of heterogeneity among study results.	N/A
	20d	Present results of all sensitivity analyses conducted to assess the robustness of the synthesized results.	N/A
Reporting biases	21	Present assessments of risk of bias due to missing results (arising from reporting biases) for each synthesis assessed.	7 & Table 2
Certainty of evidence	22	Present assessments of certainty (or confidence) in the body of evidence for each outcome assessed.	7 & Table 2
**DISCUSSION**			
Discussion	23a	Provide a general interpretation of the results in the context of other evidence.	11
	23b	Discuss any limitations of the evidence included in the review.	23-24

https://doi.org/10.3390/cimb48010004

Section and Topic	Item #	Checklist item	Location where item is reported
	23c	Discuss any limitations of the review processes used.	23-24
	23d	Discuss implications of the results for practice, policy, and future research.	24
**OTHER INFORMATION**			
Registration and protocol	24a	Provide registration information for the review, including register name and registration number, or state that the review was not registered.	3
	24b	Indicate where the review protocol can be accessed, or state that a protocol was not prepared.	3
	24c	Describe and explain any amendments to information provided at registration or in the protocol.	N/A
Support	25	Describe sources of financial or non-financial support for the review, and the role of the funders or sponsors in the review.	25
Competing interests	26	Declare any competing interests of review authors.	25
Availability of data, code and other materials	27	Report which of the following are publicly available and where they can be found: template data collection forms; data extracted from included studies; data used for all analyses; analytic code; any other materials used in the review.	25

Supplementary Table S2: SYRCLE's risk of bias tool.

Author, Year	1. Sequence generation (Randomization)	2. Baseline characteristics	3. Allocation concealment	4. Random housing	5. Blinding of caregivers & investigators	6. Random outcome assessment	7. Blinding of outcome assessor	8. Incomplete outcome data	9. Selective outcome reporting	10. Other sources of bias	Overall SYRCLE judgment for RoB

https://doi.org/10.3390/cimb48010004

Ren Liu et al., 2024	Yes	Yes	U	U	No	Yes	Yes	Yes	Yes	Yes	Low
Alexander TC et al, 2018	No	Yes	U	U	No	U	Partial	Yes	Yes	U	High
Namoju R et al, 2021	No	Yes	U	U	No	U	No	Yes	Yes	Yes	Moderate
de Souza Silva PM et al, 2018	Yes	Yes	U	U	No	Yes	No	Yes	Yes	Yes	Low
Zhao X et al, 2020	No	Yes	U	U	No	U	No	Yes	Yes	Yes	Moderate
Balci YI et al, 2016	Yes	Yes	U	U	No	Partial	Yes	Yes	Yes	Yes	Low
Fremouw T et al, 2012	No	Yes	U	U	No	U	Yes	Yes	Yes	Yes	Moderate
Jimenez JJ et al,1992	No	Yes	U	U	No	U	No	Yes	Yes	Yes	Moderate
Jimenez JJ et al,1992	No	Yes	U	U	No	U	No	Yes	Yes	Yes	Moderate
Shuzhen Han et al., 2023	No	Yes	U	U	No	U	No	Yes	Yes	Yes	Moderate
Sun F et al, 2019	No	Yes	U	U	No	U	No	Yes	Yes	Yes	Moderate
**Li JJ et al, 2023**	Yes	Yes	U	U	No	Yes	Yes	Yes	Yes	Yes	Low
Li CQ et al, 2008	Yes	Yes	U	U	No	Yes	Yes	Yes	Yes	Yes	Low
Palo AK et al, 2009	Yes	Yes	U	U	No	Yes	No	Yes	Yes	Yes	Moderate
Rootman J et al, 1983	No	Yes	U	U	No	U	No	Yes	Yes	U	High
Lee JY et al,2018	No	Yes	U	U	No	U	No	Yes	Yes	U	High
Ramos MG et al, 1997	No	Yes	U	U	No	Yes	Yes	Yes	Yes	U	Moderate
Castañeda-Yslas IY et al, 2024	No	Yes	U	U	No	U	U	Yes	Yes	Yes	Moderate
Chilaka KN et al, 2024	Yes	Yes	U	U	No	Yes	No	Yes	Yes	Yes	Moderate

https://doi.org/10.3390/cimb48010004

Bilgin AO et al, 2020	No	Yes	U	U	No	U	No	Yes	Yes	U	High
Chu W et al, 2023	Yes	Yes	U	U	No	Yes	Yes	Yes	Yes	Yes	Low
Guzmán DC et al, 2018	No	Yes	U	U	No	U	No	Yes	Yes	U	High
Guzmán DC et al, 2016	Yes	Yes	U	U	No	U	No	Yes	Yes	Yes	Moderate
Zhu RJ et al, 2013	No	Yes	U	U	No	U	No	Yes	Yes	Yes	Moderate
Patel RS et al, 2012	Yes	Yes	U	U	No	U	No	Yes	Yes	Yes	Moderate
Koros C et al, 2007	Yes	Yes	U	U	No	Yes	Yes	Yes	Yes	Yes	Low
Koros C et al, 2009	Yes	Yes	U	U	No	Yes	Yes	Yes	Yes	Yes	Low
Saif A-J et al, 2024	No	Yes	U	U	No	U	No	Yes	Yes	Yes	Moderate
Minden MD et al, 2024	Yes	Yes	U	U	No	U	No	Yes	Yes	Yes	Moderate
Park M-R et al, 2023	No	Yes	U	U	No	U	No	Yes	Yes	Yes	Moderate
Kolure R et al, 2023	Yes	Yes	U	U	No	U	No	Yes	Yes	U	Moderate
Guan Z et al, 2023	Yes	Yes	U	U	No	Yes	No	Yes	Yes	Yes	Moderate
Salimi A et al, 2023	No	Yes	U	U	No	U	No	Yes	Yes	Yes	Moderate
Khaleel B et al, 2022	No	Yes	U	U	No	U	No	Yes	Yes	Yes	Moderate
Saif A-J et al, 2020	No	Yes	U	U	No	U	No	Yes	Yes	U	High
Dudina MO et al, 2018	No	Yes	U	U	No	U	No	Yes	Yes	U	High
Porsani MYH et al, 2017	No	Yes	U	U	No	Yes	No	Yes	Yes	Yes	Moderate
Takano T et al, 2006	No	Yes	U	U	No	U	No	Yes	Yes	U	High
Elli M et al, 2009	No	Yes	U	U	No	U	Yes	Yes	Yes	U	Moderate
Sun B et al ,2009	Partial	Yes	U	U	No	U	No	Yes	Yes	U	High

Diets-Ouwehand JJAT et al, 1992	No	Yes	U	U	No	U	No	Yes	Yes	U	High
Watanabe S et al, 1992	No	Yes	U	U	No	U	No	Yes	Yes	U	High
Yamauchi H et al, 2004	No	Yes	U	U	No	Yes	No	Yes	Yes	U	Moderate
Percy DH et al, 1977	No	Yes	U	U	No	U	No	Yes	Yes	U	High
Shimada M et al, 1975	No	Yes	U	U	No	U	No	Yes	Yes	U	High
Ramos MG et al, 1999	No	No	U	U	No	U	No	Yes	Yes	U	High
Hussein AM, 1995	No	Yes	U	U	No	U	No	Yes	Yes	U	High
Percy DH et al, 1974	No	Yes	U	U	No	U	No	Yes	Yes	U	High
Yamano T et al, 1983	No	Yes	U	U	No	U	No	Yes	Yes	U	High
Percy DH, 1975	No	Yes	U	U	No	U	No	Yes	Yes	U	High
Shimada M et al, 1973	No	Yes	U	U	No	U	No	Yes	Yes	U	High
Guzmán DC et al, 2024	Yes	Yes	U	U	No	U	No	Yes	Yes	Yes	Moderate
Narang HK, 1982	No	Yes	U	U	No	U	No	Yes	Yes	U	High
Yamauchi H et al, 2003	No	Yes	U	U	No	Yes	No	Yes	Yes	U	Moderate
Matsutani T et al, 1983	No	Yes	U	U	No	U	No	Yes	Yes	U	High
Chwalinski S et al, 1989	No	Yes	U	U	No	U	No	Yes	Yes	U	High

Kaufman HE et al, 1964	No	Yes	U	U	No	U	Yes	Yes	Yes	U	Moderate
Yamano T et al, 1980	No	Yes	U	U	No	U	No	Yes	Yes	U	High
Hagiwara S et al, 2011	No	Yes	U	U	No	U	Yes	Yes	Yes	U	Moderate
Orth JM et al, 1988	No	Yes	U	U	No	No	No	Yes	Yes	U	High
Wang J et al, 2018	No	Yes	U	U	No	U	No	Yes	Yes	Yes	Moderate
Han S et al, 2023	No	Yes	U	U	No	U	No	Yes	Yes	Yes	Moderate
Chen T, 1982	No	Yes	U	U	No	U	No	Yes	Yes	U	High
Yamauchi H et al, 2004	No	Yes	U	U	No	U	No	Yes	Yes	U	High
Kochhar DM et al, 1978	No	Yes	U	U	No	U	No	Yes	Yes	U	High
Manson JM et al, 1977	No	Yes	U	U	No	U	No	Yes	Yes	U	High
Rahman ME et al, 1994	No	Yes	U	U	No	U	No	Yes	Yes	U	High
Rahman ME et al, 1996	No	Yes	U	U	No	U	No	Yes	Yes	U	High
Chiang H et al, 1995	Yes	Yes	U	U	No	U	No	Yes	Yes	U	Moderate
Chiba K et al, 1996	No	Yes	U	U	No	U	No	Yes	Yes	U	High
Ritter EJ et al, 1971	No	Yes	U	U	No	U	No	Yes	Yes	U	High
Kasubuchi Y et al, 1977	No	Yes	U	U	No	U	No	Yes	Yes	U	High
Adlard BP et al, 1975	No	Yes	U	U	No	U	No	Yes	Yes	U	High
Elmer GI et al, 2004	No	Yes	U	U	No	Yes	No	Yes	Yes	U	Moderate

Cano F et al, 2008	No	Yes	U	U	No	U	No	Yes	Yes	U	High
Ritter EJ et al, 1973	No	Yes	U	U	No	U	No	Yes	Yes	U	High
Scott WJ et al, 1975	No	Yes	U	U	No	U	No	Yes	Yes	U	High
Goto T et al, 1987	No	Yes	U	U	No	U	No	Yes	Yes	U	High
Endo A et al, 1987	No	Yes	U	U	No	U	No	Yes	Yes	U	High
Rahman ME et al, 1995	No	Yes	U	U	No	U	No	Yes	Yes	U	High
Chaube S et al, 1968	No	Yes	U	U	No	U	No	Yes	Yes	U	High

Supplementary Table S3: CAMARADES 10-point checklist.

Author, Year	1. Publication after peer review	2. Statement of control of temperature	3. Randomization to treatment or control	4. Blinded induction of experimental model	5. Blinded assessment of outcomes	6. Anesthetic without marked intrinsic neuroprotective activity	7. Appropriate animal model	8. Sample-size calculation	9. Statement of compliance with animal welfare regulations	10. Declared any potential conflict of interest	CAMARADES total score (out of 10)
Ren Liu et al., 2024	Yes	No	Yes	No	Yes	Yes	Yes	No	Yes	Yes	7
Alexander TC et al, 2018	Yes	No	No	No	Partial	Partial	Yes	No	Yes	No	4
Namoju R et al, 2021	Yes	Yes	No	No	No	Yes	Yes	No	Yes	Yes	6
de Souza Silva PM et al, 2018	Yes	Yes	Yes	No	No	Yes	Yes	No	Yes	Yes	7
Zhao X et al, 2020	Yes	Yes	No	No	No	Yes	Yes	No	Yes	Yes	6
Balci YI et al, 2017	Yes	Yes	Yes	No	Yes	Yes	Yes	No	Yes	Yes	8
Fremouw T et al, 2012	Yes	No	No	No	Yes	Yes	Yes	No	Yes	Yes	6
Jimenez JJ et al,1992	Yes	No	No	No	No	Yes	Yes	No	No	No	3
Jimenez JJ et al,1992	Yes	No	No	No	No	Yes	Yes	No	No	No	3
Shuzhen Han et al., 2023	Yes	Yes	No	No	No	Yes	Yes	No	Yes	Yes	6

https://doi.org/10.3390/cimb48010004

Sun F et al, 2019	Yes	No	No	No	No	Yes	Yes	No	Yes	Yes	5
**Li JJ et al, 2023**	Yes	No	Yes	No	Yes	Yes	Yes	No	Yes	Yes	7
Li CQ et al, 2008	Yes	Yes	Yes	No	Yes	Yes	Yes	No	Yes	Yes	8
Palo AK et al, 2009	Yes	Yes	Yes	No	Partial	Yes	Yes	No	Yes	Yes	7.5
Rootman J et al, 1983	Yes	No	No	No	No	Yes	Yes	No	No	No	3
Lee JY et al,2018	Yes	No	No	No	No	Yes	Yes	No	Yes	No	4
Ramos MG et al, 1997	Yes	No	No	No	Yes	Yes	Yes	No	No	No	4
Castañeda-Yslas IY et al, 2024	Yes	No	No	No	U	Yes	Yes	No	Yes	Yes	5
Chilaka KN et al, 2024	Yes	Yes	Yes	No	Yes	Yes	Yes	No	Yes	Yes	8
Bilgin AO et al, 2020	Yes	Yes	No	No	No	Yes	Yes	No	Yes	No	5
Chu W et al, 2023	Yes	Yes	Yes	No	Yes	Yes	Yes	No	Yes	Yes	8
Guzmán DC et al, 2018	Yes	Yes	No	No	No	Yes	Yes	No	Yes	No	5
Guzmán DC et al, 2016	Yes	No	Yes	No	No	Yes	Yes	No	Yes	Yes	6
Zhu RJ et al, 2013	Yes	Yes	No	No	No	Yes	Yes	No	Yes	Yes	6
Patel RS et al, 2012	Yes	Yes	Yes	No	No	Yes	Yes	No	Yes	Yes	7
Koros C et al, 2007	Yes	No	Yes	No	Yes	Yes	Yes	No	Yes	Yes	7
Koros C et al, 2009	Yes	No	Yes	No	Yes	Yes	Yes	No	Yes	Yes	7
Saif A-J et al, 2024	Yes	Yes	No	No	No	Yes	Yes	No	Yes	Yes	6
Minden MD et al, 2024	Yes	Yes	Yes	No	No	Yes	Yes	No	Yes	Yes	7
Park M-R et al, 2023	Yes	Yes	No	No	No	Yes	Yes	No	Yes	Yes	6
Kolure R et al, 2023	Yes	No	Yes	No	No	Yes	Yes	No	No	Yes	5
Guan Z et al, 2023	Yes	Yes	Yes	No	Yes	Yes	Yes	No	Yes	Yes	8
Salimi A et al, 2023	Yes	Yes	No	No	No	Yes	Yes	No	Yes	Yes	6
Khaleel B et al, 2022	Yes	No	No	No	No	Yes	Yes	No	Yes	Yes	5
Saif A-J et al, 2020	Yes	Yes	No	No	No	Yes	Yes	No	No	Yes	5
Dudina MO et al, 2018	Yes	No	No	No	No	Yes	Yes	No	Yes	No	4
Porsani MYH et al, 2017	Yes	Yes	No	No	Partial	Yes	Yes	No	Yes	Yes	6.5

Takano T et al, 2006	Yes	No	No	No	No	Yes	Yes	No	Yes	No	4
Elli M et al, 2009	Yes	Yes	No	No	Yes	Yes	Yes	No	Yes	No	6
Sun B et al ,2009	Yes	Yes	Partial	No	No	Yes	Yes	No	No	Yes	5.5
Diets-Ouwehand JJAT et al, 1992	Yes	No	No	No	No	Yes	Yes	No	No	No	3
Watanabe S et al, 1992	Yes	No	No	No	No	Yes	Yes	No	No	No	3
Yamauchi H et al, 2004	Yes	Yes	No	No	Partial	Yes	Yes	No	Yes	No	5.5
Percy DH et al, 1977	Yes	Yes	No	No	No	Yes	Yes	No	No	No	4
Shimada M et al, 1975	Yes	No	No	No	No	Yes	Yes	No	No	No	3
Ramos MG et al, 1999	Yes	No	No	No	No	Yes	Yes	No	No	No	3
Hussein AM, 1995	Yes	No	No	No	No	Yes	Yes	No	Yes	No	4
Percy DH et al, 1974	Yes	No	No	No	No	Yes	Yes	No	No	No	3
Yamano T et al, 1983	Yes	No	No	No	No	Yes	Yes	No	No	No	3
Percy DH, 1975	Yes	No	No	No	No	Yes	Yes	No	No	No	3
Shimada M et al, 1973	Yes	No	No	No	No	Yes	Yes	No	No	No	3
Guzmán DC et al, 2024	Yes	Yes	Yes	No	No	Yes	Yes	No	Yes	Yes	7
Narang HK, 1982	Yes	No	No	No	No	Yes	Yes	No	No	No	3
Yamauchi H et al, 2003	Yes	Yes	No	No	Yes	Yes	Yes	No	Yes	No	6
Matsutani T et al, 1983	Yes	No	No	No	No	Yes	Yes	No	No	No	3
Chwalinski S et al, 1989	Yes	No	No	No	No	Yes	Yes	No	No	No	3
Kaufman HE et al, 1964	Yes	No	No	No	Yes	Yes	Yes	No	No	No	4
Yamano T et al, 1980	Yes	No	No	No	No	Yes	Yes	No	No	No	3
Hagiwara S et al, 2011	Yes	No	No	No	Yes	Yes	Yes	No	Yes	No	5
Orth JM et al, 1988	Yes	No	No	No	No	Yes	Yes	No	No	No	3
Wang J et al, 2018	Yes	Yes	No	No	No	Yes	Yes	No	Yes	Yes	6
Han S et al, 2023	Yes	Yes	No	No	No	Yes	Yes	No	Yes	Yes	6
Chen T, 1982	Yes	No	No	No	No	Yes	Yes	No	No	No	3
Yamauchi H et al, 2004	Yes	Yes	No	No	No	Yes	Yes	No	Yes	No	5

https://doi.org/10.3390/cimb48010004

Kochhar DM et al, 1978	Yes	Yes	No	No	No	Yes	Yes	No	No	No	4
Manson JM et al, 1977	Yes	Yes	No	No	No	Yes	Yes	No	No	No	4
Rahman ME et al, 1994	Yes	No	No	No	No	Yes	Yes	No	No	No	3
Rahman ME et al, 1996	Yes	No	No	No	No	Yes	Yes	No	No	No	3
Chiang H et al, 1995	Yes	No	Yes	No	No	Yes	Yes	No	No	No	4
Chiba K et al, 1996	Yes	No	No	No	No	Yes	Yes	No	No	No	3
Ritter EJ et al, 1971	Yes	No	No	No	No	Yes	Yes	No	No	No	3
Kasubuchi Y et al, 1977	Yes	No	No	No	No	Yes	Yes	No	No	No	3
Adlard BP et al, 1975	Yes	No	No	No	No	Yes	Yes	No	No	No	3
Elmer GI et al, 2004	Yes	No	No	No	Yes	Yes	Yes	No	No	No	4
Cano F et al, 2008	Yes	No	No	No	No	Yes	Yes	No	No	No	3
Ritter EJ et al, 1973	Yes	No	No	No	No	Yes	Yes	No	No	No	3
Scott WJ et al, 1975	Yes	No	No	No	No	Yes	Yes	No	No	No	3
Goto T et al, 1987	Yes	No	No	No	No	Yes	Yes	No	No	No	3
Endo A et al, 1987	Yes	No	No	No	No	Yes	Yes	No	No	No	3
Rahman ME et al, 1995	Yes	No	No	No	No	Yes	Yes	No	No	No	3
Chaube S et al, 1968	Yes	No	No	No	No	Yes	Yes	No	No	No	3

Supplementary Table S4: Newcastle-Ottawa Scale for cohort-like animal studies.

Author, Year	**Selection**				**Comparability**		**Outcome**			**Total**
	1. Representativeness of exposed cohort	2. Selection of the non exposed cohort	3. Ascertainment of exposure	4. Demonstration that outcome of interest was not present at start of study	5a. Comparable on key factors (age/weight)	5b. Comparable on additional confounders	6. Assessment of outcome	7. Was follow-up long enough for outcomes to occur	8. Adequacy of follow up of cohorts	
Ren Liu et al., 2024	★	★	★	★	★	☆	★	★	★	**8**

Alexander TC et al, 2018	★	★	★	★	★	☆	★	★	★	8
Namoju R et al, 2021	★	★	★	★	★	☆	☆	★	★	7
de Souza Silva PM et al, 2018	★	★	★	★	★	☆	☆	★	★	7
Zhao X et al, 2020	★	★	★	★	★	☆	☆	★	★	7
Balci YI et al, 2017	★	★	★	★	★	★	★	★	★	9
Fremouw T et al, 2012	★	★	★	★	★	☆	★	★	★	8
Jimenez JJ et al,1992	★	★	★	★	★	☆	☆	★	★	7
Jimenez JJ et al,1992	★	★	★	★	★	☆	☆	★	★	7
Shuzhen Han et al., 2023	★	★	★	★	★	☆	☆	★	★	7
Sun F et al, 2019	★	★	★	★	☆	☆	☆	★	★	6
**Li JJ et al, 2023**	★	★	★	★	★	☆	★	★	★	8
Li CQ et al, 2008	★	★	★	★	★	★	★	★	★	9
Palo AK et al, 2009	★	★	★	★	★	☆	★	★	★	8
Rootman J et al, 1983	★	★	★	★	★	☆	☆	★	☆	6
Lee JY et al,2018	★	★	★	☆	★	☆	☆	★	★	6
Ramos MG et al, 1997	★	★	★	★	★	☆	★	★	★	8
Castañeda-Yslas IY et al, 2024	★	★	★	★	★	☆	☆	★	★	7
Chilaka KN et al, 2024	★	★	★	★	★	☆	☆	★	★	7
Bilgin AO et al, 2020	★	★	★	★	★	☆	☆	★	★	7
Chu W et al, 2023	★	★	★	★	★	★	★	★	★	9
Guzmán DC et al, 2018	★	★	★	★	★	☆	☆	★	★	7
Guzmán DC et al, 2016	★	★	★	★	★	☆	☆	★	★	7
Zhu RJ et al, 2013	★	★	★	★	★	☆	☆	★	★	7
Patel RS et al, 2012	★	★	★	★	★	☆	☆	★	★	7
Koros C et al, 2007	★	★	★	★	★	★	★	★	★	9
Koros C et al, 2009	★	★	★	★	★	★	★	★	★	9
Saif A-J et al, 2024	★	★	★	☆	★	☆	☆	★	★	6
Minden MD et al, 2024	★	★	★	★	★	☆	☆	★	★	7

Park M-R et al, 2023	★	★	★	★	★	☆	☆	★	★	7
Kolure R et al, 2023	★	★	★	☆	★	☆	☆	★	★	6
Guan Z et al, 2023	★	★	★	★	★	★	★	★	★	9
Salimi A et al, 2023	★	★	★	★	★	☆	☆	★	★	7
Khaleel B et al, 2022	★	★	★	★	★	☆	☆	★	★	7
Saif A-J et al, 2020	★	★	★	☆	★	☆	☆	★	★	6
Dudina MO et al, 2018	★	★	★	☆	★	☆	☆	★	★	6
Porsani MYH et al, 2017	★	★	★	★	★	☆	☆	★	★	7
Takano T et al, 2006	★	★	★	☆	★	☆	☆	★	★	6
Elli M et al, 2009	★	★	★	★	★	☆	☆	★	★	7
Sun B et al ,2009	★	★	★	★	★	☆	☆	★	★	7
Diets-Ouwehand JJAT et al, 1992	★	★	★	☆	★	☆	☆	★	★	6
Watanabe S et al, 1992	★	★	★	☆	★	☆	☆	★	★	6
Yamauchi H et al, 2004	★	★	★	★	★	☆	☆	★	★	7
Percy DH et al, 1977	★	★	★	☆	★	☆	☆	★	★	6
Shimada M et al, 1975	★	★	★	☆	★	☆	☆	★	★	6
Ramos MG et al, 1999	★	★	★	☆	★	☆	☆	★	★	6
Hussein AM, 1995	★	★	★	★	★	☆	☆	★	★	7
Percy DH et al, 1974	★	★	★	☆	★	☆	☆	★	★	6
Yamano T et al, 1983	★	★	★	☆	★	☆	☆	★	★	6
Percy DH, 1975	★	★	★	☆	★	☆	☆	★	★	6
Shimada M et al, 1973	★	★	★	☆	★	☆	☆	★	★	6
Guzmán DC et al, 2024	★	★	★	★	★	☆	☆	★	★	7
Narang HK, 1982	★	★	★	☆	★	☆	☆	★	★	6
Yamauchi H et al, 2003	★	★	★	★	★	☆	★	★	★	8
Matsutani T et al, 1983	★	★	★	☆	★	☆	☆	★	★	6
Chwalinski S et al, 1989	★	★	★	☆	★	☆	☆	★	★	6
Kaufman HE et al, 1964	★	★	★	★	★	☆	★	★	★	8

https://doi.org/10.3390/cimb48010004

Yamano T et al, 1980	★	★	★	☆	★	☆	☆	★	★	6
Hagiwara S et al, 2011	★	★	★	★	★	☆	★	★	★	8
Orth JM et al, 1988	★	★	★	☆	★	☆	☆	★	★	6
Wang J et al, 2018	★	★	★	★	★	☆	☆	★	★	7
Han S et al, 2023	★	★	★	★	★	☆	☆	★	★	7
Chen T, 1982	★	★	★	☆	★	☆	☆	★	★	6
Yamauchi H et al, 2004	★	★	★	★	★	☆	☆	★	★	7
Kochhar DM et al, 1978	★	★	★	★	★	☆	☆	★	★	7
Manson JM et al, 1977	★	★	★	★	★	☆	☆	★	★	7
Rahman ME et al, 1994	★	★	★	★	★	☆	☆	★	★	7
Rahman ME et al, 1996	★	★	★	★	★	☆	☆	★	★	7
Chiang H et al, 1995	★	★	★	★	★	☆	☆	★	★	7
Chiba K et al, 1996	★	★	★	★	★	☆	☆	★	★	7
Ritter EJ et al, 1971	★	★	★	★	★	☆	☆	★	★	7
Kasubuchi Y et al, 1977	★	★	★	★	★	☆	☆	★	★	7
Adlard BP et al, 1975	★	★	★	★	★	☆	☆	★	★	7
Elmer GI et al, 2004	★	★	★	★	★	☆	☆	★	★	7
Cano F et al, 2008	★	★	★	☆	★	☆	☆	★	★	6
Ritter EJ et al, 1973	★	★	★	☆	★	☆	☆	★	★	6
Scott WJ et al, 1975	★	★	★	☆	★	☆	☆	★	★	6
Goto T et al, 1987	★	★	★	★	★	☆	☆	★	★	7
Endo A et al, 1987	★	★	★	☆	★	☆	☆	★	★	6
Rahman ME et al, 1995	★	★	★	☆	★	☆	☆	★	★	6
Chaube S et al, 1968	★	★	★	☆	★	☆	☆	★	★	6

https://doi.org/10.3390/cimb48010004

Supplementary Table S5: ARRIVE 2.0 Essential 10 guidelines checklist

Author, Year	Study design	Sample size	Inclusion and exclusion criteria	Randomisation	Blinding	Outcome measures	Statistical methods	Experimental animals	Experimental procedures	Results	Total
Ren Liu et al., 2024	Yes	Partial	Yes	Yes	Yes	Yes	Yes	Yes	Yes	Yes	9.5
Alexander TC et al, 2018	Yes	Partial	Yes	No	Partial	Yes	Yes	Yes	Yes	Yes	8
Namoju R et al, 2021	Yes	Partial	Yes	No	No	Yes	Yes	Yes	Yes	Yes	7.5
de Souza Silva PM et al, 2018	Yes	Partial	Yes	Yes	No	Yes	Yes	Yes	Yes	Yes	8.5
Zhao X et al, 2020	Yes	Partial	Yes	No	No	Yes	Yes	Yes	Yes	Yes	7.5
Balci YI et al, 2017	Yes	Partial	Yes	Yes	Partial	Yes	Yes	Yes	Yes	Yes	9
Fremouw T et al, 2012	Yes	Partial	No	No	Yes	Yes	Yes	Yes	Yes	Yes	7.5
Jimenez JJ et al,1992	Yes	No	No	No	No	Yes	No	Yes	Yes	Yes	5
Jimenez JJ et al,1992	Yes	No	No	No	No	Yes	No	Yes	Yes	Yes	5
Shuzhen Han et al., 2023	Yes	No	No	No	No	Yes	Yes	Yes	Yes	Yes	6
Sun F et al, 2019	Yes	No	No	No	No	Yes	No	Yes	Yes	Yes	5
**Li JJ et al, 2023**	Yes	Partial	Yes	Yes	Yes	Yes	Yes	Yes	Yes	Yes	9.5
Li CQ et al, 2008	Yes	Partial	Yes	Yes	Yes	Yes	Yes	Yes	Yes	Yes	9.5

https://doi.org/10.3390/cimb48010004

Palo AK et al, 2009	Yes	Partial	Yes	Yes	No	Yes	Yes	Yes	Yes	Yes	8.5
Rootman J et al, 1983	Yes	Partial	No	No	No	Yes	No	Yes	Yes	Yes	5.5
Lee JY et al,2018	Yes	No	No	No	No	Yes	Yes	Yes	No	Yes	5
Ramos MG et al, 1997	Yes	No	No	No	Yes	Yes	Yes	Yes	Yes	Yes	7
Castañeda-Yslas IY et al, 2024	Yes	Partial	Yes	No	No	Yes	Yes	Yes	Yes	Yes	7.5
Chilaka KN et al, 2024	Yes	Partial	Yes	Yes	No	Yes	Yes	Yes	Yes	Yes	8.5
Bilgin AO et al, 2020	Yes	Partial	Yes	No	No	Yes	Yes	Yes	Yes	Yes	7.5
Chu W et al, 2023	Yes	Partial	Yes	Yes	Yes	Yes	Yes	Yes	Yes	Yes	9.5
Guzmán DC et al, 2018	Yes	Partial	Yes	No	No	Yes	Yes	Yes	Yes	Yes	7.5
Guzmán DC et al, 2016	Yes	Partial	Yes	Yes	No	Yes	Yes	Yes	Yes	Yes	8.5
Zhu RJ et al, 2013	Yes	Partial	Yes	No	No	Yes	Yes	Yes	Yes	Yes	7.5
Patel RS et al, 2012	Yes	Partial	Yes	Yes	No	Yes	Yes	Yes	Yes	Yes	8.5
Koros C et al, 2007	Yes	No	Yes	Yes	Yes	Yes	Yes	Yes	Yes	Yes	9
Koros C et al, 2009	Yes	No	Yes	Yes	Yes	Yes	Yes	Yes	Yes	Yes	9
Saif A-J et al, 2024	Yes	Partial	Yes	No	No	Yes	Yes	Yes	Yes	Yes	7.5
Minden MD et al, 2024	Yes	Partial	Yes	Yes	No	Yes	Yes	Yes	Yes	Yes	8.5

https://doi.org/10.3390/cimb48010004

Park M-R et al, 2023	Yes	No	Yes	No	No	Yes	Yes	Yes	Yes	Yes	7
Kolure R et al, 2023	Yes	Partial	Yes	Yes	No	Yes	Yes	Yes	Yes	Yes	8.5
Guan Z et al, 2023	Yes	Partial	Yes	Yes	No	Yes	Yes	Yes	Yes	Yes	8.5
Salimi A et al, 2023	Yes	Partial	Yes	No	No	Yes	Yes	Yes	Yes	Yes	7.5
Khaleel B et al, 2022	Yes	No	Yes	No	No	Yes	Yes	Yes	Yes	Yes	7
Saif A-J et al, 2020	Yes	Partial	Yes	No	No	Yes	No	Yes	Yes	Yes	6.5
Dudina MO et al, 2018	Yes	Partial	Yes	No	No	Yes	Yes	Yes	Yes	Yes	7.5
Porsani MYH et al, 2017	Yes	Partial	Yes	No	No	Yes	Yes	Yes	Yes	Yes	7.5
Takano T et al, 2006	Yes	Partial	Yes	No	No	Yes	Yes	Yes	Yes	Yes	7.5
Elli M et al, 2009	Yes	Partial	Yes	No	Partial	Yes	Yes	Yes	Yes	Yes	8
Sun B et al ,2009	Yes	Partial	Yes	Partial	No	Yes	Yes	Yes	Yes	Yes	8
Diets-Ouwehand JJAT et al, 1992	Yes	Partial	Yes	No	No	Yes	No	Yes	Yes	Yes	6.5
Watanabe S et al, 1992	Yes	Partial	Yes	No	No	Yes	Yes	Yes	Yes	Yes	7.5
Yamauchi H et al, 2004	Yes	Partial	Yes	No	No	Yes	Yes	Yes	Yes	Yes	7.5
Percy DH et al, 1977	Yes	Partial	Yes	No	No	Yes	No	Yes	Yes	Yes	6.5

https://doi.org/10.3390/cimb48010004

Shimada M et al, 1975	Yes	Partial	Yes	No	No	Yes	No	Yes	Yes	Yes	6.5
Ramos MG et al, 1999	Yes	No	No	No	No	Yes	No	No	Yes	Yes	4
Hussein AM, 1995	Yes	Partial	Yes	No	No	Yes	No	Yes	Yes	Yes	6.5
Percy DH et al, 1974	Yes	No	No	No	No	Yes	No	Yes	Yes	Yes	5
Yamano T et al, 1983	Yes	Partial	No	No	No	Yes	No	Yes	Yes	Yes	5.5
Percy DH, 1975	Yes	No	No	No	No	Yes	No	Yes	Yes	Yes	5
Shimada M et al, 1973	Yes	Partial	No	No	No	Yes	No	Yes	Yes	Yes	5.5
Guzmán DC et al, 2024	Yes	Partial	Yes	Yes	No	Yes	Yes	Yes	Yes	Yes	8.5
Narang HK, 1982	Yes	Partial	Yes	No	No	Yes	Yes	Yes	Yes	Yes	7.5
Yamauchi H et al, 2003	Yes	Partial	Yes	Partial	No	Yes	Yes	Yes	Yes	Yes	8
Matsutani T et al, 1983	Yes	No	Yes	No	No	Yes	Yes	Yes	Yes	Yes	7
Chwalinski S et al, 1989	Yes	Partial	Yes	No	No	Yes	Yes	Yes	Yes	Yes	7.5
Kaufman HE et al, 1964	Yes	No	No	No	Yes	Yes	No	Yes	Yes	Yes	6
Yamano T et al, 1980	Yes	Partial	Yes	No	No	Yes	No	Yes	Yes	Yes	6.5
Hagiwara S et al, 2011	Yes	No	No	No	Yes	Yes	Yes	Yes	Yes	Yes	7
Orth JM et al, 1988	Yes	No	No	No	No	Yes	Yes	Yes	Yes	Yes	6

https://doi.org/10.3390/cimb48010004

Wang J et al, 2018	Yes	Partial	Yes	No	No	Yes	Yes	Yes	Yes	Yes	7.5
Han S et al, 2023	Yes	Partial	Yes	No	No	Yes	Yes	Yes	Yes	Yes	7.5
Chen T, 1982	Yes	No	No	No	No	Yes	Yes	Yes	Yes	Yes	6
Yamauchi H et al, 2004	Yes	No	No	No	No	Yes	Yes	Yes	Yes	Yes	6
Kochhar DM et al, 1978	Yes	Partial	Yes	No	No	Yes	Yes	Yes	Yes	Yes	7.5
Manson JM et al, 1977	Yes	Partial	Yes	No	No	Yes	No	Yes	Yes	Yes	6.5
Rahman ME et al, 1994	Yes	Partial	Yes	No	No	Yes	Yes	Yes	Yes	Yes	7.5
Rahman ME et al, 1996	Yes	Partial	Yes	No	No	Yes	Yes	Yes	Yes	Yes	7.5
Chiang H et al, 1995	Yes	Partial	Yes	Yes	No	Yes	Yes	Yes	Yes	Yes	8.5
Chiba K et al, 1996	Yes	Partial	Yes	No	No	Yes	Yes	Yes	Yes	Yes	7
Ritter EJ et al, 1971	Yes	Partial	Yes	No	No	Yes	No	Yes	Yes	Yes	6.5
Kasubuchi Y et al, 1977	Yes	Partial	Yes	No	No	Yes	No	Yes	Yes	Yes	6.5
Adlard BP et al, 1975	Yes	Partial	Yes	No	No	Yes	Yes	Yes	Yes	Yes	7.5
Elmer GI et al, 2004	Yes	Partial	Yes	No	No	Yes	Yes	Yes	Yes	Yes	7.5
Cano F et al, 2008	Yes	No	No	No	No	Yes	No	Yes	Yes	Yes	5
Ritter EJ et al, 1973	Yes	No	No	No	No	Yes	No	Yes	Yes	Yes	5

Scott WJ et al, 1975	Yes	Partial	No	No	No	Yes	No	Yes	Yes	Yes	5.5
Goto T et al, 1987	Yes	Partial	Yes	No	No	Yes	No	Yes	Yes	Yes	6.5
Endo A et al, 1987	Yes	No	Yes	No	No	Yes	Yes	Yes	Yes	Yes	7
Rahman ME et al, 1995	Yes	No	No	No	No	Yes	No	Yes	Yes	Yes	5
Chaube S et al, 1968	Yes	Partial	Yes	No	No	Yes	No	Yes	Yes	Yes	6.5

Supplementary Table S6. Results from individual studies. Note: ACC, anterior cingulate cortex; ALA, α-lipoic acid; ALL, acute lymphoblastic leukemia; AML, acute myeloid leukemia; AMP, antimicrobial peptide; AQP5, aquaporin-5; Ara-C, cytarabine (cytosine arabinoside); AS-IV, astragaloside IV; AWAT2, acyl-CoA wax alcohol acyltransferase 2; BM, bone marrow; BrdU, bromodeoxyuridine; CA, cornu ammonis; CAT, catalase; CFS, corneal fluorescein staining; CNS, central nervous system; CTX, cyclophosphamide; DG, dentate gyrus; DHE, dihydroethidium; DNA, deoxyribonucleic acid; EM, electron microscopy; ER, endoplasmic reticulum; FGF4, fibroblast growth factor 4; FoxO, forkhead box O; GD, gestational day; GFAP, glial fibrillary acidic protein; GI, gastrointestinal; GSH, glutathione; GSH-Px, glutathione peroxidase; GST, glutathione S-transferase; H&E, hematoxylin and eosin; HMGCR, 3-hydroxy-3-methylglutaryl-CoA reductase; HO-1, heme oxygenase-1; IHC, immunohistochemistry; IF, immunofluorescence; IL, interleukin; IMD, immune deficiency pathway; iNOS, inducible nitric oxide synthase; ISC, intestinal stem cell; IV, intravenous; JAK, Janus kinase; LG, lacrimal gland; MG, meibomian gland; MGD, meibomian gland dysfunction; MI, metaphase I; MDA, malondialdehyde; MMP, mitochondrial membrane potential; MTX, methotrexate; MWM, Morris water maze; NAC, N-acetylcysteine; Nrf2, nuclear factor erythroid 2–related factor 2; OSI, oxidative stress index; PAS, periodic acid–Schiff; PCNA, proliferating cell nuclear antigen; PCE, polychromatic erythrocyte; PH3, phospho-histone H3; PI3K, phosphoinositide 3-kinase; PPARγ, peroxisome proliferator-activated receptor gamma; qRT-PCR, quantitative reverse-transcription polymerase chain reaction; ROS, reactive oxygen species; SOD, superoxide dismutase; TEM, transmission electron microscopy; TNF-α, tumor necrosis factor alpha; TOS, total oxidant status; TAC, total antioxidant capacity; VEGFR, vascular endothelial growth factor receptor; ZO-1, zonula occludens-1. Arrows: ↑, increase or upregulation; ↓, decrease or downregulation.

Author, Year	Ara-C Regimen	Animal Model	Experimental Groups	Group size	Main toxicity assessed	Objective	Results	Main histopathologic findings of Ara-C
Liu et al., 2024 [57]	Intraperitoneal Ara-C 50 mg/kg once daily × 7 days (pilot experiments showed 100	C57BL/6J male mice, 6–8 weeks old (body weight ~20–25 g at baseline)	Saline control (10 mL/kg i.p.) Ara-C only (50 mg/kg i.p. × 7 days)	n = 6–12 mice per group	Chemotherapy-induced meibomian gland dysfunction (MGD) and	To define how systemic cytarabine chemotherapy perturbs MG structure and function in vivo,	• Ara-C (50 mg/kg × 7d) produced: – ↑CFS score (punctate erosion) and ↓tear volume (~50%)	• MG: acinar atrophy, duct dilation with intraductal lipid, full-thickness ductal hyperkeratinization

https://doi.org/10.3390/cimb48010004

	mg/kg for 7 days killed ~33 % of animals, so 50 mg/kg was used for all end-point studies)		Ara-C + rosiglitazone (Ara-C 50 mg/kg i.p. × 7 days + rosiglitazone 10 mg/kg by oral gavage × 7 days)		ocular-surface injury, including: – Corneal epithelial defects – Lacrimal gland (LG) hyposecretion – Meibomian gland (MG) plugging, acinar dropout and ductal hyperkeratinization	elucidate underlying molecular mechanisms and test whether PPARγ activation by rosiglitazone mitigates those effects	– LG dysfunction (↓α-SMA in myoepithelium, ↓AQP5) – MG orifice plugging, ↓acinar area (~40%) – ↓PCNA, P63 and Lrig1 (impaired proliferation & progenitors) – Duct dilation, intraductal lipid stasis, ↑K1/K10 (hyperkeratinization) – ↓PPARγ nuclear localization, ↓AWAT2 / SOAT1 / ELOVL4, ↑HMGCR & cholesterol – ↓p-AKT, p-FoxO1 / FoxO3a and nuclear FoxO1 / FoxO3a translocation – ↑4-HNE and 8-OHdG; ↑Keap1, ↓Nrf2 / HO-1 / SOD1 (oxidative stress)	(K1/K10+), loss of proliferating/progenitor basal cells • LG: myoepithelial degeneration (↓α-SMA), reduced AQP5
Alexander TC et al, 2018 [22]	Cytarabine (Ara-C) 5 mg/kg co-administered with methotrexate (MTX) 10 mg/kg via single i.t. injection each week for 3 weeks	Juvenile male C57BL/6 mice (postnatal day 21; ~10–12 g at injection start)	• Saline controls: i.t. 0.1 % saline weekly×3 weeks • Chemotherapy: i.t. MTX + Ara-C weekly×3 weeks	n = 10 each	Chemotherapy-induced neurotoxicity: hippocampal-dependent cognitive impairment and dendritic degeneration	To determine whether intrathecal MTX + Ara-C impairs spatial memory and alters hippocampal dendritic morphology in a juvenile murine model of ALL therapy	• Cognitive – MTX + Ara-C mice failed to show quadrant preference or normal platform crossings in MWM probe trials ($p < 0.05$) despite intact swimming speed and learning curves. • Dendrites – Significant reductions in Sholl intersections (DG: 60–180 μm; CA1 apical 60–180 μm/CA1 basal 40–120 μm;	No frank necrosis or cell loss, but pronounced dendritic atrophy, reduced arbor complexity, and selective loss of mature (mushroom) spines across DG, CA1 and CA3 hippocampal subregions

							CA3 apical 80–160 μm/CA3 basal 60–140 μm from soma; p < 0.05) and total dendritic length, branch points, and tips in all hippocampal subfields (p < 0.05–0.001).<br>• Spines – Mushroom-spine density markedly decreased in DG (–50 %), CA1 apical, and CA3 apical neurons (p < 0.05); thin and stubby spines largely spared.<br>• Inflammation – Elevated circulating leukocytes per mL blood in MTX + Ara-C group (p < 0.05), suggestive of systemic, intrathecal-driven inflammation	
Namoju R et al, 2021 [80]	Intraperitoneal cytarabine (Ara-C) at 12.5 or 25 mg/kg once daily on gestational days 8–14	Pregnant rats (180–220 g body weight; 3–4 months old)	Saline control (i.p. 0.9% NaCl)<br><br>ALA only (200 mg/kg orally)<br><br>Ara-C 12.5 mg/kg<br><br>Ara-C 25 mg/kg	n=6 dams each	Developmental (fetotoxic) toxicity of prenatal Ara-C exposure: growth retardation, resorptions, malformations, skeletal ossification defects	To test whether α-lipoic acid (ALA) protects against Ara-C–induced developmental anomalies in rat fetuses	• Ara-C caused dose-dependent maternal toxicity: ↓food intake, ↓maternal weight gain, ↓placental weight, ↑placental MDA, ↓placental GSH/GSH-Px/SOD/CAT<br>• Fetal toxicity with Ara-C: ↑resorptions, ↑fetal mortality, ↓fetal weight and length,	• Fetal external anomalies (limb reduction defects, oligodactyly, brachydactyly, phocomelia, hematomas)<br><br>• Impaired ossification (skull, vertebrae, sternum, scapula, pelvic girdle, carpals, metacarpals, tarsals, metatarsals, long bones) with fewer

https://doi.org/10.3390/cimb48010004

			Ara-C 25 mg/kg + ALA 200 mg/kg				external malformations (phocomelia, digit defects, hematomas),<br>skeletal ossification delays,<br>↓ossification centers,<br>↓bone Ca and P	ossification centers and low Ca/P content
de Souza Silva PM et al, 2018 [45]	Cytarabine 1.8 mg per mouse, four intraperitoneal injections given every 12 h over 2 days	BALB/c mice, ~70 days old (sex not specified; weight not reported)	Saline O.A. + saline I.P.<br><br>Saline O.A. + Ara-C I.P.<br><br>ß-D-glucan O.A. + saline I.P.<br><br>Glutamine O.A. + saline I.P.<br><br>ß-D-glucan + glutamine O.A. + saline I.P.<br><br>ß-D-glucan O.A. + Ara-C I.P.<br><br>Glutamine O.A. + Ara-C I.P.<br><br>ß-D-glucan +	n = 3 mice/group; eight groups total	Genomic instability (DNA strand breaks in leukocytes), leukopenia, and impaired intestinal crypt cell mitosis induced by Ara-C	To test whether dietary ß-D-glucan and/or glutamine protect against Ara-C–induced DNA damage in blood cells, leukopenia, and reduced intestinal mitotic index in mice	5-fold rise in DNA-damage score,<br>~50% leukopenia,<br>↑neutrophils,<br>↓lymphocytes,<br>−50% intestinal-crypt mitoses vs. saline.	No overt mucosal necrosis, but 50 % loss of mitotic activity in intestinal crypt epithelium

			glutamine O.A. + Ara-C I.P.					
Zhao X et al, 2020 [81]	Single intraperitoneal dose of cytarabine (100 mg/kg) at mid-gestation: injected on gestational day (GD) 11.5, 12.5, 13.5 or 14.5 to timed-pregnant rats	Sprague–Dawley rats; females 8–10 weeks old, 200–250 g; mated and dated by vaginal smear, GD 0 = day of sperm detection	• Four Ara-C groups: single 100 mg/kg i.p. on GD 11.5, 12.5, 13.5 or 14.5<br>• Four matched saline-injected controls (i.p. 0.9% NaCl on same GDs)	n = 8 dams per group	Cytarabine-induced congenital malformation (polydactyly - syndactyly) of the forelimb thumb	To determine whether cytarabine causes thumb polydactyly in rat embryos and to assess FGF4 expression changes in the limb bud underlying that defect	• Ara-C at GD 12.5 yielded the highest incidence (85%) of thumb polydactyly/syndactyly<br><br>• In situ hybridization: FGF4 expression domain at limb-bud apex was 3–4× larger and persisted longer in Ara-C vs. saline controls (12.5 d: 84.8% vs. 30.8% area; p<0.01)<br><br>• Histology and cartilage staining: extra metacarpal and phalanx visible in malformed limbs<br><br>• X-ray: confirmed supernumerary metacarpal and phalanx in deformed thumbs	Discrete extra skeletal elements: one additional metacarpal and one additional phalanx; syndactylous fusion of thumb rays in ~85% of treated embryos
Balci YI et al, 2017 [58]	Cytosine-arabinoside (Ara-C) 400 mg/kg intraperitoneally once daily for 5 days	Mature Wistar albino rats (4 months old; ~250 g)	Ara-C only (400 mg/kg × 5 days)<br><br>Ara-C + N-acetylcysteine (NAC 200 mg/kg i.p. daily × 5 days)<br><br>NAC only (200	n=10 rats/group	Ara-C–induced ocular oxidative stress (keratoconjunctivitis model)	To test if NAC supplementation attenuates Ara-C–induced oxidative stress in rat cornea and conjunctiva	↑TOS (19.3±7.7 vs. 1.00±0.34 µmol H2O2 eqv.; P=0.002)<br><br>↑OSI (1.51±0.65 vs. 0.17±0.07; P=0.04) vs. control	N/A

			mg/kg × 5 days)<br><br>Control (saline injections)					
Fremouw T et al, 2012 [23]	Cytosine-arabinofuranoside (ARA-C) 275 mg/kg i.p., once daily×5 days	Male C57BL/6J mice, 8 weeks old (≈20–25 g)	• Saline (vehicle) + recent-memory test<br><br>• Saline + remote-memory test<br><br>• ARA-C + recent-memory test<br><br>• ARA-C + remote-memory test	n≈15 per group	Chemotherapy-induced cognitive dysfunction (“chemo-fog”)	To determine whether ARA-C impairs spatial learning and short-term (1 d) or long-term (30 d) memory in the Morris water maze (MWM) in mice	• ARA-C caused ~9% weight loss vs. ~+2% in controls ($p<0.001$)<br><br>• No ARA-C effect on acquisition latency or distance ($p>0.5$)<br><br>• No deficit in recent or remote memory (all probe measures $p>0.29$)<br><br>• Numerical trends favored ARA-C group on all cognitive measures	N/A
Jimenez JJ et al,1992 [64]	Cytarabine 20 mg/kg i.p. once daily × 7 days	7-day-old Fisher rats (~12–18 g at start of experiment) transplanted i.p. with C51 chloroleukemia cells	Vehicle control (0.2 mL buffer)<br><br>ARA-C alone (20 mg/kg i.p. daily × 7 days)<br><br>rHu-IL-1β alone (0.25 µg i.p. daily × 7 days)<br><br>ARA-C + rHu-IL-1β (same doses, co-	n = 10 rats per group	ARA-C–induced alopecia and failure to suppress chloroleukemia	To test whether rHu-IL-1β synergizes with ARA-C to abort C51 chloroleukemia while preventing ARA-C–induced alopecia in rats	– Vehicle: 0/10 long-term survivors (0%)<br><br>– ARA-C alone: 1/10 survivors (10%)<br><br>– ARA-C alone: 100% of rats developed complete body alopecia by day 10	N/A

https://doi.org/10.3390/cimb48010004

			administered daily × 7 days)					
Jimenez JJ et al,1992 [65]	50 mg/kg cytarabine (Ara-C) i.p. once daily for 4–5 days	Sprague-Dawley rats at 7 days old	– Parenteral: 2 groups × 10 rats (CTX 50 mg/kg + Ara-C 50 mg/kg × 5 days ± ImuVert 10 µg + NAC 4 mg s.c. × 5 days)<br><br>– Topical: 5 groups × 10 rats (CTX 50 mg/kg + Ara-C 50 mg/kg × 4 days; treatments applied daily in 0.25 mL liposomal suspension)	n = 10 rats per group	Chemotherapy-induced alopecia	(1) Determine if N-acetylcysteine (NAC) prevents cyclophosphamide (CTX)-induced alopecia<br><br>(2) Test whether ImuVert + NAC protects against alopecia from combined CTX + Ara-C	• CTX (50 mg/kg) + Ara-C (50 mg/kg × 5d) + PBS → 100% alopecia<br><br>• CTX + Ara-C + ImuVert/NAC (s.c.) → 7/10 rats only mild alopecia (1+), 2/10 moderate (2+)<br><br>• Topical ImuVert/NAC (liposomes) during CTX + Ara-C → 9/9 rats showed thick hair regrowth over most of the back; 3/9 complete protection	N/A
Han et al., 2023 [55]	Cytarabine (Ara-C) was dissolved in fly food to final concentrations of 0, 1, 5 and 10 mM. Larvae were exposed continuously from egg laying through pupation. Adult flies (2–3 days	Drosophila melanogaster (w^1118 strain; plus gstD1-GFP and esg-Gal4,UAS-GFP reporters)	Four treatment groups: 0 (control), 1, 5 and 10 mM Ara-C.<br><br>• Development: ~100 eggs/vial × 3 vials/group (n = 9 total)<br><br>• Larval crawling: 5 larvae/group × 2 experiments		• Developmental delay and growth defects<br><br>• Reduced longevity<br><br>• Impaired locomotor function (larval	To establish D. melanogaster as an in vivo model for probing cytarabine's side-effect profile and underlying mechanisms, and to enable future screening of protective agents	• Development: 10 mM Ara-C delayed egg→pupa median time by ~15%, reduced pupal count and sharply cut eclosion rate. Female adult weight dropped at 1 mM.<br><br>• Locomotion: larval crawling speed/track length and adult climbing index declined dose-dependently.	• Midgut epithelial cells: mild cytoplasmic edema, apical displacement of nuclei<br><br>• Brush border: sparse, truncated microvilli<br><br>• Mitochondria: outer-membrane

	old at the start) were maintained on Ara-C–supplemented food for 10 days.	• Lifespan: 20 flies/vial × 2–3 vials/replicate × 5–6 replicates/sex<br>• Climbing: 20 flies/vial × 8 vials/group × 3 trials<br>• Food-intake: 10 flies/vial × 3 replicates<br>• Stress resistance (H_2O_2, SDS, starvation): 20 flies/vial × 7–10 replicates/sex<br>• Enzyme assays (SOD, CAT): 20 flies/sample × 6–10 samples<br>• Histology/TEM/IHC/qPCR: 3–5 guts × 3 biological replicates	crawling, adult climbing)<br>• Decreased feeding and stress resilience<br>• Intestinal morphological and ultrastructural damage, oxidative injury, inflammation and apoptosis	• Longevity: lifespan shortened in both sexes at ≥1 mM.<br>• Feeding/stress: dose-dependent food-intake reduction in both sexes; females lost starvation resilience at ≥5 mM. Survival under H_2O_2 declined in both sexes, while SDS challenge paradoxically yielded higher survival in Ara-C groups alongside downregulation of JAK-STAT and JNK genes.<br>• Oxidative stress: females showed raised CAT enzyme activity and upregulated gstD1/2 mRNA; no SOD change.<br>• Intestinal damage: no “Smurfs” (no gross permeability loss), but 10 mM Ara-C shortened and thinned midguts. TEM revealed epithelial-cell edema, apical nuclear migration, truncated/sparse microvilli, and ruptured	rupture, rarefied matrix

							mitochondrial membranes.<br><br>• ISC proliferation & ROS: 10 mM Ara-C boosted esg+ cell counts (~25%), increased pH3+ mitoses (~30%), elevated DHE and gstD1-GFP fluorescence (~20–40%), and upregulated gut gstD1/2 mRNA.<br><br>• Immune/apoptosis: gut expression of Toll (myd88, dif, drs), IMD (imd, rel, dpt), AMP (AttA, dro, mtk) and apoptotic (reaper, drice, dcp-1) genes rose significantly under 10 mM Ara-C.	
Sun F et al, 2019 [70]	2.5 mg/kg Ara-C i.p. once daily for 5, 10, 15 or 20 d	BALB/c nude mice, 5–8 weeks old, 18–20 g	control (saline) vs Ara-C (2.5 mg/kg)	n per arm not specified	Ara-C–induced impairment of mouse growth (body-weight loss) and organ atrophy (kidney, liver, lung)	To dissect how Ara-C triggers G_1/S cell-cycle arrest—via upregulation of INK4 family inhibitors (CDKN2A–D) and/or direct blockade of the CDK4/cyclin D1 complex—and to explore	• In nude mice, Ara-C markedly shrinks tumor volume & weight in a time-dependent manner.<br><br>• In normal mice, Ara-C causes dose-dependent body-weight loss and atrophy of kidney, liver & lung, accompanied by CDKN2A–D induction and CDK4 / cyclin D1	Gross organ atrophy (kidney, liver, lung) and histologic evidence of renal inflammation and structural disruption (H&E), consistent with impaired tissue growth

https://doi.org/10.3390/cimb48010004

					consequences for tumor growth and normal-tissue development	suppression in those organs.	
Li JJ et al, 2023 [46]	Cytarabine (Ara-C) 100 mg/kg i.p. once daily for 7 days	Male C57BL/6 mice, 8–10 weeks old, 20–25 g	Saline control (i.p.)<br><br>Ara-C (100 mg/kg i.p. × 7 days)<br><br>Ara-C + AS-IV (100 mg/kg + astragaloside IV 10 mg/kg i.p. × 7 days)<br><br>Ara-C + AS-IV (100 mg/kg + AS-IV 20 mg/kg)<br><br>Ara-C + AS-IV (100 mg/kg + AS-IV 40 mg/kg)	n = 10 mice/group	Chemotherapy-induced intestinal mucositis (IM): weight loss, ileal epithelial injury, barrier disruption & cytokine release	To determine whether astragaloside IV (AS-IV) protects against Ara-C–induced intestinal mucositis and to define the underlying mechanism related to macrophage polarization via AKT signaling	Ara-C (100 mg/kg) induced:<br><br>– >10% weight loss & ↓ food intake by Day 3<br><br>– Predominant ileal injury: villus shortening - flattening, crypt loss, epithelial vacuolation, inflammatory infiltrate, muscle-layer edema<br><br>– ↓ Occludin & ZO-1; ↑ TNF-α, IL-6; ↓ IL-10<br><br>– ↑ CD86+ (M1) macrophages without M2 change	Ileal villus atrophy (short/flat), crypt epithelial necrosis, enterocyte vacuolation, dense inflammatory infiltrates, muscularis edema, lamina propria inflammation, muscularis edema; tight-junction loss on IHC
Li CQ et al, 2008 [24]	400 mg/kg Ara-C intraperitoneally once daily × 5 days	Male Sprague–Dawley rats, 200–250 g	• Vehicle (0.9% saline) control<br><br>• Ara-C (400 mg/kg × 5 days i.p.)	n = 20 rats per group	Long-term cognitive impairment (remote spatial memory) and neuronal	To determine how Ara-C affects spatial learning, memory (recent vs remote) and dendritic morphology in	– Learning (escape latency over 5 days) and recent memory (Day 1 probe) were intact in Ara-C rats vs controls<br><br>– Remote memory (Day 30 probe): Ara-C rats	Selective retraction of apical dendritic arbors in ACC pyramidal cells (fewer spines & branches) without overt cell loss

https://doi.org/10.3390/cimb48010004

					dendritic retraction	the anterior cingulate cortex (ACC) and hippocampal CA1	spent significantly less time in target quadrant ($p < 0.01$)<br><br>– ACC pyramidal neurons (apical dendrites only) in Ara-C rats showed:<br><br>• ~15% shorter total dendritic length ($p < 0.001$)<br><br>• ~36% fewer branch points ($p < 0.005$)<br><br>• ~51% lower spine density ($p < 0.001$) – No changes in basal ACC dendrites or in hippocampal CA1 apical-basal dendrites	
Palo AK et al, 2009 [74]	Single i.p. injection of Ara-C at 100, 150 or 200 mg/kg body-weight (one dose per animal)	Swiss albino mice, 8–10 weeks old, ~20 g	Vehicle control (0.9% NaCl)<br><br>Positive control: cyclophosphamide 40 mg/kg<br><br>Ara-C 100 mg/kg<br><br>Ara-C 150 mg/kg	Five groups (n = 30 mice each: 6 females + 24 males)	Cytogenotoxicity in bone-marrow and male germ cells, plus germline transmission	To quantify Ara-C–induced chromosomal damage in somatic (bone marrow) and germ-line cells and to test whether effects transmit via male gametes	• Bone marrow: dose-dependent ↑ aberrant metaphases and CAs (breaks, fragments, minutes) at 100–200 mg/kg ($p \leq 0.01$); no significant change in mitotic index; ↑ micronuclei ($p \leq 0.01$)<br><br>• Germline: dose-dependent ↑ aberrant spermatogonial	Random chromatid and chromosome gaps & breaks; fragments and pulverized metaphases in bone marrow; autosomal asynapsis and extra chromosomal elements in diakinesis; increased

https://doi.org/10.3390/cimb48010004

			Ara-C 200 mg/kg			metaphases & CAs at 24 h (p ≤ 0.05–0.01); ↑ atypical bivalents/univalents in primary spermatocytes at 4 weeks (p ≤ 0.01); slight, non-significant ↓ in abnormal sperm at 8 weeks	malformed sperm categories
Rootman J et al, 1983 [59]	Single dose of 37.5 mg/kg cytosine-arabinoside (Ara-C) in 0.5 ml saline, given either<br><br>– Subconjunctivally (posterior to the superior limbus) in the right eye<br><br>Or<br><br>– Intravenously (marginal ear vein)	Female New Zealand white rabbits with mean weight 2.20 ± 0.03 kg	– Pharmacokinetics: 3 rabbits/route/time-point at 0.5, 1, 2, 4, 8, 12 h (total 18 per route)<br><br>– Ocular-toxicity histology: 5 rabbits (SC injection in right eye; fellow eye saline)<br><br>– Bone-marrow toxicity (WBC counts): 2 rabbits/route	• Ocular (local) toxicity from subconjunctival dosing<br><br>• Systemic bone-marrow suppression	To compare ocular penetration, elimination kinetics, and local/systemic toxicity of an equal dose of Ara-C when given subconjunctivally versus intravenously	• Anterior chamber peak (mean ± SE): – Subconjunctival 1.305 ± 0.092 mM (at 1 h) vs IV 0.090 ± 0.014 mM → 15× higher SC<br><br>• Vitreous peak: SC 0.082 ± 0.011 mM vs IV 0.044 ± 0.002 mM → 2× higher SC<br><br>• Serum peak: SC 0.061 mM vs IV 0.170 mM<br><br>• Urinary excretion (cumulative, 12 h): SC 67% vs IV 70% of dose<br><br>• All ocular compartments reached concentrations well above in vitro cytotoxic levels for tumor cell lines<br><br>• No significant bone-marrow suppression at 37.5 mg/kg dose	• Subconjunctival injection:<br><br>– Mild, transient polymorphonuclear and lymphocytic infiltration under the conjunctiva<br><br>– Focal superficial conjunctival erosions at 12–24 h, resolved by 1 week<br><br>– Cornea, iris, ciliary body histologically normal<br><br>• Fellow-eye (saline) control: minimal transient conjunctival edema

Lee JY et al, 2018 [75]	Single intraperitoneal dose of cytosine-arabinoside (Ara-C) at 100 mg/kg per mouse	C57BL/6J mice, 7 weeks old	Wild-type (no leukemia)<br><br>Leukemia alone (C1498 cells + vehicle)<br><br>Plerixafor alone (CXCR4 antagonist)<br><br>Ara-C alone (100 mg/kg)<br><br>Ara-C + plerixafor	Five cohorts ($n \geq 3$ mice each for histology)	Impact of Ara-C (± CXCR4 antagonist) on bone-marrow microenvironment: sinusoidal vessel density and megakaryocyte counts	To test whether CXCR4 blockade (plerixafor) plus Ara-C restores bone-marrow architecture (sinusoids & megakaryocytes) in a murine AML model	• Leukemia reduced sinusoidal capillary density (~17 vs 41 per field) and megakaryocyte numbers (~5 vs 14) vs wild-type<br><br>• Ara-C + plerixafor restored sinusoids (~37/field) and megakaryocytes (~13/field), significantly above Ara-C or plerixafor alone ($P < 0.01$)	• Leukemic BM has collapsed sinusoids and scant megakaryocytes<br><br>• Ara-C + plerixafor remodels endothelium (VEGFR-3$^+$ capillaries) and replenishes megakaryocytes
Ramos MG et al, 1997 [47]	3.6 mg per mouse per day, i.p., given every 12 h for 2 days (Experiment B) or 4 days (Experiment A)	Swiss NMRI mice (30 ± 2 g), fed either a standard commercial chow (Experiment A) or an elemental diet (Experiment B)	Experiment A (commercial diet):<br><br>• Normal (no Ara-C, no SCFA), n = 5<br><br>• Ara-C + oral saline (SAL), n = 13<br><br>• Ara-C + oral SCFA solution (60:30:40 mM acetate:propionate:butyrate), n = 12<br><br>Experiment B (elemental diet):<br><br>• Ara-C + oral saline, n = 13		Chemotherapy-induced intestinal mucositis (atrophy, inflammation, necrosis)	To test whether oral short-chain fatty acids (SCFA) protect against Ara-C mucosal injury in mice on commercial vs elemental diets	Ara-C caused villus shortening, enterocyte necrosis, lamina-propria inflammation, focal hepatic necrosis; more severe on elemental diet	- Ara-C + saline: flattened/atrophic villi, focal necrosis, increased lamina-propria mononuclear cells, occasional polyps in colon, hepatic necrosis<br><br>– Ara-C + SCFA: milder villus blunting, minimal necrosis, occasional xanthomized enterocytes at high SCFA dose, hepatic xanthomization without necrosis

https://doi.org/10.3390/cimb48010004

			• Ara-C + oral SCFA (35:15:9 mM), n = 13<br><br>• Normal (no Ara-C), n = 2–3					
Castañeda-Yslas IY et al, 2024 [76]	Ara-C 6 mg/kg i.p.<br>– Single dose at 0 h (Group 2 → total 6 mg/kg)<br><br>– Three consecutive daily doses at 0, 24, 48 h (Group 3 → total 18 mg/kg)<br><br>– Variations combined with AgNPs in Groups 5–7 (all 6 mg/kg per Ara-C dose)	Male BALB/c mice (Mus musculus) 5–6 weeks 20.46 ± 2.96 g	Control: 200 μL water p.o. at 0, 24, 48 h<br><br>Ara-C × 1 (0 h)<br><br>Ara-C × 3 (0, 24, 48 h)<br><br>AgNPs × 3 (0, 24, 48 h; 6 mg/kg p.o.)<br><br>Ara-C (0 h) → AgNPs (24, 48, 72 h)<br><br>Ara-C (0, 24, 48 h) → AgNPs (72, 96, 120 h)<br><br>Alternating Ara-C (0, 48, 96 h) and AgNPs (24, 72, 120 h)	N per group: 7 animals (49 total)	• Genotoxicity: acute (micronucleated polychromatic erythrocytes, MNPCE) and cumulative (micronucleated erythrocytes, MNE)<br><br>• Cytotoxicity/myelosuppression: reduction in polychromatic erythrocytes (PCE/1000 TE)	Determine whether a defined silver-nanoparticle (AgNPs) formulation (Argovit) can protect against the genotoxic side effects of the antimetabolite Ara-C in vivo	• Ara-C alone induced:<br><br>– Transient myelosuppression (↓ PCE)<br><br>– Peak acute genotoxicity (MNPCE) at 72 h (3.9× control)<br><br>– Peak cumulative genotoxicity (MNE) at 72 h (38 ± 3.5/10,000 TE)	N/A
Chilaka KN et al, 2024 [82]	Ara-C 12.5 mg/kg and 25 mg/kg, i.p. once daily on gestational days 8–21 (14	Pregnant female rats (strain not specified)	Control (saline, GD 8–21)<br><br>ALA only (200 mg/kg p.o., GD 8–21)	n = 10 male pups per group	Prenatal Ara-C–induced male reproductive (testicular)	To determine whether maternal α-lipoic acid (ALA) supplementation protects F_1 male	Compared with controls, prenatal Ara-C produced dose-dependent:	• Seminiferous-tubule atrophy, vacuolization in germinal epithelium & interstitium

	consecutive days)		Ara-C low (12.5 mg/kg i.p., GD 8–21)<br><br>Ara-C high (25 mg/kg i.p., GD 8–21)<br><br>Ara-C high + ALA (25 mg/kg + 200 mg/kg, GD 8–21)		developmental toxicity	rats from in utero Ara-C–induced impairments in reproductive development and function	• ↓ F_1 pup body weight & delayed puberty (≈ 2–6 days delay)<br><br>• ↓ testis, epididymis, prostate & seminal-vesicle weights & sizes<br><br>• ↑ testicular MDA; ↓ SOD, GSH, GSH-Px, CAT • ↓ plasma testosterone, FSH, LH<br><br>• ↓ 3β-HSD & 17β-HSD; ↑ testicular cholesterol<br><br>• ↓ sperm count, motility, viability, seminal fructose; ↑ morphological defects<br><br>• Histomorphometry: thinner tubules, higher tubule density, lower Johnsen scores	• Disorganized/detached germ cells, cell sloughing into lumen<br><br>• Depleted or absent mature spermatozoa, increased immature germ cells<br><br>• Reduced Leydig/Sertoli cell integrity<br><br>• Epididymal tubules with sparse sperm and tubular vacuolization
Bilgin AO et al, 2020 [79]	Ara-C 200 mg/kg i.p. once daily × 14 days	Male Wistar rats, 260–280 g	– Control: saline gavage + saline i.p.<br><br>– Cytarabine: saline gavage + Ara-C 200 mg/kg i.p.<br><br>– Rutin + Cytarabine:	n=8 per group	Ara-C-induced noncardiogenic pulmonary oedema & lung oxidative - inflammatory injury	To test whether oral rutin can prevent Ara-C-associated pulmonary oedema, oxidative stress & inflammation in rats	– CT: 50% oedema - effusion in Ara-C group; none in Rutin + Ara-C<br><br>– Ara-C: ↑MDA, TOS, TNF-α, NF-κB; ↓tGSH, TAS ($p<0.0001$ vs control)<br><br>– Rutin co-treatment normalized all markers vs Ara-C alone ($p<0.0001$)	diffuse alveolar oedema, vascular congestion, chronic inflammatory infiltrates, bronchial damage

https://doi.org/10.3390/cimb48010004

			rutin 50 mg/kg oral + Ara-C 200 mg/kg i.p.					
Chu W et al, 2023 [49]	100 mg/kg Ara-C, i.p., once daily for 7 days	Male C57BL/6 mice, 6–8 weeks old, 20–25 g	Control (saline i.p.)<br><br>Ara-C alone (100 mg/kg i.p.×7 days)<br><br>Ara-C + GQBZP low dose (100 mg/kg Ara-C + 11 g/kg GQBZP, 7 + 10 days)<br><br>Ara-C + GQBZP high dose (100 mg/kg Ara-C + 22 g/kg GQBZP, 7 + 10 days)	n = 12 each	Chemotherapy-induced intestinal mucositis	To test whether Guiqi Baizhu prescription (GQBZP) can prevent Ara-C-induced mucositis in mice and to define its mechanism via macrophage polarization and JAK2/STAT1 signaling	– Ara-C caused weight loss, anorexia, villus atrophy, crypt loss, ↑ histopath scores<br><br>– Pro-inflammatory ↑IL-6, TNF-α; ↓anti-inflammatory IL-10	– Shortened, flattened villi; crypt necrosis/obliteration; epithelial vacuolization<br><br>– Lamina-propria inflammatory infiltrates; muscular-layer edema
Guzmán DC et al, 2018 [25]	Cytarabine 0.6 g/kg i.v. once daily for 5 days	Male Wistar rats, 4 weeks old, ~80 g	Control (0.9% NaCl vehicle)<br><br>Ara-C alone<br><br>Stevia alone<br><br>Ara-C + stevia<br><br>Splenda alone	n=8 each	Chemotherapy-induced neurotoxicity: biochemical and histological damage in brain regions	To determine how two non-nutritive sweeteners (stevia, Splenda) affect dopamine, 5-HIAA, oxidative-stress markers and histology in	– Ara-C alone ↓ striatal & medullary dopamine; ↓ 5-HIAA in striatum & cerebellum; ↓ GSH; ↑ TBARS in all regions; marked neuronal degeneration.	Widespread neuronal pyknosis, cell shrinkage, vacuolization, loss of nuclear detail in cortex, striatum, cerebellum/medulla

https://doi.org/10.3390/cimb48010004

			Ara-C + Splenda			young rats treated with cytarabine		
Guzmán DC et al, 2016 [26]	Cytarabine 70 mg/kg i.p. once daily for 5 days	Male Wistar rats, ≈ 100 g (~4–5 weeks old)	Control (0.9% NaCl i.p.)<br><br>Doxorubicin 1 mg/kg i.p. × 5 days<br><br>Cytarabine 70 mg/kg i.p. × 5 days<br><br>Oleic acid 1,500 µL/kg i.p. × 5 days<br><br>Dox + oleic acid (same doses)<br><br>Ara-C + oleic acid (same doses)	n = 6 each	Chemotherapy-induced oxidative/nitrosative stress and monoamine depletion in the brain	To evaluate whether oleic acid cotreatment protects against basal-ganglia and cortical oxidative damage and monoamine changes caused by cytarabine and doxorubicin in young rats	• Ara-C alone ↓ dopamine, TBARS, GSH, H2O2 & ATPase activity in all regions vs. control ($p < 0.001$)	N/A
Zhu RJ et al, 2013 [77]	Ara-C 0.5 g/kg , i.p., once daily × 4 days	C57BL/6J mice (female), 6–8 weeks old, ~20 g	– Ara-C + DMSO vehicle (“control”): n = 35<br><br>– Ara-C + BADGE (60 mg/kg i.p.	n = 35 each	Chemotherapy-induced bone-marrow adipogenesis and delayed hematopoietic recovery	To test whether BADGE (a PPARγ antagonist) prevents Ara-C–induced adipocyte hyperplasia in the marrow niche and	– Ara-C induced marrow adipocyte hyperplasia, sinus dilation & hemorrhage<br><br>– BADGE markedly reduced Ara-C–driven adipogenesis in long bones & tail BM (↓	– Long-bone BM: hypocellularity, hemorrhagic, dilated/discontinuous sinusoids, adipocyte hyperplasia<br><br>– Tail vertebrae BM: sinus dilation,

			daily × 4 wk): n = 35<br><br>– Ara-C + G-CSF (positive control, 300 μg/kg s.c. × 5 d): n = 25			thereby accelerates post-chemotherapy hematopoietic recovery	adipocyte counts, ↓PPARγ-2/aP2)<br><br>– Faster WBC/neutrophil recovery & trend↑ platelets with BADGE vs. vehicle; Hb unchanged<br><br>– No change in serum G-CSF with BADGE<br><br>– Higher CFU counts in BADGE group at weeks 2–3 post-Ara-C<br><br>– ↑ Ki-67+CD45+ BM cells, ↑ Ki-67+ LSK HSCs and HPCs with BADGE<br><br>– Enhanced CD45+ cell infiltration in fatty (tail) BM niche with BADGE<br><br>– BADGE decreased BM SDF-1α and HIF-1α expression, and restored sinus regeneration	increased PPARγ-2/aP2 expression, but fewer new adipocytes
Patel RS et al, 2012 [27]	50, 100 or 200 mg/kg cytarabine, i.p. once daily 5 days (“short-term”) or 14 days (“long-term”)	Male juvenile Sprague–Dawley rats, 40–50 g	Vehicle control (0.9% NaCl)<br><br>Ara-C 50 mg/kg/day<br><br>Ara-C 100 mg/kg/day	n = 10 rats/group	Ara-C–induced cerebellar neuronal damage and motor dysfunction	To correlate in vivo cerebellar histological/biochemical/genetic alterations with behavioral deficits following Ara-C	– Ara-C (14 d) ↓ food/water intake, ↓ body & brain weight ($p < 0.001$)<br><br>– Motor deficits: ↓ rotarod performance & locomotion; ↑ step width,	– Histology: Purkinje cell misalignment & loss; granule cell depletion in all lobes ($p < 0.001$)

			Ara-C 200 mg/kg/day			treatment in juvenile rats	↓ step length ($p < 0.05$–0.001)<br><br>– Oxidative stress: ↑ MDA, ↓ GSH in cerebellum ($p < 0.001$) – DNA damage: ↑ TL, TM, OTM, % tail DNA in neuronal comets ($p < 0.001$)<br><br>– Apoptosis: ↑ TUNEL+ granule-layer cells ($p < 0.001$)<br><br>– IHC: ↑ p53+ & caspase-3+ cells in Purkinje/molecular layers ($p < 0.001$)<br><br>– Calbindin D-28K: no change at 5 d; ↓ 14 d at 100&200 mg/kg ($p < 0.001$)<br><br>– Epigenetics: 14 d Ara-C ↑ histone H3 acetylation & H3K4me; ↓ H3K9me ($p < 0.01$–0.001)	– Granule cell layer: marked cell loss & cytotoxic changes
Koros C et al, 2007 [29]	400 mg/kg cytosine-arabinoside, intraperitoneal injection once daily for 5 days	Adult male Wistar rats (~250–350 g)	Control (saline i.p.)<br><br>Ara-C alone (400 mg/kg × 5 days)	n=10 rats each	High-dose Ara-C–induced cerebellar dysfunction/ neurotoxicity	To characterize Ara-C–induced motor coordination deficits, histological and cytoskeletal	• Ara-C caused ataxic gait (↓ step length, ↑ step width), rotarod deficits (↓ latency & active walking time), irregular swim velocity ($p<0.05$–0.001).	• Disruption of Purkinje-cell monolayer (multilinear arrangement)

			NAC alone (200 mg/kg/day p.o. for 14 days)<br><br>Ara-C + NAC (NAC for 7 days before and during the 5 days of Ara-C, total 14 days)			changes in the adult rat cerebellum and to test whether N-acetylcysteine (NAC) prevents these alterations	• Open-field and water-maze learning/memory spared.<br><br>• NF immunostaining ↓ in molecular layer (p=0.043).<br><br>• Calbindin ↑ in Purkinje cells/dendrites (p=0.032).	• Granule-layer cell loss and cytotoxic changes
Koros C et al, 2009 [28]	Cytosine-arabinoside 400 mg/kg i.p. once daily for 5 days	Adult male Wistar rats, ~10 weeks old (≈ 250–300 g)	Vehicle control (0.9% NaCl i.p.)<br><br>Ara-C alone (400 mg/kg × 5 days i.p.)<br><br>NAC alone (200 mg/kg/day p.o. for 14 days)<br><br>Ara-C + NAC (NAC 7 days pre- and 5 days co-treatment)	n = 8 rats/group	High-dose Ara-C–induced cerebellar neurotoxicity via neurofilament (NF) alteration	To profile how Ara-C alters individual NF-H/M/L isoform expression in rat cerebellum and test whether the antioxidant NAC prevents NF loss in vivo	– Ara-C selectively reduced NF-H levels (≈ 40% drop vs. control; p = 0.006) with no significant change in NF-M or NF-L<br><br>– NAC co-treatment (Ara-C+NAC) preserved NF-H to control levels (no NF-H loss vs. NAC alone)<br><br>– IHC: Ara-C rats showed marked loss of NF immunoreactivity in molecular/axonal processes; NF-H loss confirmed by blot	– Reduced NF immunostaining in axons of molecular layer (basket/stellate axons & Purkinje dendrites)<br><br>– Unchanged NF in pinceau and Purkinje somata, consistent with selective NF-H degradation
Saif A-J et al, 2024 [50]	Cytarabine (Ara-C) 60 mg/kg i.p. once daily for 10 days total	New Zealand white rabbits (Oryctolagus cuniculus) ~4	A. Control: 1 mL 0.9% NaCl i.p.×10 d	n = 6 rabbits per group	Chemotherapy-induced parotid salivary gland injury	To characterize histological/immunohistochemical changes induced by Ara-	• Ara-C alone (Group C)<br>– Thickened connective-tissue trabeculae<br>– Acinar degeneration & necrosis, ductal necrosis	Serous acinar cell necrosis, ductal necrosis, stromal thickening, interlobular edema,

		months old, 1.75–2.25 kg	B. α-Tocopherol only: 800 IU p.o.×10 d<br><br>C. Ara-C only: 60 mg/kg i.p.×10 d<br><br>D. Ara-C+α-Tocopherol: 800 IU p.o. + 60 mg/kg i.p.×10 d			C in rabbit parotid glands and test whether α-tocopherol attenuates those lesions	– Inflammatory cell infiltrates (PMNs, giant cells)<br>– TNF-α score 2.7 ± 0.94 (grade 3) vs control ~0.5<br>– Bcl-2 score 0.4 ± 0.24 (grade 0) vs control ~2.2	atrophy and inflammatory infiltrates
Minden MD et al, 2024 [51]	Cytarabine 30 mg/kg i.p., twice daily on days 0–4	Male BALB/cAnNCRL mice, 12–18 weeks old (21–26 g)	Experiment 1 (cytarabine 30 mg/kg):<br><br>– Vehicle control (sterile saline, i.p.)<br><br>– Ara-C only<br><br>– Ara-C + apraglutide 3.3 mg/kg s.c. QD from day –4 to +12<br><br>– Ara-C + apraglutide 3.3 mg/kg s.c. from day 0 to +12	Experiment 1: n=6–18/group<br><br>Experiment 2 dose–response: n=16/group plus n=6 recovery animals	Chemotherapy-induced gastrointestinal mucosal injury and mortality	To evaluate whether the long-acting GLP-2 analog apraglutide protects the intestinal mucosa, preserves function and improves survival after cytarabine or melphalan chemotherapy, and after total-body irradiation plus allo-HSCT in mice	Chemotherapy model:<br><br>• Ara-C alone → 100% mortality by day 3, severe weight loss, villus atrophy, crypt damage, ↓citrulline, dysbiosis (↓Firmicutes, ↑Bacteroidetes)<br><br>• Apraglutide (3.3 mg/kg pre + co-) → 83–100% survival (vs 0% in Ara-C alone; p<0.0001), attenuated weight loss, preserved villi/ crypt architecture, maintained citrulline and normalized microbiota<br><br>Dose–response:	Villus shortening, crypt epithelial degeneration, lamina-propria inflammatory cell infiltrates, cryptal abscesses

			– Ara-C + apraglutide (3.3 mg/kg s.c.) on days –4, –2, 0, +3, +6, +9, +12 (+recovery cohorts for neutrophil counts)<br><br>Experiment 2 (cytarabine 30 mg/kg):<br><br>– Vehicle control<br><br>– Ara-C only<br><br>– Ara-C + apraglutide 0.11, 0.33, or 1.1 mg/kg s.c. QD from day –4 to +4<br><br>– Ara-C + hGLP-2 0.6 mg/kg s.c. BID from day –4 to +4				• Apraglutide 1.1 & 3.3 mg/kg → full or near-full survival vs Ara-C alone (p<0.01)<br><br>• Lower doses (0.33 mg/kg) less protective; hGLP-2 less effective	
Park M-R et al, 2023 [103]	Cytarabine (CYT) 100 mg/kg i.v. once daily ×	10-week-old male C57BL/6 mice (~25 g)	– CON: vehicle (saline)<br>– CYT: cytarabine	n ≈ 5–6 mice/group	Chemotherapy-induced cachexia (weight loss,	To dissect how cytarabine drives cachexia via energy-balance	• CYT mice ate as little as PF but lost more weight and fat/muscle mass than	• Intact epithelial tight junctions (no leaky gut); crypt/villus

	4 consecutive days		– PF: pair-fed to match CYT food intake + vehicle		muscle, fat wasting) and associated metabolic dysfunction	perturbation and to define the underlying mechanisms—specifically whether lipid malabsorption in the small intestine contributes independently of anorexia	PF → true cachexia (≥5 % BW loss)<br>• CYT suppressed nocturnal EE and Ucp1 expression in BAT/eWAT; raised nocturnal RQ vs. PF → lower lipid oxidation<br>• Serum TG ↓ in CYT vs. PF/CON; mucosal TG, cholesterol & FFA ↑ in duodenum/jejunum only in CYT<br>• Oral-fat-tolerance test: defective lipid absorption<br>• Fecal total lipids ≈ PF, but higher lipid per pellet + slowed small-bowel transit in CYT → retention in gut	morphology largely preserved<br>• TEM: enlarged chylomicrons (>600 nm) accumulated in TGN cisternae; secreted CMs trapped in intercellular space<br>• Lacteal LEC junctions shifted from “button-like” to impermeable “zipper-like” in CYT → blocked CM uptake<br>• Increased VE-cadherin zippering, with elevated VEGFR2(Y1175) & AKT(S473) phosphorylation
Kolure R et al, 2023 [71]	Cytarabine 25 mg/kg p.o. once daily × 13 days (gestation day 8–20)	Pregnant Sprague Dawley rats (~200 g at mating)	Control (0.9% NaCl p.o.)<br>Ara-C 25 mg/kg<br>Swertiamarin (ST) 100 mg/kg	n=8 rats/group	Cytarabine-induced hepatotoxicity (liver injury & oxidative stress) in the dam	To test whether the natural antioxidant swertiamarin protects against Ara-C–induced liver injury in pregnant rats	– Ara-C alone ↓ maternal weight gain vs. control ($p<0.01$)<br>– Ara-C ↑ MDA and serum AST/ALT, urea, creatinine; ↓ CAT, SOD, GSH, GSH-Px (all $p<0.01$)	Ara-C alone produced hepatic vacuolization, disrupted lobular architecture, pycnotic nuclei and dilated sinusoids.

https://doi.org/10.3390/cimb48010004

			ST 200 mg/kg<br><br>Ara-C + ST 100 mg/kg<br><br>Ara-C + ST 200 mg/kg					
Guan Z et al, 2023 [30]	Single i.p. injection on gestation day (GD) 7.5 at 5, 15, 22.5, 25 or 60 mg/kg body weight	Pregnant C57BL/6 mice, 7–8 weeks old, 18–20 g at mating	– Control (0 mg/kg)<br><br>– Ara-C 5 mg/kg<br><br>– Ara-C 15 mg/kg<br><br>– Ara-C 22.5 mg/kg<br><br>– Ara-C 25 mg/kg<br><br>– Ara-C 60 mg/kg	Exact number per group not stated	Neurodevelopmental toxicity, specifically neural tube defects (NTDs) in embryos	To characterize how maternal Ara-C exposure perturbs early embryonic neurodevelopment and triggers NTDs, and to define underlying cellular/molecular mechanisms	• Embryonic viability fell and growth-retardation and resorptions rose in a dose-dependent manner; 22.5 mg/kg gave the highest NTD rate (~30 % exencephaly, $P < 0.05$)<br><br>• Crown–rump length was significantly reduced in NTD embryos ($P < 0.05$) • PH3+ proliferation in neuroepithelium ↓ ~ 65 % ($P < 0.05$); cleaved caspase-3 apoptosis ↑ ~ 2–3-fold ($P < 0.05$)<br><br>• Nestin expression ↓ and GFAP expression ↑ at GD 13.5 ($P < 0.05$)—premature differentiation<br><br>• β-Catenin levels ↓ ~ 50–65 % on GD 11.5 and 13.5 ($P < 0.05$)—impaired Wnt signaling	• Exencephaly with failure of neural-tube closure<br><br>• Loose, swollen, disorganized neuroepithelial layer with irregular basal and luminal surfaces on HE sections

							• In mESC, Ara-C at 0.05 nM (environmental level) already reduced viability (CCK-8) and increased apoptosis; 3.75 µM Ara-C arrested cell-cycle in G1, lowered EdU incorporation and boosted EB formation (early differentiation)	
Salimi A et al, 2023 [31]	Cytarabine 70 mg/kg i.p. once daily for 5 days (days 10–14 of study)	Adult male Wistar rats, 200 ± 20 g	Vehicle control<br><br>Ara-C alone (70 mg/kg)<br><br>Betanin alone (25 mg/kg)<br><br>Vitamin D alone (500 U/kg)<br><br>Thymoquinone alone (0.5 mg/kg)<br><br>Ara-C + betanin (same doses)<br><br>Ara-C + vitamin D<br><br>Ara-C + thymoquinone	n = 6 rats per group	Cytarabine-induced neurotoxicity (oxidative/mitochondrial dysfunction in brain)	To test whether three mitoprotective/antioxidant agents (betanin, vitamin D, thymoquinone) can prevent Ara-C-driven mitochondrial impairment, oxidative stress, and histopathologic damage in rat brain	– Ara-C alone caused:<br>• ↓ AChE & BChE activities<br>• ↓ GSH; ↑ GSSG & MDA<br>• ↓ mitochondrial SDH activity<br>• ↑ mitochondrial swelling, ROS, MMP collapse<br>• Midbrain cell loss, cytoplasmic depletion, granule-layer fragmentation	Midbrain sections showed loss of neurons, cytoplasmic and nuclear depletion, and fragmentation of the granule cell layer on H&E

Khaleel B et al, 2022 [117]	Cytarabine 140 mg/kg i.p. every 12 h for three doses administered 24 h after AML cell injection	C57BL/6 male mice, 2 weeks old (sexually immature; ~10–12 g)	Untreated control<br><br>AML alone (i.p. C1498 cells)<br><br>CYT alone (140 mg/kg)<br><br>AML + CYT	n ≈ 6–12 per group	Long-term impairment of spermatogenesis and testicular histology	To determine how pediatric-equivalent AML and its standard chemotherapy (cytarabine) affect adult spermatogenic development when given before puberty	– AML engraftment raised monocyte ±30× and granulocyte ±10× counts and shortened mouse survival (~3 wk AML; ~5 wk AML+CYT vs. >6 wk controls)<br><br>– CYT alone ↓ testis weight; AML alone did not<br><br>– Both AML and CYT markedly ↑ seminiferous-tubule apoptosis (TUNEL) and ↓ "normal" tubule histology (↑ moderate/severe damage) at all time points<br><br>– SALL4/PLZF+ spermatogonia: unchanged by AML, ↑ with CYT alone<br><br>– CREM+ (meiotic) and acrosin+ (post-meiotic) cells: ↓ with AML, CYT, and AML+CYT vs. control; AML+CYT ≃ CYT<br><br>– PCNA+ spermatogonia: ↑ with AML, ↓ with CYT; AML+CYT intermediate	Seminiferous-tubule distortion: reduced germ-cell layers, luminal enlargement, cytoplasmic vacuolization, fragmented basement membrane

https://doi.org/10.3390/cimb48010004

							– Sperm: absent after CYT or CYT+AML; normal in controls<br><br>– Testicular GDNF, SCF, MCSF: ↓ protein with AML; GDNF & MCSF ↑ with CYT; SCF unaffected by CYT<br><br>– IL-6 mRNA: ↑ with AML, ↓ with CYT; IL-10 mRNA: ↓ with AML & CYT	
Saif A-J et al, 2020 [72]	Cytarabine 50 mg/kg BW, i.p., once daily × 7 days	New Zealand white rabbits, 3 months old, 1.5–2.0 kg	Control (1 mL sterile saline, i.p., daily × 7 d)<br><br>Ara-C alone (50 mg/kg i.p. daily × 7 d)<br><br>Vit E + Ara-C (Vitamin E 800 IU orally 5 h before each Ara-C dose, Ara-C as above)	n=6 rabbits/group	Cytarabine-induced hepatotoxicity	To characterize liver histopathology after Ara-C and to test Vitamin E's ability to prevent those lesions	– Ara-C alone caused:<br><br>• Mononuclear (and Kupffer/epithelioid) cell infiltration around central veins and portal areas<br><br>• Portal-area fibrosis and bile-duct hyperplasia<br><br>• Vascular congestion<br><br>• Hepatocyte coagulative necrosis<br><br>• Sinusoidal distension	– Central-vein area inflammatory-cell infiltrates<br><br>– Portal-area fibrosis with bile-duct hyperplasia<br><br>– Sinusoidal congestion/distension<br><br>– Coagulative necrosis of periportal hepatocytes
Dudina MO et al, 2018 [73]	Cytarabine 2 g/m2 i.v. once daily for 5 days	Wistar rats (both sexes), 180–220 g	Intact control (saline i.v. ×5 d)	n = 10/group	Acute cytotoxic drug-induced	To dissect molecular/cellular mechanisms of cytarabine	• Ara-C alone ↑ liver weight, lobular necrosis, vacuolization, steatosis, leukocyte infiltration,	• Central-lobular hepatocyte necrosis and karyolysis

https://doi.org/10.3390/cimb48010004

			CIADD (cytarabine alone)<br><br>CIADD + LBK-527 (magnesium 2-aminoethanesulfonate) 100 mg/kg p.o. 1 h before each Ara-C		liver damage (CIADD)	hepatotoxicity and test hepatoprotection by LBK-527	portal fibrosis, sinusoidal congestion; ↑ AST/ALT/GGTP/ALP (p<0.05); ↑ TNF-α; ↓ IL-10; ↑ HGF; ↑ Bcl-2; ↓ Ki-67 proliferation index	• Mononuclear/lymphohistiocytic infiltrates in lobules and portal tracts<br><br>• Hepatocellular vacuolar degeneration and steatosis<br><br>• Portal-area fibrosis and bile-duct hyperplasia<br><br>• Sinusoidal congestion and dilation
Porsani MYH et al, 2017 [52]	Cytarabine 15 mg/kg i.p. every 12 h for 4 doses on the last 2 days of a 21-day experiment	male Balb/C mice ~50 days old	• Control (no Ara-C)<br>• Ara-C only<br>• ß-glucan + Ara-C (80 mg/kg q24 h by gavage for 21 days)<br>• Glutamine + Ara-C (150 mg/kg q24 h by gavage for 21 days)<br>• ß-glucan + glutamine + Ara-C (same	n = 6 per group	Intestinal (villus/crypt architecture) and systemic immunological damage induced by cytarabine	To test whether oral pretreatment with soluble S. cerevisiae–derived ß-glucan, glutamine, or their combination protects mice from Ara-C–induced intestinal injury and immune suppression	Ara-C alone ↓ total leukocytes, ↓ IL-10, ↑ IFN-γ	untreated Ara-C mice showed reduced villus/crypt dimensions and WBC counts vs. controls

			doses/schedule)				
Takano T et al, 2006 [32]	Cytarabine 2 × 30 mg/kg i.p. to pregnant dams on gestational days 13.5 and 14.5	ICR mice (pregnant females and their offspring)	• Ara-C group: 12 pregnant dams (offspring analyzed) • Control group: 4 pregnant dams (saline) • For cell counts: n ≈ 3–5 offspring per group per time point	Developing-brain damage – neuronal apoptosis, disrupted migration, microcephaly & gray-matter heterotopia	To elucidate how prenatal Ara-C causes microcephaly and gray-matter heterotopia via disturbed neuronal migration	• E15: massive TUNEL+ apoptosis in VZ, IZ & GE; clusters of pyknotic neurons • ↓ nestin+ radial-glial fibers; disrupted BrdU labeling pattern in cingulate VZ • Loss/shortening of calretinin+ subplate fibers • P0–P3: bilateral subcortical heterotopia in cingulate/frontal cortex; later (P13) subependymal nodules • P32: overt microcephaly; enlarged subcortical heterotopia still confined to frontal/cingulate cortices • Heterotopic neurons express mature MAP-2 isoforms, indicating neocortical origin	• Widespread apoptosis in proliferative zones (VZ/IZ) • Atrophy of ganglionic eminence • Disorganized cortical lamination over heterotopia • Failure of radial and tangential migratory scaffolds
Elli M et al, 2009 [53]	Cytarabine 3.6 mg per mouse i.p. once daily × 5 days on days 3–7 of a 7-day protocol	Male BALB/c mice, 8–10 weeks old, 27–35 g	I. Control (saline only) II. ARA-C + saline III. ARA-C + vitamin A (5000 IU/kg p.o. daily ×7 d)	n≈5 each	ARA-C–induced small-intestinal mucosal injury (villus atrophy, enterocyte loss,	To test whether oral vitamin A protects against Ara-C–induced jejunal mucosal damage	• ARA-C alone → significant villus shortening, enterocyte loss & inflammation	• Marked villus atrophy with blunt, shortened villi • Crypt hyperplasia & inflammatory cell infiltrate

https://doi.org/10.3390/cimb48010004

			IV. ARA-C + lipid vehicle<br>V. Vitamin A alone		inflammation<br>)			
Sun B et al ,2009 [67]	Cytarabine 30 mg/kg/day i.p. once daily for 7 consecutive days	Sprague–Dawley rats (neonatal pups) 8 days old	• Group 1 (saline i.p. only): n = 3<br>• Group 2 (Ara-C alone): n = 7<br>• Group 3 (Ara-C + AHCC p.o., 500 mg/kg/day): n = 9<br>• Group 4 (Ara-C + AHCC i.p., 500 mg/kg/day): n = 10<br>• Group 5 (Ara-C + AHCC topical swab, 5% solution): n = 10		Chemotherapy-induced alopecia (hair-loss) and follicle damage	To test whether co-treatment with AHCC can protect neonatal rats from cytosine-arabinoside–induced hair-loss	• Ara-C alone: 71.4% of pups had severe alopecia (75–100% hair loss), 28.6% moderate (25–75%)	Profound loss of hair-follicle number and atrophy of follicles in Ara-C–treated skin fields
Diets-Ouwehand J et al, 1992 [60]	Intravitreal, fractionated doses of Cytarabine in one eye; fellow eye received saline	Pigmented chinchilla rabbits (2.3–3.2 kg)	• Each animal: one eye injected with Ara-C, fellow eye with saline<br><br>• Dose subsets: 600 µg (n = 3 eyes), 1 500 µg (n = 3), 2 700 µg (n = 1)<br>– 600 µg total: 2 × 300 µg on consecutive days (24 h apart)<br><br>– 1,500 µg total: 5 × 300 µg on 5	n = 7 rabbits	Retinal and blood–retina–barrier toxicity following intravitreal Ara-C	To define the retinal safety profile of intravitreal cytarabine for potential local treatment of vitreoretinal non-Hodgkin lymphoma	• 600 µg: no significant biomicroscopic, ERG, fluorophotometric, or histologic changes<br>• ≥ 1,500 µg: transient increase in fluorescein leakage (blood–retina–barrier impairment) that normalized in ~2 weeks; irreversible widening/reduction of scotopic b-wave and prolonged latency; persistent vascular narrowing & retinal pallor on exam	• Light microscopy: no overt morphologic changes at any dose<br><br>• Electron microscopy at ≥ 1,500 µg: disorganization of synaptic-ribbon vesicle lining in rod photoreceptor pedicles; occasional vacuoles & enlarged vesicles

			consecutive days<br><br>– 2,700 μg total: 3 × 900 μg on 3 consecutive days					
Watanabe S et al, 1992 [118]	1-β-d-Arabinosylcytosine (ara-C) s.c. at 63 mg/kg body weight once daily for 5 days	Male Sprague–Dawley rats, 50 days old, 170–180 g	• ara-C group: n = 6 rats, 63 mg/kg/day × 5 days<br>• Control group: n = 6 rats, saline s.c. × 5 days	n = 6 per group	Systemic/cellular effects of ara-C on male accessory organs (prostate, seminal vesicles, testis)	To determine how a 5-day ara-C course alters body/organ weights and polyamine levels (putrescine, spermidine, spermine) in male rat accessory glands	• Body weight fell 13% in ara-C rats (P < 0.01)<br><br>• Prostate weight down 20% (P < 0.05); seminal vesicles down 13%; testes down 5% (P < 0.1)<br><br>• Per-g organ polyamine rises in ara-C group: prostate put × 1.2, spd × 1.8 (P < 0.01), spm × 2.9 (P < 0.01); seminal vesicle put/spd/spm each +40%; testes spd and spm +20% (P < 0.05)<br><br>• Total organ polyamines significantly higher in prostate and testis despite weight loss<br><br>• spd/spm ratio fell in prostate only	N/A
Yamauchi H et al, 2004 [33]	Cytarabine (Ara-C) 100 mg/kg	Timed-pregnant Slc:Wistar rats; Dams ~200–250 g;	• Ara-C–treated:	n = 6 per group	Ara-C–induced fetal brain	To define the role of p53 and its downstream	• Apoptosis index rose from 3 h, peaked at 9–12 h, normalized by 48 h	• Marked apoptosis of neuroepithelial

	i.p. once daily for 7 days	fetuses collected at embryonic day 14 (GD13 + 1 day)	• Vehicle (PBS) controls	injury—neuroepithelial apoptosis & cell-cycle arrest	targets in mediating Ara-C–induced apoptosis and mitotic inhibition in the fetal rat brain	• Mitotic index fell sharply at 3–12 h, recovered by 48 h<br>• p53 protein labeling peaked at 3 h; p21 protein peaked at 6 h (both returned to baseline by 48 h)<br>• p21, bax, cyclin G1 & fas mRNAs all up-regulated after p53 induction (p21 from 3–24 h; others ~9 h peak)	cells in ventricular zone<br>• Pronounced suppression of neuroepithelial mitoses
Percy DH et al, 1977 [61]	Cytarabine 15 mg/kg/d s.c. injection once daily on postnatal days 1–5	Newborn Sprague–Dawley rats	• ara-C–treated: n = 52 pups<br>• saline-injected controls: n = 18 littermates	Developmental/teratogenic effects of ara-C on postnatal retinal maturation (“retinal dysplasia”)	To chart the long-term histologic changes in the rat retina—rosette formation, photoreceptor/bipolar layer disruption—after neonatal ara-C	• Growth retardation, tremor/ataxia & visual impairment in treated pups<br>• By day 6: reactive pigment epithelium, separation of neuroretina, abundant rosettes bounded by an external limiting membrane<br>• Bipolar-cell nuclei scattered into the outer nuclear layer; misalignment of photoreceptor processes	• Numerous photoreceptor-lined rosettes containing cell debris and disordered outer segments<br>• Displacement of bipolar- and Müller-cell nuclei into the photoreceptor layer<br>• Reactive/villous pigment epithelium; chronic degeneration with

https://doi.org/10.3390/cimb48010004

							• Persistent retinal thinning, cellular degeneration & phagocyte infiltration at 30–60 days	macrophages at later ages
Shimada M et al, 1975 [34]	Cytarabine 30 mg/kg/d or 50 mg/kg/d SC injections on postnatal days 2, 3 and 4 (three consecutive days)	Newborn ICR-JCL mice ("suckling" pups)	• Group 1 (30 mg/kg): n = 82 pups<br>• Group 2 (50 mg/kg): n = 112 pups<br>• Controls (saline): n = 35 pups	n = 229 total	Disruption of postnatal cerebellar development—external granular layer (EGL) cytotoxicity and resultant dysplasia	To characterize the time course and histopathology of ara-C–induced injury, necrosis and regeneration in the neonatal mouse cerebellum	• Growth: both doses → transient weight stunting (30 d weights 18.5 ± 1.2 g at 30 mg/kg; 10.3 ± 1.1 g at 50 mg/kg vs 20.7 ± 0.3 g controls)<br><br>• 30 mg/kg: no mortality, no ataxia; 50 mg/kg: 10/112 deaths, ataxia after day 15<br><br>• Day 5 (24 h post-last dose): massive necrosis of undifferentiated EGL cells; scant survivors at 30 mg/kg, none at 50 mg/kg<br><br>• Days 7–15: partial, regional regeneration of EGL—more robust in posterior lobes, incomplete overall<br><br>• Autoradiography: regenerating EGL draws ^{3}H-label but migration is disordered, with EGL–IGL misplacements	• Acute EGL cell necrosis<br>• Incomplete/regional EGL regeneration<br>• Heterotopic (misplaced) granule cells in molecular layer<br>• Disorganized Purkinje cell layer<br>• Reduced total cerebellar size (vermis & overall width)

							• By day 20: EGL normally gone but treated mice show heterotopic granule cells in molecular layer, irregular Purkinje cell rows, hypocellular IGL<br><br>• Day 30: persistent cerebellar hypoplasia (vermian length down ~25% at 30 mg/kg; ~50% at 50 mg/kg) and marked dysplasia	
Ramos MG et al, 1999 [48]	3.6 mg Ara-C per mouse per day, given intraperitoneally for the last 2 days of a 9-day protocol	Germ-free mice (n = 30 total)	– SCFA/O + Ara-C<br>– SCFA/IG + Ara-C<br>– Placebo/O + Ara-C<br>– Placebo/IG + Ara-C<br><br>• 3 control groups<br>– SCFA/O + Saline<br>– SCFA/IG + Saline<br>– Normal (water+diet)	Total mice = 30	Ara-C-induced intestinal mucosal damage (villus atrophy, enterocyte loss, necrosis, inflammation)	To determine whether orally or intragastrically administered short-chain fatty acids (SCFA) protect germ-free mouse intestinal mucosa from Ara-C–induced lesions, independent of endogenous SCFA production	Ara-C alone → severe villus shortening, enterocyte loss, necrosis, inflammation	• Shortened, blunted/fused villi<br>• Reduced enterocyte number<br>• Submucosal inflammatory infiltrate<br>• Foci of necrosis
Hussein AM, 1995 [68]	75 mg/kg/day cytosine arabinoside, intraperitoneal	8-day-old Sprague–Dawley rat pups	Ara-C + vehicle control<br><br>Ara-C + topical	n = 78 total; 13 rats/group	Chemotherapy-induced alopecia (hair-follicle	To test whether minoxidil (topical or subcutaneous)	Ara-C + vehicle or topical Rogaine → 100% complete alopecia in treated area	N/A

https://doi.org/10.3390/cimb48010004

	injections (0.1 mL) once daily for 5 consecutive days		minoxidil (2% Rogaine)<br><br>Ara-C + subcutaneous minoxidil (2 mg/mL, 0.1 mL)<br><br>Cyclophosphamide (50 mg/kg × 1 IP) + vehicle control<br><br>Cyclophosphamide + topical minoxidil<br><br>Cyclophosphamide + subcutaneous minoxidil	arrest leading to total hair loss)	can prevent Ara-C–induced alopecia in newborn rats		
Percy DH et al, 1974 [35]	Cytosine arabinoside (CA) 3.125–50 mg/kg/day, subcutaneously for 5 consecutive days	ICR Swiss albino mice and Sprague–Dawley rats	For each species, treatments at five dose levels (50, 25, 12.5, 6.25, 3.125 mg/kg/day), begun at P1, P5 or P10. Rough group sizes (initially treated) at P1:<br>• Mice: n = 20, 21, 13, 6, 6 (for the five doses) + 26 untreated littermates<br>• Rats: n = 30, 28, 10, 10, 10 + 35 untreated littermates	Postnatal developmental defects in CNS (cerebellum), retina and kidney	To characterize age- and dose-dependent developmental lesions induced by postnatal CA in newborn rodents	– Mortality and growth retardation were highest when CA began at P1 and at ≥ 25 mg/kg/day<br><br>– Lesion severity decreased sharply when dosing began at P5, and was minimal at P10<br><br>– Autoradiography confirmed intense postnatal cell division in	Cerebellum:<br><br>– Marked hypoplasia, folial shortening, loss of normal lamination (molecular/Purkinje/internal granular layers poorly defined), scattered Purkinje & granule cells, vacuoles in cortex

https://doi.org/10.3390/cimb48010004

						cerebellum, retinal nuclear layers and renal cortex, correlating with windows of maximal CA sensitivity	Retina:<br><br>– Retinal dysplasia with numerous rosettes in peripheral outer nuclear layer (rosette lumen lined by photoreceptors), thinning of nuclear layers, pigment-epithelium irregularities, optic-nerve hypoplasia<br><br>Kidney:<br><br>– Focal cortical dysplasia: subcapsular nests of undifferentiated “primordial” renal cells, arrested glomerular & tubular development
Yamano T et al, 1983 [44]	Cytosine arabinoside 30 mg/kg s.c. once daily for 3 days	ICR-JCL strain suckling mice	Four timing schedules:<br><br>– Group I: postnatal days 2–4	n = 45 total pups divided into five groups (approx. 9 mice/group)	Loss of external granular layer (EGL) and ensuing cerebellar	To define how the postnatal timing of EGL destruction by Ara-C determines later	• P20: Groups I–III retained an EGL (most extensive in I > II > III); Group IV and controls had virtually no EGL.<br><br>• P30 histology:

			– Group II: days 4–6<br>– Group III: days 7–9<br>– Group IV: days 10–12<br>Control (saline on P2–4)		maldevelopment	cerebellar cytoarchitectural abnormalities	– Group I: widespread Purkinje-cell disarray + heterotopic granule cells in all lobes<br>– Group II: heterotopics in every lobe; Purkinje disarray only in anterior/middle lobes<br>– Group III: heterotopics limited to anterior/middle lobes; Purkinje organization preserved<br>– Group IV & control: normal cytoarchitecture<br>• Golgi-Cox: four abnormal Purkinje-cell morphologies (inverted, lying, T-shaped, poorly arborized); severity inversely proportional to age at dosing (I > II > III > IV)<br>• EM: ectopic glomerular synaptic complexes and "naked" Purkinje-cell spines in molecular layer of I–III	
Percy DH, 1975 [36]	Cytosine arabinoside (CA) 12.5, 25 or 50 mg/kg·day – SC injections on three consecutive days of late gestation	ICR Swiss albino mice and Sprague–Dawley rats	– CA 12.5 mg/kg·day × 3 days<br>– CA 25 mg/kg·day × 3 days<br>– CA 50 mg/kg·day × 3 days<br>– Untreated controls	Numbers per group not stated	Teratogenic developmental defects in cerebellum, retina, and kidney	To characterize the dose-dependent teratogenic effects of prenatal CA on late-fetal development of brain, eye, and kidney in mice and rats	– CA at 50 mg/kg·day → segmental cerebellar hypoplasia (most severe in anterior vermian lobules 1–3), focal renal cortical microcysts, and retinal dysplasia in rats<br>– Mice exposed to 50 mg/kg·day → similar cerebellar and renal lesions but no consistent retinal dysplasia	– Cerebellum: reduced folial size/tortuosity, poorly demarcated molecular / Purkinje / internal granular layers, persistent external granular layer in hypoplastic lobules<br>– Retina (rats at 50 mg/kg): central

						– Lower CA doses (25, 12.5 mg/kg·day) → milder or absent defects<br>– Lesion distribution corresponded to developmental stage of each organ at time of exposure	retinal thinning with rosette formation of photoreceptors + outer nuclear layer, inner-layer undulations<br>– Kidney: focal subcapsular microcysts—dilated tubules lined by vacuolated epithelium; nests of primordial cells indicating arrested nephrogenesis
Shimada M et al, 1973 [62]	Cytosine arabinoside s.c. at 30 mg/kg once daily on postnatal days 2, 3 and 4 (“group 1”) – Parallel cohort at 50 mg/kg × 3 days (“group 2”)	ICR-JCL suckling mice	– Group 1 (30 mg/kg × 3): n = 47 pups<br>– Group 2 (50 mg/kg × 3): n = 74 pups<br>– Controls: age-matched untreated littermates	Disruption of normal retinal histogenesis → rosette formation and heterotopic ganglion-cell placement	To determine whether postnatal cytosine arabinoside induces rosette-type dysplasia in the developing mouse retina	– 24 h after the final dose (day 5): selective necrosis of the undifferentiated (outer) nuclear layer with nuclear debris and cavitation<br>– By day 7: debris cleared but widespread rosette formation in the outer nuclear layer; occasional mitoses persisted at the inner edge of the former undifferentiated layer<br>– Persistence of rosettes through days 10–30:	– Rosette-type infoldings of photoreceptor/outer-nuclear–layer cells into an ectopic lumen<br>– Persistent heterotopic ganglion cells within the inner plexiform layer<br>– Selective loss and failed differentiation of the postmitotic

https://doi.org/10.3390/cimb48010004

							• Group 1 (30 mg/kg): 81% of retinas still showed rosettes at day 20<br><br>• Group 2 (50 mg/kg): 100% incidence at day 20 and day 30<br><br>– Inner plexiform layer widened ~2× normal in treated pups; heterotopic ganglion-cell clusters appeared within it	(future inner nuclear) zone
Guzmán DC et al, 2024 [37]	Cytarabine 0.08 mM, single intraperitoneal injection	Female Wistar rats, 4 weeks old (~70 g)	Saline control<br><br>Cytarabine alone (0.08 mM IP)<br><br>Etoposide alone (0.007 mM IP)<br><br>Oligoelements mix alone (50 µL IP)<br><br>Oligoelements + cytarabine<br><br>Oligoelements + etoposide	n = 6 rats per group; total 36	Cytarabine- or etoposide-induced oxidative stress and neurochemical disruption in brain regions	To determine how co-administration of a trace-element (“oligoelement”) mix modulates cytarabine- or etoposide-induced changes in dopamine metabolism and markers of oxidative damage across cortex, striatum, and cerebellum/medulla	• DA<br>– OE + cytarabine: ↑ striatal DA vs. control ($p < 0.0002$)<br>– OE + cytarabine: ↓ cerebellum/medulla DA vs. control ($p < 0.005$)<br><br>• GSH<br>– Cytarabine alone: ↑ cortical GSH vs. control ($p < 0.05$)<br>– OE + etoposide: ↓ cortical GSH vs. etoposide alone ($p < 0.006$)<br><br>• TBARS<br>– Cytarabine and OE + etoposide: ↓ lipid peroxidation in cortex, striatum, cerebellum ($p < 0.02$)	N/A

https://doi.org/10.3390/cimb48010004

							• Na^+/K^+-ATPase<br>– Cytarabine and OE + cytarabine: ↑ activity in striatum and cerebellum/medulla vs. control ($p < 0.008$)	
Narang HK, 1982 [38]	Four cytarabine (Ara-C) schedules, all beginning on day 4 after HSV-1 inoculation:<br><br>– 20 mg/kg day SC once daily for 5 days<br><br>– 10 mg/kg day SC in two 5 mg/kg doses daily for 5 days<br><br>– 10 mg/kg day SC once daily for 5 days, drug-free 5 days, then 5 days on<br><br>– 20 mg/kg SC on alternate days for 10 days	New Zealand albino rabbits, 18 days old at HSV inoculation (~35–50 g)	• Ara-C arms: 4 schedules × 10 rabbits each (n = 40)<br><br>• Ara-A arms: 4 dosing schedules × 10 each (n = 40)<br><br>• Untreated HSV controls: 20 rabbits<br><br>• Uninoculated drug-only toxicity controls: 12 rabbits (6 mg/kg Ara-A or 20 mg/kg Ara-C for 5–10 days)	each cohort ≈10 litter-mates	Cytarabine's systemic toxicity & its effect on survival, neurologic signs, and CNS pathology in HSV encephalitis	To compare pathological (histological + ultrastructural) lesions and survival in HSV-1–infected rabbits treated with Ara-A versus Ara-C	• Mortality during treatment: Ara-C 30–50% vs untreated 10% (dose-dependent)<br>• Early (day 5–10): treated animals showed fewer macrophages in lesions than controls<br>• Late (≥ day 15): Ara-C (and Ara-A) markedly worsened optic-nerve atrophy vs controls<br>• Neurologic signs (ataxia, head jerks, convulsions) persisted or were delayed but not prevented in Ara-C group	• Right optic nerve/chiasm: gross atrophy, meningeal thickening<br><br>• Spongy degeneration with astrocyte/oligodendrocyte loss<br><br>• Large extracellular vacuoles filled with lipid-laden macrophages<br><br>• Endothelial hyperplasia, vessel wall thickening, remyelination attempts<br><br>• HSV particles persisting in neuroglia on EM

https://doi.org/10.3390/cimb48010004

Yamauchi H et al, 2003 [83]	Cytarabine 250 mg/kg single i.p. injection on gestational day 13 (GD 13)	Pregnant Wistar rat	• Ara-C group: 18 pregnant dams<br>• Control group (PBS vehicle): 18 pregnant dams	• Ara-C–induced apoptosis (fetal and placental cytotoxicity)<br>• Associated fetotoxic/teratogenic effects (growth inhibition, malformations)	To characterize the time course and distribution of Ara-C–induced apoptosis in rat fetal tissues and placenta, and relate it to fetotoxicity/teratogenicity	• Fetal and placental weights significantly ↓ at 48 h post-dose.<br>• Labyrinth-zone thickness of placenta ↓ by 48 h.<br>• Marked ↑ in pyknotic (TUNEL-positive) cells in<br>– CNS neuroepithelium (outer ventricular zone)<br>– Mesenchymal tissues (craniofacial, limb bud, tail bud)<br>– Hematopoietic liver progenitors, spinal ganglia, alimentary tract, lung, gonads<br>– Placental labyrinth trophoblasts<br>• Onset: 3–6 h; peak apoptosis: 6 h in placenta, 9–12 h in fetal tissues; still above control at 48 h.<br>• Electron microscopy confirmed classic apoptotic features (chromatin condensation, nuclear fragmentation, phagocytosis of apoptotic bodies)	• Fetal hypoplasia<br>• Reduced thickness of placental labyrinth zone<br>• Widespread apoptotic cell death (pyknosis, DNA fragmentation) in proliferative fetal and trophoblastic compartments

Matsutani T et al, 1983 [39]	Fetal administration (FA-group) Single i.p. injection of 280 mg/kg on gestational day 15 (GD 15)<br><br>Neonatal administration (NA-group) 30 mg/kg/day s.c. on postnatal days 4–7 (4 consecutive days)	Wistar-Imamichi albino rats 347 ± 11 g	Fetal administration • Ara-C – offspring n=5–8 per assay • Saline controls – n=7 per assay<br><br>Neonatal administration • Ara-C – offspring n=3–11 per assay • Saline controls – n=3–11 per assay	Developmental impairment of the brain (growth retardation, hypoplasia) and associated neurochemical changes	To characterize long-term neurochemical alterations (monoamines, DNA content, myelin markers) in rat brain following Ara-C–induced developmental impairment in utero or neonatally	– FA-group (single 280 mg/kg at GD 15):<br><br>• Body weight ↓ ~15% vs control at day 60<br><br>• Cerebral hemisphere weight ↓ to 60% of control; brain-stem to 75%; cerebellum to 89%<br><br>• DNA per region ↓ to ~63% (cerebrum); DNA per gram tissue unchanged • NE, DA, 5-HT concentrations (µg/g) in cerebrum ↑ ~1.7–1.8×; smaller ↑ in brain-stem & pons-medulla; no change in cerebellum • CNPase activity & myelin protein unchanged in cerebrum/brain-stem; slight ↓ in cerebellum<br><br>– NA-group (4 × 30 mg/kg P4–7): • Body weight ↓ ~50%; pronounced ataxia, delayed hairing • Cerebellum weight ↓ to ~33% of control; cerebrum & brain-stem to ~90%	Gross cerebellar hypoplasia, post-weaning ataxia, delayed hair appearance; no detailed light-microscopic histology reported

						• DNA per cerebellum ↓ to 9% of control; DNA per gram cerebellum ↓ to ~29%<br>• NE & 5-HT in cerebellum ↑ ~3–4× (µg/g); total monoamine per region unchanged<br>• Cerebellar CNPase activity ↑ 1.36×; myelin protein ↑ 1.7× (relative myelin enrichment)<br>• CNPase & myelin protein in cerebrum/brain-stem slightly ↓ vs control	
Chwalinski S et al, 1989 [54]	1-β-D-arabinofuranosylcytosine 1st dose: 200 µg/g bw i.p. ; 2nd dose: 100 µg/g bw i.p. 12 h later	Male BDF1 (BDF) mice 10–12 weeks old, ~25 g	Crypt base/Paneth cell counts: 19 mice (≈520 crypts)<br><br>Label- and mitotic-index studies (3H-TdR autoradiography): 10 mice<br><br>3H-TdR cytotoxicity time-course (6, 12, 24 h post-label): 4 mice per time point<br><br>Ara-C regeneration time-course (1, 6, 12, 18 h after 2nd dose): 4 mice per time point	S-phase–specific cytotoxic damage to proliferating crypt cells in ileal crypts	To determine how crypt-base columnar cells (BCC) and Paneth cells (PC) contribute to crypt regeneration following S-phase–specific damage by Ara-C, and to map the spatial pattern of proliferation and injury within the bottom (“stem-cell”) zone of the crypt	Ara-C injury & regeneration:<br>• 6 h after 2nd dose: widespread destruction of cells above PC zone, PC themselves spared<br>• 12–18 h: first 3H-TdR uptake reappeared in BCC and immediately overlying cells—peak LI at positions 4–5 during regeneration (≈ 40% of control labelling)<br>• Regeneration clearly initiates at crypt base (PC/BCC zone)	Selective loss of proliferative crypt cells above Paneth zone; Paneth cells morphologically intact<br><br>Crypts reduced to surviving PC/BCC layer by 6 h post-treatment<br><br>By 12–18 h, re-emergence of S-phase cells first within PC zone

https://doi.org/10.3390/cimb48010004

Kaufman HE et al, 1964 [63]	Rabbit model: 1.0% CA ophthalmic drops administered to one eye every 2 h around the clock for 5 days	New Zealand white rabbits, one eye treated, fellow eye as intra-animal control	– Placebo vehicle: 12 eyes<br>– 0.1% idoxuridine: 12 eyes<br>– 1.0% CA alone: 36 eyes<br>– 1.0% CA + 100 µg deoxycytidine: 20 eyes<br>– 1.0% CA + 200 mg deoxycytidine: 16 eyes	Corneal epithelial toxicity: superficial opacities (“glittering” deposits), punctate epithelial staining, pain/iritis	To characterize the corneal toxicity of topical cytosine arabinoside and determine whether it stems solely from DNA-synthesis inhibition or involves other metabolic effects	– In rabbits 1.0% CA → “glittering” epithelial opacities in 92% of treated eyes; idoxuridine or vehicle → no epithelial toxicity<br><br>– Autoradiography: CA (but not idoxuridine) profoundly suppressed ^3H-thymidine uptake in corneal epithelium (and stroma); idoxuridine only partially inhibited stromal DNA synthesis<br><br>– Enzyme histochemistry: CA caused loss of glycolytic enzymes (lactic and α-glycerophosphate dehydrogenase) in superficial epithelial layers; other dehydrogenases preserved	– Megalocytic (“megaloblastic”) epithelial cells, especially in basal layers<br><br>– Fragmented or weakened Feulgen (DNA) staining in central epithelium<br><br>– Loss of superficial glycolytic enzyme activity
Yamano T et al, 1980 [40]	Cytarabine 30 mg/kg s.c. once daily on postnatal days 2, 3 and 4	ICR-JCL mouse pups	N = 24 pups total	Disruption of external granular layer leading to aberrant cerebellar development (heterotopic granule cells, Purkinje cell	To define how neonatal loss of the external granular layer (via Ara-C) leads to formation of heterotopic granule cells in the molecular layer	– Ara-C ablated the external granular layer (EGL) in the first postnatal week; a regenerating EGL re-emerged beginning ~P10<br><br>– By P10, mossy fibers and climbing-fiber collaterals aberrantly	– P30 cerebella: scattered granule cells in the inner ML, rosette-like clusters, disorganized Purkinje cell layer, depleted IGL cell density

					disarray, reduced inner granular layer)		extended into the molecular layer (ML) in treated but not control mice<br>– Migrating granule cells in the ML formed synapses onto these misguided axons, never reaching inner granular layer (IGL) → stable heterotopic granule cells by P30 (often in rosettes)<br>– Autoradiography showed ³H-thymidine–labeled heterotopic granule cells in ML at P30; controls had labeled cells only in IGL	– EM: glomerular synapses between ectopic granule-cell dendrites and mossy-fiber/ climbing-fiber axons in ML; reduction of parallel fibers; unattached Purkinje spines
Hagiwara S et al, 2011 [69]	Cytosine arabinoside (AraC) 20 mg/kg day–1 by intraperitoneal injection for 7 consecutive days (days 0–6)	Wistar rat pups, 8 days old at first dose	• Saline + vehicle cream (control)<br>• AraC + 0% DHLHZn cream<br>• AraC + 0.5% DHLHZn cream<br>• AraC + 1% DHLHZn cream – AraC	n = 10 rats per group	Chemotherapy-induced alopecia (hair loss)	To test whether topical sodium zinc dihydrolipoylhistidinate (DHLHZn), an α-lipoic acid derivative antioxidant, prevents AraC-induced hair loss	– AraC + vehicle → complete alopecia (score ≈ 0)	– Hair follicles with dense inflammatory infiltrates<br>– Electron-microscopy: swollen mitochondria, disrupted cristae, flocculent matrix in hair-matrix cells

https://doi.org/10.3390/cimb48010004

			(or saline) on days 0–6; topical cream daily on days 0–12					
Orth JM et al, 1988 [119]	cytosine arabinoside (araC) 60 mg/ml solution, 2 µl/testis × bilateral intratesticular injections at 36–48 h after birth	Sprague-Dawley rat pups 36–48 h after birth (day 2)	• araC-treated: n = 4 adults for morphometry; n = 3 per time-point for short-term studies<br>• saline-injected controls: same n as araC group	n = 4 rats per group	Selective depletion of Sertoli cell proliferation leading to reduced adult Sertoli-cell population	To test whether adult spermatid output depends quantitatively on the size of the Sertoli-cell population established during perinatal development	• Short term: araC sharply reduced Sertoli-cell S-phase labeling by 24 h; germ-cell labeling and timing of onset were unaffected<br>• Adult Sertoli cell number: 15.5 × 10^6 in controls vs. 7.1 × 10^6 in araC-treated (–54%; $P < 0.01$)<br>• Round spermatids per testis: 92.2 × 10^6 in controls vs. 41.1 × 10^6 in araC-treated (–55%; $P < 0.01$)<br>• Spermatids per Sertoli cell: ~5.7–6.0 in both groups (no difference)<br>• Serum ABP: halved in araC-treated vs. control ($P < 0.01$), but ABP output per Sertoli cell unchanged<br>• Serum FSH: doubled in araC-treated vs. control ($P < 0.001$)<br>• Leydig cell volume and ventral prostate weight: no difference vs. controls	• Neonatal araC abolished Sertoli-cell proliferation without overt tubular necrosis<br>• Adult seminiferous tubules: normal histological architecture and complete spermatogenesis but reduced tubular volume consistent with fewer Sertoli cells
Wang J et al, 2018 [78]	Cytarabine (Ara-C) 250 mg/kg i.p. once daily	Male C57BL/6 mice, 18–20 g	1. Saline control	n = 10 mice per group	Chemotherapy-induced	To test whether lienal peptide (LP) can reverse	N/A	N/A

	for 3 days (days 0–2)		2. Ara-C only (immunosuppressed model)<br>3. Ara-C + LP 1.5 mg/kg/day i.p.<br>4. Ara-C + LP 4.5 mg/kg/day i.p.	immunosuppression by Ara-C	Ara-C–induced immune suppression in vitro and in vivo		
Han S et al, 2023 [55]	Cytarabine (Ara-C) was added to the standard Drosophila diet at final concentrations of 0, 1, 5 or 10 mM. – Flies were exposed continuously from egg lay, through larval and pupal stages, into adulthood (≥ 10 days post-eclosion)	Drosophila melanogaster (w1118 strain and reporter lines)	– Development assays: ~100 eggs/vial, 3 vials × 3 independent replicates<br>– Adult assays (lifespan, climbing, feeding, stress): 20 flies/vial, n = 5–8 vials per dose<br>– Biochemical & histological assays: typically n = 3–10 biological replicates per dose	– Developmental delay & pupal defects<br>– Adult lifespan shortening, locomotor impairment<br>– Reduced feeding, diminished stress resistance (oxidative, starvation, SDS)<br>– Intestinal morphological and ultrastructural damage	To establish a rapid in vivo Drosophila model for Ara-C–induced side effects and dissect the underlying mechanisms, particularly intestinal injury	– 10 mM Ara-C delayed pupation, reduced pupa number & size, cut adult eclosion rates by > 50%<br>– Adult flies fed Ara-C (1–10 mM) showed dose-dependent lifespan shortening and impaired climbing<br>– Feeding was suppressed and female flies lost starvation resistance after Ara-C; males less affected<br>– Ara-C increased mortality under H_2O_2 and SDS stress; elevated GSTD1/2 expression but little change in SOD/CAT mRNA<br>– Gut length and epithelial thickness were reduced; TEM revealed epithelial cell edema, microvilli truncation, swollen mitochondria	Midgut epithelial disruption: sparse/truncated microvilli, nuclear repositioning, mitochondrial swelling and membrane rupture

						– Ara-C doubled ISC mitoses (anti-pH3) and increased esg>GFP+ ISCs/EBs; elevated gut ROS (DHE, gstD1-GFP)<br>– Toll and IMD innate-immune pathway genes and antimicrobial peptides were upregulated in Ara-C guts<br>– Pro-apoptotic genes (reaper, drice, dcp-1) were induced in the gut	
Chen T, 1982 [56]	1-β-D-arabinofuranosylcytosine (ara-C) 50 mg/kg bodyweight, intraperitoneal once daily × 5 consecutive days	Male Swiss-Webster mice 30–40 g at start of dosing	– Control (saline) n≈11<br>– ara-C (50 mg/kg × 5 d) n≈10–11<br>– 5-aza-C (15 mg/kg × 5 d) n≈6<br>– ara-C + 2′-deoxycytidine (100 mg/kg ip) n≈9 – 5-aza-C + cytidine (50 mg/kg ip) n≈6	Impairment of active intestinal absorption: glucose, 3-O-methyl-glucose, amino acids, Na^+ and Cl^-	To determine whether the pyrimidine antimetabolites ara-C and 5-aza-C disrupt intestinal nutrient and electrolyte transport, and to test if co-administration of endogenous nucleosides prevents these effects	• ara-C in vivo (50 mg/kg × 5 d) decreased D-glucose, 3MG and L-tyrosine transport by 67%, 74% and 60%, respectively; 5-aza-C (15 mg/kg × 5 d) reduced these by 58%, 79% and 62%.<br>• Fasting (24 h, 17% weight loss) ↑ nutrient transport (234–350% of control), ruling out malnutrition as cause.	N/A
Yamauchi H et al, 2004 [100]	250 mg/kg 1-β-D-arabinofuranosylcytosine i.p., single dose on gestation Day 13	Slc:Wistar pregnant rats, gestation Day 13	– Ara-C group: six dams sacrificed at each time point post-dose (1, 3, 6, 9, 12, 24, 48 h)<br>– Control group: six PBS-treated dams per time point	Placental injury: trophoblast apoptosis and impaired proliferation	To elucidate mechanisms of Ara-C-induced placental cytotoxicity, focusing on p53 involvement	• Apoptosis (TUNEL, caspase-3) in labyrinth trophoblast peaked at 6 h and normalized by 48 h<br>• Proliferation (Topo IIα IHC, mitotic index) fell by 3–6 h, recovered by 48 h	Labyrinth zone trophoblastic cell apoptosis, labyrinth thinning; villus/blastocyst necrosis minimal

https://doi.org/10.3390/cimb48010004

							• p53 protein rose at 1–3 h, peaked at 3 h, normalized by 48 h (no p53 mRNA change)<br><br>• p21, cyclin G1, fas mRNAs peaked ~9 h, then declined by 24–48 h	
Kochhar DM et al, 1978 [84]	Single i.p. injection of cytosine-arabinoside (Ara-C) at 2, 10, 25, 50, 100, or 200 mg/kg maternal bodyweight on a specific day of gestation (10.5–12.0 d)	ICR (CD-1) mice, 6–8 weeks old	Pregnant dams received Ara-C at one of six doses on one of four susceptible gestational days (10.5, 11.0, 11.5, 12.0 d)	n ≈ 48 embryos implanted per dose-day cohort (from ~6–8 dams), plus saline-injected and uninjected controls	Embryonic and placental toxicity: limb reduction defects (micromelia, phocomelia, hemmelia, hemimelia, adactyly), embryolethality (resorptions), cytotoxicity to limb-bud mesenchyme, and inhibition of embryonic DNA synthesis	To define how Ara-C perturbs limb development at cellular and molecular levels—specifically, to map stage- and compartment-specific cell death and DNA-synthesis arrest, and to infer differentiation status of affected cells	• Embryolethality was dose- and stage-dependent—100–200 mg/kg fully lethal at 10.5–12.0 d; 2 mg/kg non-teratogenic.<br><br>• Limb-reduction defects displayed strict proximo-distal sequence by treatment stage:<br><br>– 10.5 d → micromelia (shortened long bones, intact digits)<br><br>– 11.0 d → phocomelia (loss of segments with scapula+digits preserved)<br><br>– 11.5 d → hemimelia (radius-ulna missing, humerus+digits intact)<br><br>– 12.0 d → adactyly (digits absent, long bones intact)	• Toluidine-blue micrographs: widespread necrotic mesenchymal cells (osmiophilic debris) in distal limb bud at 11.5–12.0 d; thinning of distal mesenchyme correlating to future bone loss.<br><br>• TEM: pyknotic nuclei, cytoplasmic condensation, phagocytosis of dead cells.<br><br>• AER abnormalities: thickening, pseudoglandular morphology and epithelial folding at 12–24 h post-dose.

							• Ara-C dose-dependently suppressed embryonic DNA synthesis to 11–55% of control at 2 h post-dose, with full (or overshoot) recovery by 24 h except at 2 mg/kg dose (64% recovery).<br><br>• Cytotoxicity localized to high-proliferation mesenchyme—AER and precartilage zones largely spared; necrosis evident by 2–4 h, peaks by 4 h, resolved by 24 h.<br><br>• Simultaneous CdR (8× Ara-C) fully protected against embryolethality and long-bone defects but induced high polydactyly (45–63%) in rescued limbs.	
Manson JM et al, 1977 [85]	Single maternal i.p. dose of 40 mg/kg cytosine arabinoside on gestational days 10–12	Pregnant Swiss-Cox albino mice (ICR strain), ~20–25 g	Dams injected with 40 mg/kg Ara-C at one of three time points per day on GD 10–12; controls saline or untreated	n ≈ 6–9 litters per time	Limb teratogenesis (adactyly, blisters)	To develop an in vitro mouse limb bud culture assay that reproduces Ara-C–induced malformations and to compare Ara-C vs noncytotoxic analogues	In vivo: 40 mg/kg Ara-C on GD10–12 produced blisters + adactyly in 60–100% of limbs; hindlimbs more sensitive<br><br>• Ara-C dose-dependently inhibited 3H-thymidine (to 16% at 10 µg/ml) and 35SO4 uptake;	Distal limb mesenchymal cell necrosis in vivo and in culture; blister corresponds to ectoderm overlying necrotic mesenchyme

https://doi.org/10.3390/cimb48010004

						Ara-U and Ara-HX had no effect on morphology or label uptake	
Rahman ME et al, 1994 [86]	Single maternal i.p. dose of 5 mg/kg cytosine arabinoside on gestational day 10.5	Pregnant Jcl:ICR mice, 10–16 weeks old, ~35 g	• Treated dams: 125 newborns examined<br>• Control dams: 92 newborns examined	Carpal and tarsal bone anomalies (fusion, absence, deformation) in postnatal pups	To determine whether a teratogenic Ara-C dose that causes digital defects also induces anomalies of carpal/tarsal bone development	• 46% forelimb and 25% hindlimb oligodactyly after Ara-C<br>• Carpal anomalies in 58% pups vs 33% controls; tarsal anomalies in 77% vs 14% controls<br>• Fusion: C4–C6, C5–C6; T6–T8, T6–T7–T8; Absence: C5 (56%); Deformation: large C6–C7; small C4<br>• Digital defects and carpal/tarsal anomalies were highly correlated	Permanent absence, fusion and deformations of specific carpal/tarsal ossifications; no nodal cartilage present for missing bones
Rahman ME et al, 1996 [87]	Single maternal i.p. dose of 0.5, 1.0 or 2.0 mg/kg cytosine-arabinoside (Ara-C) on gestational day 10.5 (2130 h)	Pregnant Jcl:ICR mice, 10–16 weeks old, ~35 g	– Three teratogen arms, each with three dose-levels:<br>• Ara-C: 0.5, 1.0, 2.0 mg/kg i.p. (n dams per dose not specified; total PN pups per Ara-C arm ≈112)<br>• Mitomycin-C: 1.0, 2.0, 4.0 mg/kg i.p. (n pups ≈98)<br>• Busulfan: 10, 50, 100 mg/kg p.o. (n pups ≈122)<br>– Controls: untreated and 0.5% CMC vehicle (n pups ≈63)	Postnatal carpal and tarsal bone anomalies (fusion, absence, deformation) as endpoints of limb teratogenesis	To test whether carpal/tarsal ossifications are unusually sensitive targets for antiproliferative teratogens compared to digital elements in mice	• Ara-C at 0.5–2 mg/kg induced carpal bone anomalies in 18.8–88.2% of forelimbs (vs 33% controls) and tarsal anomalies in 24.6–71.5% of hindlimbs (vs 14% controls)<br>• Digit anomalies (metacarpal/phalangeal) were <9% at any Ara-C dose, demonstrating greater sensitivity of carpal/tarsal elements	Permanent fusion, absence or deformation of specific carpal (C5–C6) and tarsal (T6–T8) ossifications without cartilage primordia in their locations

https://doi.org/10.3390/cimb48010004

							• Nearly all limbs with digital defects also had carpal/tarsal anomalies, but carpal/tarsal anomalies occurred independently of digital defects	
Chiang H et al, 1995 [88]	Single i.p. dose of 10 mg/kg cytosine arabinoside on gestational day 9	Pregnant Swiss Webster mice, ~22 ± 2 g	Controls, PMF only, Ara-C only, and PMF + Ara-C groups	n = 21–24 dams per group (89 total)	Teratogenicity: cleft palate (CP), cleft lip (CL), skeletal variations, resorptions, fetal death	To test whether pulsed 15.6 kHz 40 μT magnetic fields (4 h/day GD6–17) enhance ara-C teratogenicity	• Ara-C increased resorptions (19.5% vs 5.3% control) and fetal death; PMF alone did not<br><br>• CP/CL rates: 2.6% control, 14.9% PMF, 26.1% Ara-C, 49.0% PMF + Ara-C (P<0.05 vs control; PMF + Ara-C > Ara-C alone P<0.005)<br><br>• Minor skeletal variations rose with Ara-C (41%–54%); PMF alone modestly increased variations (33% vs 30% control), not significant	N/A
Chiba K et al, 1996 [89]	Single intraperitoneal injection of cytosine arabinoside (Ara-C) at 5.0 mg/kg or 7.5 mg/kg administered on one of three gestational days (GD): 8, 9.5 or 11	Jcl:ICR mice virgin females, 8–24 weeks old, ~35 g	• Control: untreated (9 dams, 97 pups)<br><br>• GD 8: 5.0 mg/kg (8 dams, 97 pups); 7.5 mg/kg (8 dams, 95 pups)<br><br>• GD 9.5: 5.0 mg/kg (9 dams, 101 pups); 7.5 mg/kg (19 dams, 122 pups)		Prenatal Ara-C–induced anomalies of the neonatal hip joint and hindlimb skeleton	To induce hip-joint anomalies in mice by in-utero Ara-C exposure and assess their relationship with adjacent hindlimb bone defects	• Hip-joint anomalies appeared only in the 7.5 mg/kg GD 9.5 group (~30% incidence)<br><br>• Types of hip anomalies:<br><br>– Femoral-shaft dysplasia<br>– Pseudoarthrosis of femur or coxal bone	• Dysplastic/absent ossification centers in femur and pelvis<br><br>• Pseudoarthrosis (non-union) at femoral head, shaft, coxal bones

https://doi.org/10.3390/cimb48010004

			• GD 11: 5.0 mg/kg (8 dams, 87 pups); 7.5 mg/kg (9 dams, 85 pups)			– Femoral-head dysplasia<br>– Acetabular dysplasia<br>– Fusion between femoral head & acetabulum<br><br>• All hip lesions co-occurred with preaxial hyperplasia of hind paws and lower-leg defects<br><br>• Paradox: oligodactyly was common in pups without hip anomalies (23%) but absent in those with hip anomalies<br><br>• Lower-leg anomalies (tibia/fibula non-fusion or fusion, dysplasia) also peaked at GD 9.5 (both doses) and GD 11 (7.5 mg/kg)<br><br>• No shoulder-joint anomalies, though radial-head dysplasia appeared at GD 9.5	• Tibia–fibula fusion proximally; non-fusion distally<br><br>• Preaxial polydactylies/triphalangeal toes and tarsal-bone deformities
Ritter EJ et al, 1971 [90]	Single maternal i.p. dose on gestational day 12 of 25, 50, 100 or 200 mg/kg cytosine arabinoside (Ara-C)	Pregnant Wistar rats, mated to yield embryos at 12 days gestation; Dams weighed ~200–250 g	• Ara-C alone: groups at 25, 50, 100, 200 mg/kg (n dams per dose not given; total implants ~74–101 each)<br><br>• Ara-C + thymidine + partial hysterectomy: same four	• Embryonic malformations (skeletal and external), resorptions, fetal death and	To correlate Ara-C teratogenicity (malformation, lethality, growth deficit) with the kinetics and magnitude of DNA-synthesis	• Dose-dependent embryolethality and malformations: 50–200 mg/kg → increasing resorptions, deaths and growth retardation	Day 20 fetuses showed limb malformations (digital, skeletal) and general growth retardation in a dose-dependent manner

			Ara-C dose groups (total implants ~45–79 each)<br><br>• Untreated controls: pooled historical data, ~477 implants	growth retardation<br><br>• Inhibition of embryonic DNA synthesis	inhibition in embryos	• Ara-C reduced embryonic DNA synthesis to <25% of control within 1 h at all doses; low doses (25 mg/kg) recovered by 24 h, high dose (200 mg/kg) still depressed at 27 h<br><br>• Cumulative DNA-synthesis depression (area under curve) best predicted teratogenic severity	
Kasubuchi Y et al, 1977 [41]	30 mg cytosine arabinoside per kg body-weight, intraperitoneal	ICR-JCL mice	Controls: 12 pregnant mice (4 received ^3H-TdR only)<br><br>Single dose on gestational day (GD) 13⅓ (Group 1)<br><br>Two doses—GD 13⅓ at 18:00 h and GD 14 at 10:00 h (Group 2)<br><br>Autoradiography subgroups (Group 3a–c) also received one 30 mg/kg dose on GD 13⅓:<br><br>3a (n = 2): ^3H-TdR→Ara-C→sacrifice at 6 h<br><br>3b (n = 2): Ara-C→^3H-TdR→sacrifice at 6 h	Transplacental dysgenetic hydrocephalus	To characterize how Ara-C's inhibition of DNA synthesis in the embryonic ventricular (matrix) zone disrupts neurogenesis and produces hydrocephalic malformation	Within 3–6 h post-Ara-C: pyknotic nuclei and debris span the deepest matrix layer; mitoses abolished<br><br>12–24 h: marked matrix destruction, rosette remnants, migratory-zone debris; mitoses reappear in remaining rosettes<br><br>Autoradiography: S-phase cells in the matrix layer are selectively killed; surviving matrix cells become synchronized at S-phase<br><br>Pups from Group 2 exhibit:	Embryonic matrix zone: extensive cell loss, cavitation, debris<br><br>Postnatal cortex: parieto-temporal cortex reduced to membranous remnant; ependymal denudation of lateral ventricles; cystic lesions under thin skull<br><br>Rostral cortex & brainstem largely spared

			3c (n = 2): Ara-C→^3H-TdR (next day)→sacrifice 1 h later			• Severe dilatation of lateral ventricles (parieto-occipital > frontal)<br>• Rapid cranial vault expansion; scanty scalp hair<br>• Death by postnatal day 35<br>• Normal brainstem; absent corpus callosum; patent aqueduct but moderately enlarged 4th ventricle	
Adlard BP et al, 1975 [42]	• Prenatal: single i.p. dose 50 mg/kg on gestational day 14<br><br>• Postnatal: single i.p. dose 250 mg/kg on postnatal day 5	Lister Black and White hooded rats	Prenatal: Controls; Ara-C 50 mg/kg; Ara-A 50 mg/kg; Ara-A 1,000 mg/kg (n per group not specified)<br><br>Postnatal single dose: Controls; Ara-C 50 mg/kg; Ara-C 250 mg/kg; Ara-A 50 mg/kg; Ara-A 250 mg/kg (2 males/litter × 6 litters)<br><br>Postnatal multiple dose: Controls; Ara-A 250 mg/kg × 4 days; Ara-A 1,000 mg/kg × 4 days (n = 6 litters)	Impaired brain growth (whole brain, cerebellum) and adult learning deficits	To compare Ara-C vs Ara-A on rat brain growth/development and behavior when given prenatally or postnatally	• Prenatal Ara-C 50 mg/kg → 17% ↓ brain weight at birth, 14% ↓ body weight; normal brain/body in Ara-A<br><br>• Postnatal Ara-C 250 mg/kg → significant ↓ cerebellum growth vs whole brain; no effect on Ara-A<br><br>• Adult offspring of prenatal Ara-C→ impaired T-maze learning vs controls (80% more errors)	N/A
Elmer GI et al, 2004 [43]	Cytosine arabinoside 30 mg/kg i.p. on embryonic days 19.5 and 20.5 (two injections)	Pregnant Sprague–Dawley rats	• Three cohorts of dams:<br><br>– Cohorts 1–2: 5 saline-, 10 Ara-C–injected dams each<br><br>– Cohort 3: 8 saline-, 12 Ara-C–injected dams	Disruption of sensorimotor gating (prepulse inhibition of acoustic startle)—a putative model for	To determine whether late-embryonic Ara-C exposure produces adult-onset PPI deficits and enhances vulnerability to	• PND 35: no change in baseline startle or PPI<br><br>• PND 56 (adult):<br><br>– Ara-C rats: ↓ startle amplitudes and ↓ PPI versus controls	No gross lesions—subtle, variable disorganization of hippocampal CA2/3 pyramidal cell layer; CA1 lamination intact but slightly thinner

					schizophrenia-like deficits	PPI disruption by dopaminergic (apomorphine) or glutamatergic (PCP) challenge	– Apomorphine: disrupted PPI in controls (all prepulse intensities) but did not further reduce PPI in Ara-C rats<br><br>– PCP: disrupted PPI in controls; Ara-C rats showed no differential sensitivity<br><br>• Ara-C litters had fewer surviving male pups and modest reductions in body (–15%) and brain weight (–5–6%)	
Cano F et al, 2008 [120]	Cytarabine 2 g/m2 i.v. once daily × 5 days	Mll;Enl;Lmo2-Cre translocator mice, 3–4 weeks old	• Mll;Enl;Lmo2-Cre leukemia mice: vehicle (n=3), Ara-C 100 mg/kg (n=5)<br><br>• Dose-ranging: 3.5, 30, 100 mg/kg Ara-C, two 4-day courses separated by 3 d (n≥4 each)<br><br>• Nonleukemic Mll;Enl: vehicle or 100 mg/kg Ara-C		Response of myeloid leukemia to Ara-C (tumor cytoreduction & remission)	To validate Mll-Enl translocator mouse as a preclinical model by testing cytarabine efficacy	• In vivo: 100 mg/kg Ara-C → rapid granulocyte clearance to normal levels, spleen size normalization, extended survival<br><br>• Lower doses (30 mg/kg) also effective; 3.5 mg/kg insufficient → relapse<br><br>• Flow: Mac-1/Gr-1+ blasts ↓→1–2% while B/T-cell populations recovered	N/A
Ritter EJ et al, 1973 [91]	Ara-CP palmitate (long-acting cytarabine) 200 mg/kg i.p. once	Wistar rats, timed-pregnant	Each timepoint: 6 litters Ara-CP, 6 litters HU or	n = 6 per group	Teratogenic limb defects from DNA	To correlate timing of DNA synthesis inhibition, cell death and	• Ara-CP caused delayed/extended DNA synthesis inhibition (to E15-17) vs Ara-C or HU	N/A

https://doi.org/10.3390/cimb48010004

	at gestation day 12		Ara-C, 2 litters controls		synthesis inhibition	timing of limb malformations (ectrodactyly)	• Peak cell death occurred 48–72 h post Ara-CP vs 3–5 h HU or ~23–29 h Ara-C<br>• Limb ectrodactyly pattern correlated with time of peak cell death (more severe with Ara-CP)	
Scott WJ et al, 1975 [92]	Single intraperitoneal dose of cytosine arabinoside (ara-C) at 100 mg/kg body weight, given on either day 10 or day 11 of gestation (depending on which limb was targeted).	Pregnant Royalhart-stock rats (Wistar-derived), timed at gestational day 10 or 11	Groups treated at different times (9 AM, 5 PM on day 10 for forelimb; 9 AM, 5 PM, 9 PM on day 11 for hindlimb).	at least 3 embryos per time-point	Teratogenic limb malformations (polydactyly) and cytotoxic effects on limb-bud mesoderm/ectoderm	To elucidate how an antiproliferative, cytotoxic agent (ara-C) can paradoxically induce preaxial polydactyly in rat embryos	Peak hindlimb polydactyly (85% of survivors) when 100 mg/kg ara-C was given 9 PM day 11.<br>Forelimb polydactyly over a broader window (day 10, 9 AM–9 PM).<br>Treated embryos lost the normal parallelism of meso-/ectoderm proliferation and exhibited synchronized S-phase peaks	• Increased mesodermal cell degeneration (pycnosis/karyorrhexis).<br>• Thickened preaxial ectodermal ridge with necrotic foci.<br>• Absence of the normal preaxial ectodermal cell-death zone seen in controls.<br>• Dilated marginal sinus in limb bud ectoderm
Goto T et al, 1987 [93]	single intraperitoneal injection of cytosine arabinoside at one of three dose levels (2.5, 5 or 10 mg/kg)	Virgin female Jc1:ICR mice, 7–10 weeks old and weighing 25–35 g	• Day 9.5 at 5 mg/kg (32 litters)<br>• Day 9.5 at 10 mg/kg (26 litters)<br>• Day 10.5 at 2.5 mg/kg (23 litters) • Day 10.5 at 5 mg/kg (23 litters)		Digit malformations in the fetuses—specifically polydactyly, syndactyly and	To define dose- and developmental stage–related sex differences in the incidence and pattern (forelimb vs.	• Overall, digit anomalies were more frequent after day 10.5 dosing than day 9.5, and hindlimbs were more affected than forelimbs.	N/A

	administered once on either gestational day 9.5 or day 10.5		• Day 10.5 at 10 mg/kg (26 litters)		oligodactyly (the latter two combined as reduction defects)	hindlimb; preaxial vs. postaxial) of Ara-C-induced digit anomalies	• With 10 mg/kg on day 9.5, hindlimb oligodactyly was significantly higher in male fetuses (≈45 %) than in females (≈29 %).<br>• At 5 mg/kg on day 10.5, male fetuses showed higher oligodactyly in both fore- and hindlimbs, whereas female fetuses exhibited more polydactyly in the hindlimbs.<br>• Increasing dose shifted the lesion pattern from extra digits toward digit loss, a shift more pronounced in males at the mid-level dose.	
Endo A et al, 1987 [94]	A single intraperitoneal injection of cytosine arabinoside at 5 mg/kg body weight, given once on day 11 of gestation	Timed-pregnant CD-1 (ICR) mice, 7–10 weeks old, ~25 g at mating	• Control lighting (06:00–18:00), Ara-C at 10:00<br>• Control lighting, Ara-C at 16:00<br>• Progressive phase delay (light shifted daily 2 h later, dg 2–7), Ara-C at 10:00	12–18 pregnant dams each	Embryotoxicity/teratogenicity manifesting as digit anomalies (polydactyly, oligodactyly) and cleft palate	To test whether the teratogenic effects of Ara-C differ between light versus dark phases by using a progressive phase-shift of the dam's light–dark cycle	• Shifting the dam's light–dark cycle (either delay or advance) had no effect on baseline pregnancy outcome or fetal growth.<br>• Ara-C produced a high rate of digit defects and moderate cleft palate across all six groups.<br>• No significant difference in overall malformation rate between light-phase versus dark-phase dosing when	Only gross and skeletal-staining identification of digital malformations and cleft palate were reported; no additional microscopic pathology was described.

			• Progressive phase delay, Ara-C at 16:00<br>• Progressive phase advance (light shifted daily 2 h earlier, dg 2–6), Ara-C at 10:00<br>• Progressive phase advance, Ara-C at 16:00			comparing control, phase-delay and phase-advance groups.<br>• However, comparing 10:00 versus 16:00 treatments (regardless of lighting schedule) revealed fewer cleft palates but more oligodactyly at the later time, indicating a within-day stage-dependence of susceptibility, not a true light/dark effect.	
Rahman ME et al, 1995 [95]	Single intraperitoneal injection of cytosine arabinoside (Ara-C) at 5 mg/kg body weight, administered once on gestational day 9.5, 11.0 or 12.5	Virgin female Jc1:ICR mice, 10–16 weeks old and weighing approximately 35 g at mating	• Ara-C on dg 9.5: 12 dams, 114 newborns examined<br>• Ara-C on dg 11.0: 15 dams, 201 newborns examined<br>• Ara-C on dg 12.5: 12 dams, 160 newborns examined	Skeletal malformations of the carpal (wrist) and tarsal (ankle) bones, and associated digit anomalies (polydactyly, oligodactyly)	To define the gestational-stage specificity of Ara-C–induced anomalies in mouse carpal and tarsal bones and to assess how those anomalies correlate with digit defects	• Stage-dependent patterns emerged: all three exposure days yielded high rates of carpal/tarsal fusions, but bone absence and deformity were seen only when Ara-C was given on dg 11.0.<br>• Common fusion types varied by day: dg 9.5 favored C6–C7 and T4–T7 fusions, dg 11.0 showed C3–I Mc and T6–T7–T8 fusions, dg 12.5 principally yielded T5–T6 fusion.<br>• Carpal/tarsal anomalies occurred even where digits appeared normal, but every limb with a metacarpal/metatarsal or phalangeal anomaly also	N/A

https://doi.org/10.3390/cimb48010004

						showed at least one carpal/tarsal defect, indicating greater sensitivity of carpal/tarsal elements and a broader critical period than for digits.	
Chaube S et al, 1968 [96]	Single intraperitoneal injection of ara-C on one day of gestation. Doses ranged from 2.5 up to 900 mg/kg applied on days 5–12 (The optimal teratogenic dose was 50 mg/kg on day 10, 100 mg/kg on day 11 and 150 mg/kg on day 12)	Timed-pregnant female CF Wistar rats, weighing 180–250 g at mating	• Ara-C alone: 109 dams injected once (2.5–900 mg/kg) on days 5–12.<br>• Ara-C + CdR: 100 dams on day 12 received 150 mg/kg ara-C plus CdR (5–600 mg/kg) either simultaneously (35 rats) or at intervals up to 360 min apart (65 rats). Controls: 20 dams with 150 mg/kg ara-C alone; 9 with CdR alone.<br>• Ara-C + other nucleosides: 38 dams on day 12 received 150 mg/kg ara-C plus either dCMP, CMP, CDP, CR or TdR (100–1,460 mg/kg); 28 dams received those alone.	Teratogenic malformations in the fetus: cleft palate and lip, encephalocele, limb and tail deformities (hemimelia, phocomelia), digit anomalies, and defects of ossification in skull, ribs and vertebrae	(1) To chart the stage- and dose-dependent teratogenicity of ara-C in the rat embryo (2) To test whether deoxycytidine (CdR) or related nucleotides can prevent ara-C–induced malformations	• Ara-C is nonteratogenic when given on dg 5–9, but teratogenic on dg 10–12 in a sharp dose window. Optimal teratogenic doses (day 12:150 mg/kg) produced 70–90% malformed survivors (vs. <10% in controls).<br>• Malformations included cleft palate/lip, encephalocele, clubbed and retarded limbs, ectro-/polydactyly, incomplete ossification of skull, fused ribs and vertebrae.<br>• CdR (600 mg/kg) completely prevented all ara-C–induced malformations when given up to 20 min before or up to 10 min after a 150 mg/kg ara-C dose; protection waned thereafter (64% at 120 min, <10% by 360 min). Lower CdR doses gave partial protection.	Cleared-stained skeletons revealed incomplete/calvarial ossification, skull–facial bone hypoplasia, fused and split ribs and vertebrae, distorted sternebrae, limb bone agenesis or malalignment, and digital duplications/absence.

https://doi.org/10.3390/cimb48010004

	• dCMP matched CdR's efficacy on a mmol/kg basis. CMP, CDP, CR and TdR failed to protect and in some cases increased fetal mortality.<br><br>• ^{3}H-ara-C assays showed ~30% of the dose excreted in urine over 24 h, minimal deamination to ara-U, and only ~2.5% of radioactivity in embryonic DNA (97.5% in TCA-soluble fraction). Kinase activity was high in embryo vs. maternal liver; deaminase activity was negligible in both.
